# Gene Expression Analysis of Peripheral Cells for Subclassification of Pediatric Inflammatory Bowel Disease in Remission

**DOI:** 10.1371/journal.pone.0079549

**Published:** 2013-11-18

**Authors:** Pieter P. E. van Lierop, Sigrid M. Swagemakers, Charlotte I. de Bie, Sabine Middendorp, Peter van Baarlen, Janneke N. Samsom, Wilfred F. J. van IJcken, Johanna C. Escher, Peter J. van der Spek, Edward E. S. Nieuwenhuis

**Affiliations:** 1 Department of Paediatrics, Erasmus MC - Sophia Children’s Hospital, Rotterdam, The Netherlands; 2 Department of Bioinformatics, Erasmus MC, Rotterdam, The Netherlands; 3 Department of Genetics, Erasmus MC, Rotterdam, The Netherlands; 4 Cancer Genomics Centre, Erasmus MC, Rotterdam, The Netherlands; 5 Center for Biomics, Erasmus MC, Rotterdam, The Netherlands; 6 Top Institute Food and Nutrition, Wageningen, The Netherlands; 7 Department of Paediatric Gastroenterology, Wilhelmina Children’s Hospital, UMC Utrecht, Utrecht, The Netherlands; CWRU/UH Digestive Health Institute, United States of America

## Abstract

**Objective:**

In current clinical practice, optimal treatment of inflammatory bowel disease (IBD) aims at the induction and maintenance of clinical remission. Clinical remission is apparent when laboratory markers of inflammation are normal and clinical symptoms are absent. However, sub-clinical inflammation can still be present. A detailed analysis of the immune status during this inactive state of disease may provide a useful tool to categorize patients with clinical remission into subsets with variable states of immune activation.

**Design:**

By using Affymetrix GeneChips, we analysed RNA gene expression profiles of peripheral blood leukocytes from pediatric IBD patients in clinical remission and controls. We performed (un)supervised clustering analysis of IBD-associated genes and applied Ingenuity® pathway software to identify specific molecular profiles between patients.

**Results:**

Pediatric IBD patients with disease in clinical remission display heterogeneously distributed gene expression profiles that are significantly distinct from controls. We identified three clusters of IBD patients, each displaying specific expression profiles of IBD-associated genes.

**Conclusion:**

The expression of immune- and IBD-associated genes in peripheral blood leukocytes from pediatric IBD patients in clinical remission was different from healthy controls, indicating that sub-clinical immune mechanisms are still active during remission. As such, RNA profiling of peripheral blood may allow for non-invasive patient subclassification and new perspectives in treatment regimes of IBD patients in the future.

## Introduction

Inflammatory bowel disease (IBD) is known as a chronic inflammatory disease of the intestine. Although multiple treatment options have been used to control inflammation, to date no curative interventions have been described. Therefore, the major aim of treatment is the induction and preservation of clinical remission. Furthermore, differences in phenotype, medication responsiveness and associated genetic polymorphisms, illustrate that IBD patients represent a heterogeneous group that may need a novel classification beyond that of Crohn’s disease (CD) and ulcerative colitis (UC). Specifically, genotype, RNA expression, demographic parameters and disease location may help to discriminate subsets within this patient population. Of note, 15% of all IBD cases have disease onset during childhood. [1] Given the distinct disease phenotype and associated pathological changes in children compared to adults, pediatric disease onset can be seen as a specific subset of IBD. [1], [2], [3], [4].

Clinical remission in pediatric IBD is defined as the absence of symptoms of disease, that is expressed as the pediatric Crohn’s disease activity index (PCDAI) or pediatric ulcerative colitis activity index (PUCAI). In both PCDAI and PUCAI, a score <10 defines remission and scores from 10–30 represent mild active disease. [5], [6] The PCDAI describes the overall disease activity and includes laboratory findings, growth and overall wellbeing, whereas the PUCAI only involves clinical signs and symptoms. Although endoscopic disease activity is not part of either PCDAI or PUCAI, the latter has been found to correlate well with colonoscopic appearance in active disease. [6] Correlation of endoscopy and clinical indices during remission is less clear and thus inflammatory processes can be present on a macroscopic as well as a microscopic level in patients who experience no symptoms of active disease. [7] Specifically, subclinical immune activation might still be present despite the absence of clinical symptoms. [8], [9], [10] A detailed analysis of the immune status of patients during remission or mild active disease scores may provide an approach to identify patient subclassifications with subclinical immune activation.

Laboratory markers of inflammation (e.g. CRP and ESR) and fecal calprotectine are associated with (imminent) disease exacerbation. [11], [12] Moreover, severity of disease may be predicted based upon a panel of genetic polymorphisms. [13] However, the usefulness of these parameters for individual patients seems limited. [14] As such, in depth analysis of systemic expression profiles of specific genes involved in the pathogenesis of IBD may help to subclassify IBD patients and may lead to new perspectives in treatment regimes of IBD patients.

To assess the immune status during clinical remission, we analysed gene expression profiles of peripheral blood leukocytes (PBL) obtained from pediatric IBD patients with inactive to mildly active disease as well as control individuals. By using unsupervised clustering analysis, supervised analysis of IBD-associated gene expression as well as Ingenuity® pathway analysis (Ingenuity), we detected notable differences in gene expression profiles when comparing pediatric IBD patients with healthy controls.

These data indicate that the immune status of pediatric IBD patients during clinical remission is not comparable to that of healthy controls and extents to the peripheral blood compartment.

## Materials and Methods

### Patients

A cohort of 45 pediatric IBD patients in clinical remission (24 CD patients and 21 UC patients) with a median age of 16.0 years (IQR 15.0–17.0) were included in this study, as well as 13 controls (Table S1). The control group consisted of orthopaedic patients and did not suffer from inflammatory or intestinal disease at the time of inclusion. This group was recruited during a scheduled control at the hospital.

All patients had received a diagnosis of CD or UC by standard diagnostic work-up according to the Porto criteria. [15] Clinical Disease activity was assessed in the outpatient clinic using the PCDAI and PUCAI indexes for CD and UC, respectively. [5], [6].

None of the IBD patients had overt clinical disease activity upon inclusion. When scoring for clinical remission using the PCDAI and PUCAI, a majority (71%) had a clinical remission score <10. A minor proportion had a mild-active phenotype, scoring 10–30. None of the patients had a score >30 reflecting active disease. Patients received different medication regimes. The majority of CD patients were given azathioprine, whereas most UC patients were given mesalazine. Four patients received prednisolone. Each patient was followed for 18 months for clinical characteristics from the time the blood sample was obtained. Approval for this study was obtained by the Medical Ethics Committee Erasmus MC, Rotterdam, The Netherlands (METC 2007-335). All patients and parents provided their written informed consent to participate in this study.

### Isolation and Quality Control of RNA

Venous blood (2ml) was collected in PAXgene tubes (PreAnalytiX) and stored at –20°C until RNA extraction. Total cellular RNA was extracted using the PAXgene blood RNA kit (Qiagen) according to the manufacturer’s protocol. RNA levels, quality and purity were assessed with the RNA 6000 Nano assay on the Agilent 2100 Bioanalyzer (Agilent). The samples did not show RNA degradation or contamination with DNA.

### Gene Expression Profiling and Quality Control

Samples were analysed by Affymetrix GeneChip Human genome U133 plus 2.0 arrays as described earlier. The GeneChip contains 54,675 probe sets, representing approximately 39,000 genes. Biotinylated antisense cRNA was prepared from 5 µg of total RNA using the one-cycle labelling kit (Affymetrix, Santa Clara, CA). cRNA was hybridized on the GeneChip human genome U133 plus 2.0 array (Affymetrix)45°C for 16–18 hours. Staining, washing and scanning procedures were carried out according to the manufacturer’s protocol (Affymetrix). All GeneChips were visually inspected for irregularities. The R package AffyQC-report was used for quality control and indicated high quality and overall comparability of all samples.

The dataset is located at NIH, gene expression omnibus (GEO). Accession number of repository for expression microarray data: GSE33943.

http://www.ncbi.nlm.nih.gov/geo/query/acc.cgi?acc=GSE33943.

### Data Normalization, Statistical Analysis, and (Un)supervised Analyses

Data were normalized as previously described. [16] In short, raw intensity values of all samples were normalized by background correction and quantile normalization using the version 6.4 Robust Multichip Analysis (Partek). To visualize the correlation between the samples, principal component analysis (PCA) was used. The normalized data file was transposed and imported into OmniViz version 6.0.3 (Biowisdom) for further analysis.

Unsupervised and supervised cluster analyses were performed in Omniviz using Pearson’s correlation in the Correlation View. We used cut-off values for significantly expressed genes with a fold change of 1.5 and a false discovery rate (FDR) of <0.05. Supervised analysis was based on Significance Analysis of Microarrays (SAM). [17] Functional annotation of the SAM results were performed by using Ingenuity.

Group analyses were performed using Pearson’s Rank correlation, student’s T-test, Kruskall - Wallis and Chi-square test when appropriate. P values <0.05 were considered significant.

## Results

### Gene Expression Profiles of Pediatric IBD Patients are Heterogeneously Distributed and Independent of Type of Disease

**Figure 1:**
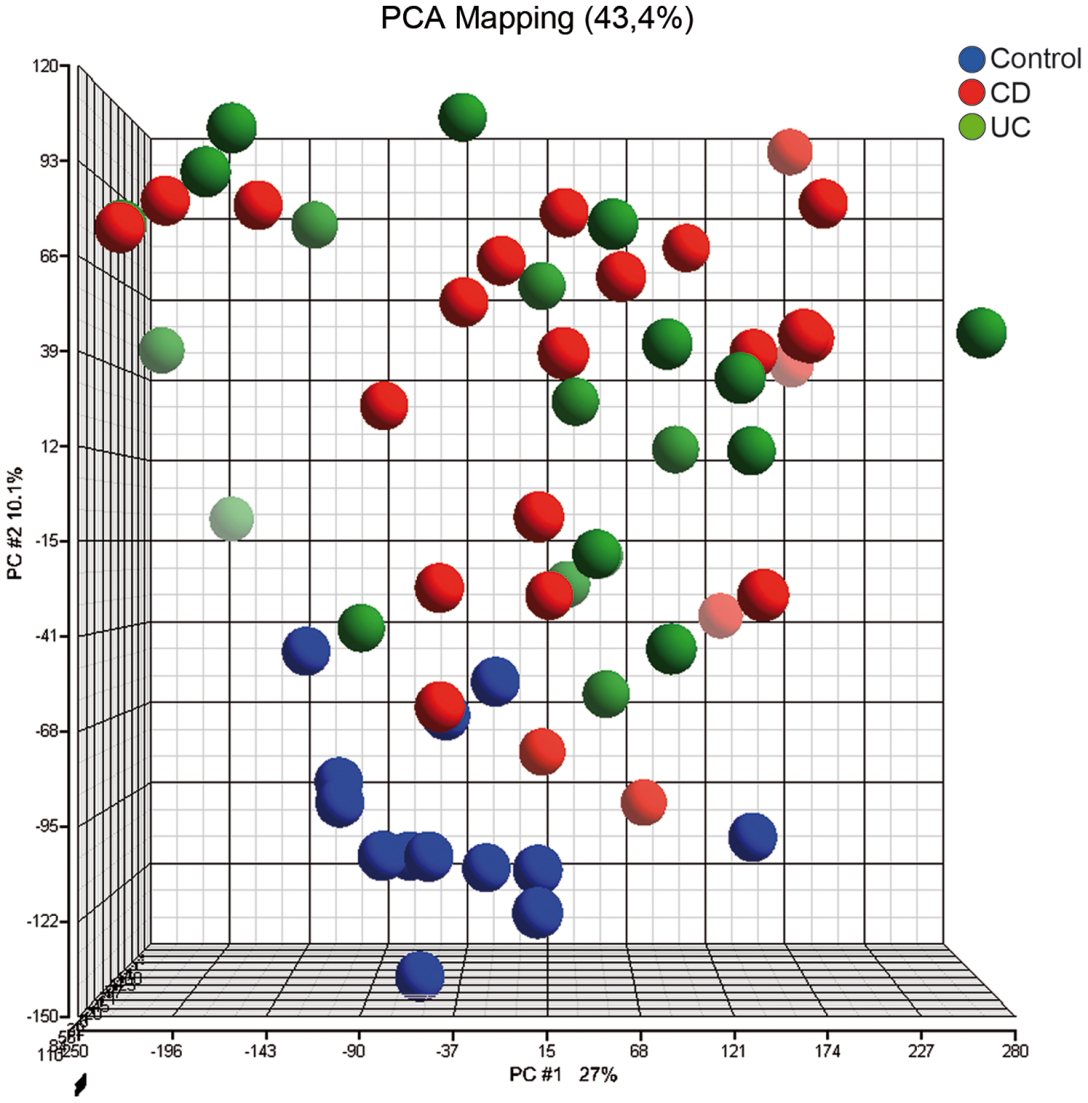
Gene expression profiles of quiescent pediatric IBD patients. Global gene expression profiles of peripheral blood leukocytes were analysed using principal components analysis (PCA). Samples with higher similarity between gene expression profiles cluster more strongly together in the PCA space. Crohn’s disease (red) and ulcerative colitis (green) patients with quiescent disease are not different from each other and form a heterogeneous cluster that is distinct from controls (blue).

Gene expression profiles of PBL from both IBD patients and controls were analysed by using PCA. This type of analysis visualizes clustering of those samples that display comparable expression profiles (Figure 1). In our cohort, control samples appear in a homogeneous cluster (blue) whereas both CD (red) and UC (green) patients clustered together based on overall gene expression profiles. In the PCA some IBD patients were in close vicinity of the controls. However, the majority of the IBD patients appeared at a location that was distinct from the cluster of controls.

As such, IBD patients in clinical remission or mildly active disease are not comparable to healthy individuals based on overall gene expression profiles. Moreover, as we did not observe separate clusters for CD and UC patients, classical sub-categorization into type of disease may not apply for patients in clinical remission.

### IBD Patients can be Subdivided into Three Groups Based on a 3-fold Difference in Gene Expression Level

**Figure 2:**
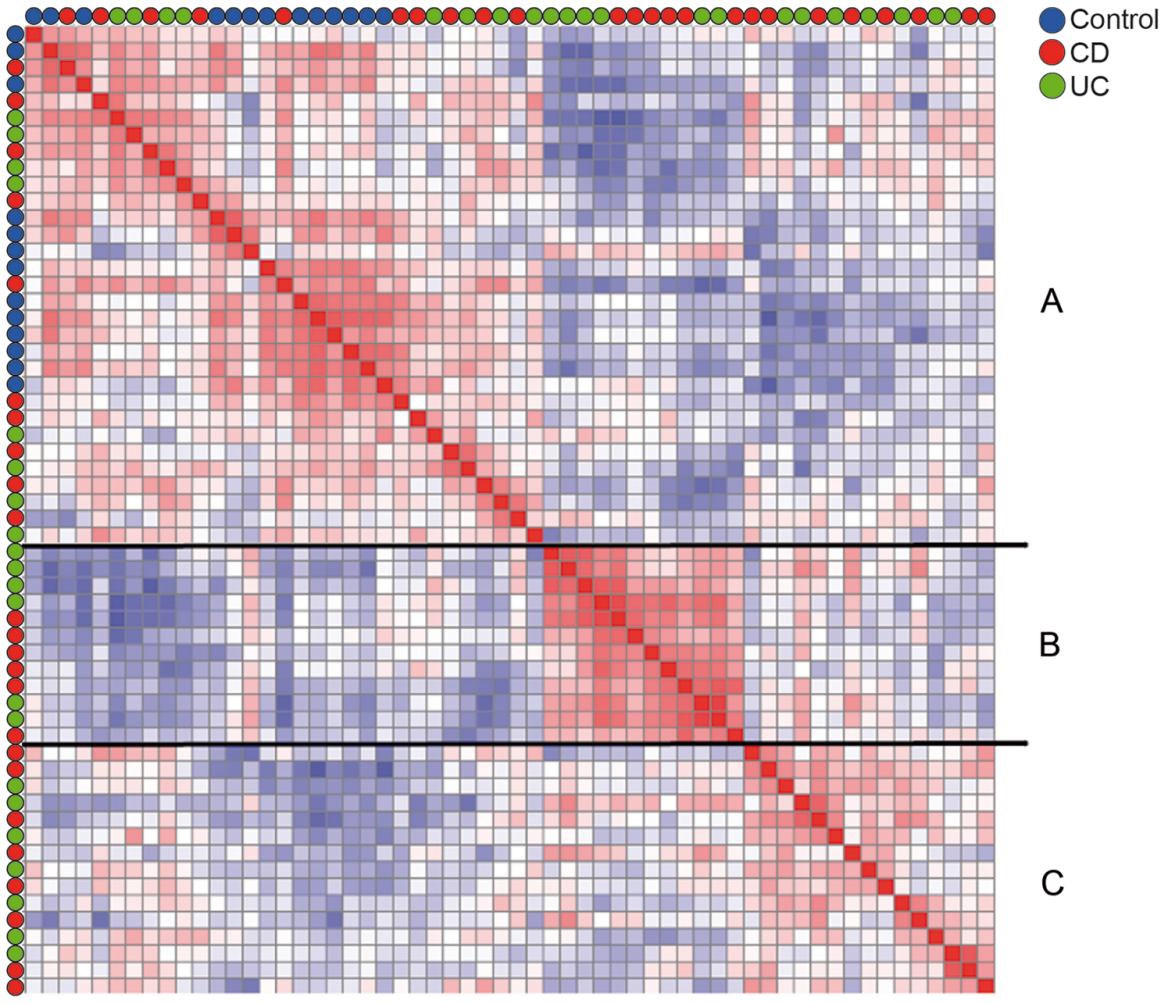
Correlation plot of quiescent IBD patients involving 2957 probe sets. Pair-wise correlations between the samples are depicted in a Pearson’s correlation plot. The colours of the cells relate to Pearson’s correlation coefficient values, with deeper colours indicating higher positive (red) or negative (blue) correlations. This Pearson’s correlation plot revealed three distinct groups (A, B, C). Controls are annotated to group A.

Next, we determined whether the heterogeneous cluster of IBD patients could be subclassified into newly defined subgroups, according to unsupervised ordering of their gene expression profiles. Therefore, we generated a Pearson’s correlation plot that included probe sets with a 3-fold difference in gene expression level relative to the geometric mean of expression, reflecting up- or down-regulation. In total, 2957 records met these criteria and correlation between the samples was analysed on the basis of similarities in gene expression profiles. Samples that show positive correlation in gene expression profiling are indicated in red, whereas negative correlation is illustrated in blue. This Pearson’s correlation plot revealed three distinct groups, which we annotated as group A, B and C. All control samples were located in group A, whereas both CD and UC patients were heterogeneously distributed over all three groups (Figure 2).

To assess whether there may have been underlying (clinical) parameters that influenced the RNA profiling, we compared the disease characteristics for each group in more detail (Table 1). We found no significant difference in clinical parameters (PCDAI or PUCAI scores, CRP, medication, disease type, behaviour and location) between the groups. However, patients in group C displayed a slightly higher ESR (p<0.05). Patients of group A were slightly older compared to the patients of group B (p<0.05), and patients of group C consisted mainly of females (87%) (Table 1). The records that were included for the Pearson correlation plot were located on multiple chromosomes. Only a small percentage of these records (3.5% of all included records genes, 5% in group C) were located on the X or Y chromosome (data not shown).

**Table 1:** Group characteristics.

Group	A		B		C		
	#	%	#	%	#	%	p-value
**N**	18		12		15		
**Female**	7	38.9	6	50.0	13	86.7	**0.02**
**CD**	10	55.6	6	50.0	8	53.3	0.90
**Remission**	16	88.9	6	50.0	10	66.7	0.06
**(PCDAI/PUCAI <10)**							
**Medication**	**#**	**%**	**#**	**%**	**#**	**%**	
**Azathioprine**	9	50.0	7	58.3	4	26.7	
**Inflixmab**	0	0	1	8.3	1	6.7	
**Prednisolone**	1	5.6	1	8.3	2	13.3	
**Mesalazine**	9	50.0	4	33.3	5	33.3	
**Methotrexate**	1	5.6	1	8.3	3	20.0	
**Sulfasalazine**	1	5.6	0	0	2	13.3	
**None**	1	5.6	0	0	1	6.7	
	**Median**	**IQR**	**Median**	**IQR**	**Median**	**IQR**	
**PCDAI/PUCAI**	0	0–5	10	0–20	1	0–16.25	0.23
**ESR (mm/h)**	6	3.75–15.25	5	3–13.25	14	8.75–24.25	**0.04**
**CRP (mmol/l)**	1	1–3.75	1	1–3	2	1–6,5	0.52
**Age (y)**	16,5	15–17	15	14.25–16	16	15–16	**0.05**
**Age onset (y)**	12	11–14	11	6.25–15	14	11–15	0.24
	**Median**	**IQR**	**Median**	**IQR**	**Median**	**IQR**	
**B-cells (CD19+) (%)**	1.15	0.57–2.78	1.16	0.67–2.25	1.44	0.10–3.32	0.55
**Th-cells (CD3+CD4+) (%)**	11.32	9.91–13.54	6.77	4.61–11.55	7.53	3.71–8.88	**0.04**
**Tc-cells (CD3+CD8+) (%)**	4.58	4.03–8.08	3.39	2.35–5.05	4.01	1.42–4.62	0.23
**Granulocytes (%)**	48.82	28.02–57.67	23.68	13.71–33.62	57.08	50.63–64.88	**0.01**
**Monocytes (%)**	4.65	2.81–10.45	2.77	1.19–5.02	8.02	5.70–11.59	**0.01**

Measurement by flow cytometry of immune cells in peripheral blood showed increased percentages of monocytes and granulocytes compared in group C (p<0.001). Medication regimes were comparable between the three groups. (Table 1).

By using unsupervised clustering analysis, we show that IBD patients with clinically inactive disease can be subclassified into three groups with distinct gene expression profiles. CD and UC patients were present in equal percentages within each group and 14 IBD patients clustered together with controls.

### Three Clusters of IBD Patients in Remission Each Display Specific Immunological Expression Profiles

In order to investigate whether the IBD patients of the three subgroups showed differences in IBD-associated immune functions, we analysed the expression of IBD-associated genes (Table S2) in the three separate clusters in a supervised manner. For this analysis we excluded the controls from group A. First, we determined for each group which probe sets showed >1.5 fold higher or lower expression compared to controls. Next, we determined which of these probes sets were significantly different between IBD patients and controls by SAM analysis. Although the majority of IBD patients are in clinical remission or mild active disease, the expression of various IBD-associated genes were found to differ significantly from controls. IBD patients in group A displayed a significantly different expression for 31 IBD-associated probe sets, whereas group B and C contained 151 and 161 probe sets, respectively (Table S3).

Next, we compared the expression levels of several immunological probe sets within the three groups of IBD patients and controls. We found that for group A, only two immunological probe sets were differentially expressed compared to controls and the two other groups. Group B displayed specific gene expression for 75 probe sets, whereas in group C 26 probe sets were distinct from controls as well as from the two other groups (Table S4).

These results indicate that although the clinical phenotype of the IBD patients and controls is comparable, expression levels of IBD-associated genes are significantly different between the IBD patients and controls. Furthermore, by performing supervised analysis between the three groups, as defined in Figure 2, we show that the groups were at least in part divided on the basis of gene expression profiles of IBD-associated immune genes.

### Ingenuity Pathway Analysis Reveals Group-specific Involvement of Immunological Pathways

In order to investigate the possible interaction of the differentially expressed immunological genes, we analysed for each patient group which immunological pathways were associated with these genes by using Ingenuity®. The pathways within groups A, B and C that were distinct from controls involved immunological pathways at all levels of the immune cascade, ranging from pattern recognition to adaptive immune cell activation (Table 2). In all groups, genes of the glucocorticoid receptor signaling pathway showed differential expression compared to controls, while for example the IL-4 signaling pathway was only significantly modulated in groups B and C. By performing Ingenuity pathway analysis, we determined which pathways derived from Table S4 were most distinctive for each group individually (Table 3).

**Table 2:** Top canonical pathways Group A/B/C vs Control.

	Name	p-value	ratio
**A vs Control**	Glucocorticoid Receptor Signaling	1.01^e-23^	16/280 (0.057)
	Role of NFAT in Regulation of the Immune Response	1.75^e-16^	11/194 (0.057)
	Systemic Lupus Erythematosus Signaling	1.11^e-15^	10/150 (0.067)
	iCOS-iCOSL Signaling in T Helper Cells	6.51^e-13^	8/115 (0.07)
	T Cell Receptor Signaling	7.77^e-13^	8/110 (0.073)
**B vs Control**	Glucocorticoid Receptor Signaling	9.26^e-92^	61/280 (0.218)
	Estrogen Receptor Signaling	1.46^e-42^	29/121 (0.24)
	Colorectal Cancer Metastasis Signaling	3.56^e-22^	22/247 (0.089)
	IL-4 Signaling	2.63^e-21^	15/72 (0.208)
	B Cell Receptor Signaling	1.97^e-20^	18/155 (0.116)
**C vs Control**	Glucocorticoid Receptor Signaling	5.66^e-72^	52/280 (0.186)
	IL-4 Signaling	2.65^e-19^	14/72 (0.194)
	Dendritic Cell Maturation	1.60^e-18^	17/173 (0.098)
	RAR Activation	1.26^e-17^	17/178 (0.096)
	Systemic Lupus Erythematosus Signaling	1.41^e-16^	15/150 (0.1)

**Table 3:** Top canonical pathways Group A/B/C vs Group and Controls.

	Name	p-value	ratio
**A vs B,C and controls**	Role of PKR in Interferon Induction and Antiviral Response	4.05^e-03^	1/46 (0.022)
	Fcg Receptor-mediated Phagocytosis in Macrophages and Monocytes	9.21^e-03^	1/104 (0.01)
	Systemic Lupus Erythematosus Signaling	1.24^e-02^	1/150 (0.007)
	Dendritic Cell Maturation	1.44^e-02^	1/173 (0.006)
	Role of NFAT in Regulation of the Immune Response	1.59^e-02^	1/194 (0.005)
**B vs A,C and controls**	Glucocorticoid Receptor Signaling	1.89^e-51^	34/280 (0.121)
	Estrogen Receptor Signaling	9.51^e-28^	18/121 (0.149)
	Colorectal Cancer Metastasis Signaling	4.28^e-14^	13/247 (0.053)
	HMGB1 Signaling	8.82^e-14^	10/98 (0.102)
	LPS-stimulated MAPK Signaling	3.98^e-13^	9/80 (0.112)
**C vs A,B and controls**	Glucocorticoid Receptor Signaling	5.41^e-18^	12/280 (0.043)
	Hepatic Fibrosis/Hepatic Stellate Cell Activation	2.37^e-07^	5/135 (0.037)
	Communication between Innate and Adaptive Immune Cells	5.03^e-05^	3/90 (0.033)
	LXR/RXR Activation	5.51^e-05^	3/86 (0.035)
	Colorectal Cancer Metastasis Signaling	9.92^e-05^	4/247 (0.016)

This in depth analysis indicated that in IBD patients with clinical remission of mildly active disease, active immune processes were still differentially modulated in comparison to the healthy controls. These active processes were shown to participate in distinct, well-known immune-related pathways.

### Supervised Analysis Indicated Differences in Gene Expression Profiles of IBD-associated Genes

Next, we investigated whether the active immune processes found during clinical remission could predict exacerbation or clinical inflammation. We determined gene expression profiles of IBD-associated genes in relation to exacerbations during a one and a half year follow-up from inclusion. Within this time frame, 61% of the CD patients and 76% of the UC patients encountered one or more exacerbations. We did not find differences in the occurrence of exacerbations between groups A, B and C (data not shown).

Supervised analysis of the patients that had one or more exacerbations indicated that multiple IBD-associated genes were differentially expressed in the remission phase, specifically in CD patients that developed an exacerbation compared to CD patients with sustained remission (Table S5). We did not detect any difference in IBD-associated gene expression when we performed a similar analysis for UC patients (data not shown). Furthermore, only three IBD-related genes were differentially expressed between CD and UC patients, whereas seventeen IBD-related genes were differentially expressed in CD patients with ileitis or ileocolitis (74%) at the time of diagnosis compared to CD patients without ileal involvement (Table S5).

## Discussion

Bioinformatics-based integration of molecular and clinical information has proven to be crucial for the discovery of new disease pathways relevant to identify subclasses of various diseases. [16] Using gene expression arrays in IBD, multiple genes have been discovered which are associated with activity of disease, phenotype (e.g. CD and UC) and age of onset of IBD. [18], [19], [20], [21], [22], [23] However, most of these studies were performed using supervised analysis in order to find associated genes for CD compared to UC or for disease activity. This study is, to our knowledge, the first that includes pediatric IBD patients in clinical remission or mild active disease and encompasses unsupervised analyses of gene expression profiles in peripheral blood. By using this approach, we have now revealed substantial differences between non-symptomatic IBD patients and controls and have catalogued and analysed genes that were differentially expressed between IBD patients and healthy persons.

Given the ethical limitations of performing colonoscopy with biopsies in pediatric IBD patients during clinical remission, we decided to focus on expression arrays of PBL. Numerous studies have shown potential immunological differences in peripheral derived immune cells from IBD patients in the active stages of the disease. [21], [24], [25], [26], [27], [28] Using gene expression arrays of PBL, we have shown that the majority of pediatric IBD patients experiencing clinical remission have gene expression profiles that are diverse and distinct from controls (Figure 1). Interestingly, CD and UC patients did not form separate clusters within the PCA, suggesting immunological similarities between these two subsets in this inactive phase of disease. Over the last years multiple SNPs have been found in active IBD patients that are specific for CD and UC. Interestingly, overlap for some of these SNPs has been established between CD and UC. [29], [30].

By using unsupervised analysis on the genes, we have subclassified pediatric IBD patients in remission or mild active disease into three distinct groups (Figure 2). Although non-immune genes were overrepresented in these three groups, we also observed significant differences in immunological gene expression profiles between controls as well as between groups (Tables S3 and S4). The majority of these genes were upregulated when compared to controls, suggesting activation of associated pathways. We performed Ingenuity pathway analysis in order to investigate which pathways were affected by the various differentially expressed genes in the IBD groups and controls. By this method we could identify the associated pathways (Tables 2 and 3) for each gene. Most importantly, genes involved in the glucocorticoid receptor signaling pathway showed enhanced expression in group B and C. As these two groups were most distinct from controls in the Pearson’s correlation plot (Figure 2), we conclude that this pathway plays an important role in the distinction between controls and asymptomatic IBD patients. Continuous activation of the glucocorticoid receptor signaling pathway could indicate that these patients request a more robust anti-inflammatory medication regime in order to preserve remission.

Although all groups had comparable PCDAI/PUCAI scores, patients of group C had a higher ESR and increased amounts of granulocytes and monocytes, suggesting an activated immune system as compared with the other groups. However, the difference is small and absolute numbers of immune cells are all below the threshold.

Partly due to the limited number of patients and the heterogeneous phenotype in our cohort of patients, we could not establish an association with clinical characteristics or duration of disease. Therefore, we are at the moment unable to determine whether the patient classification may help predict the course of disease (e.g. exacerbations) in the future. In a one and a half year follow up of our patients, we did not find an association between the groups or course of disease such as exacerbation. However, supervised analysis of those CD patients that encountered an exacerbation and those with prolonged remission, illustrated differences in gene expression profiles (Table S5). Interestingly, one of these genes, interleukin-8, has been extensively associated with the pathogenesis of CD. [25], [31], [32] Previously, we have observed enhanced interleukin-8 production in buccal epithelial cells of pediatric CD patients, [33] indicating a potential role for this chemokine in the pathogenesis of pediatric CD. Moreover, other genes in this analysis, such as protein tyrosine phosphatase, non-receptor type 2 (PTPN2) and prostaglandin E receptor 4 (PTGER4) have also been associated with active CD. [34], [35] A prospective study that assesses RNA expression longitudinally (at the time of diagnosis, during remission and at the time of exacerbation) may provide answers whether the expression of these and/or other genes can predict disease exacerbation.

In summary, we find that remission in pediatric IBD patients is associated with gene expression profiles that are distinct from healthy persons. We conclude that in the absence of clinical symptoms, various immune-related pathways are still active, suggesting subclinical immunological activation.

Our studies suggest that monitoring of specific gene expression profiles may help identify subclasses of pediatric IBD patients and may deliver novel sets of genes associated with different IBD types and stages. Prospective clinical studies using both clinical disease activity markers as well as “immunological disease activity” using bioinformatics may lead to new perspectives in treatment regimes of IBD patients.

## Supporting Information

Table S1 Patient characteristics.
(DOC)

Table S2 IBD Related genes.
(DOC)

Table S3 Top genes up/down regulated Group A/B/C vs Control.
(DOC)

Table S4 Specific top genes up/down regulated group A/B/C.
(DOC)

Table S5 Supervised analysis.
(DOC)
