# Supplementary material for: Gene Expression Analysis of Peripheral Cells for Subclassification of Pediatric Inflammatory Bowel Disease in Remission
Source: PLoS One. 2013 Nov 18;8(11):e79549. doi: 10.1371/journal.pone.0079549 (PMC3832619; doi:10.1371/journal.pone.0079549)
Supplement: Table S1 — Patient characteristics. (DOC) [file pone.0079549.s001.doc]

**Table S1, Patient characteristics**

|  | **IBD** | **%** |  | **Control** | **%** |
| --- | --- | --- | --- | --- | --- |
| Total | 45 |  |  | 13 |  |
| Male | 19 | 42,2 |  | 7 | 53,8 |
| Female | 26 | 57,8 |  | 6 | 46,2 |
|  |  |  |  |  |  |
| Crohn's disease | 24 | 53,3 |  |  |  |
| Colonic involvement | 18 | 75,0 |  |  |  |
|  |  |  |  |  |  |
| Ulcerative colitis | 21 | 46,7 |  |  |  |
|  |  |  |  |  |  |
|  |  |  |  |  |  |
|  | **Median** | **IQR** |  | **Median** | **IQR** |
| **Age (years)** | 16.0 | 15.0 - 17.0 |  | 13,5 | 12 - 14,75 |
| Age CD | 16.0 | 15.0 - 17.0 |  |  |  |
| Age UC | 15.0 | 15.0 - 16.0 |  |  |  |
|  |  |  |  |  |  |
| **Age of onset (years)** | 13.0 | 11.0 - 15.0 |  |  |  |
| Age of onset CD | 13.0 | 11.0 - 15.0 |  |  |  |
| Age of onset UC | 11.5 | 9,5 - 14,0 |  |  |  |
|  |  |  |  |  |  |
| PCDAI | 1 | 1 - 10 |  |  |  |
| PUCAI | 0 | 0 - 10 |  |  |  |
|  |  |  |  |  |  |
| **ESR (mm/h)** | 8,5 | 4,25 - 15,75 |  |  |  |
| ESR CD (mm/h) | 11 | 5,25 - 16,75 |  |  |  |
| ESR UC (mm/h) | 6 | 3,25 - 14,75 |  |  |  |
|  |  |  |  |  |  |
| **CRP (mmol/l)** | 1 | 1 - 3,75 |  |  |  |
| CRP CD (mmol/l) | 1 | 1 - 6 |  |  |  |
| CRP UC (mmol/l) | 1 | 1 - 2 |  |  |  |
|  |  |  |  |  |  |
|  |  |  |  |  |  |
| **Medication CD** | **#** | **%** |  |  |  |
| Azathioprine | 15 | 65,2 |  |  |  |
| Inflixmab | 2 | 8,7 |  |  |  |
| Prednisolone | 3 | 13,0 |  |  |  |
| Mesalazine | 4 | 17,4 |  |  |  |
| Methotrexate | 5 | 21,7 |  |  |  |
| None | 1 | 4,3 |  |  |  |
|  |  |  |  |  |  |
| **Medication UC** | **#** | **%** |  |  |  |
| Azathioprine | 5 | 23,8 |  |  |  |
| Inflixmab | 0 | 0,0 |  |  |  |
| Prednisolone | 1 | 4,8 |  |  |  |
| Sulfasalazine | 3 | 14,3 |  |  |  |
| Mesalazine | 16 | 76,2 |  |  |  |
| Methotrexate | 0 | 0,0 |  |  |  |
| None | 1 | 4,8 |  |  |  |
