# Supplementary material for: Gene Expression Analysis of Peripheral Cells for Subclassification of Pediatric Inflammatory Bowel Disease in Remission
Source: PLoS One. 2013 Nov 18;8(11):e79549. doi: 10.1371/journal.pone.0079549 (PMC3832619; doi:10.1371/journal.pone.0079549)
Supplement: Table S2 — IBD Related genes. (DOC) [file pone.0079549.s002.doc]

**Table S2, IBD related genes**

| **Gene Title** | **Gene Symbol** | **Location** |
| --- | --- | --- |
| heat shock 70kDa protein 6 (HSP70B') | HSPA6 | chr1q23 |
| chemokine (C-C motif) ligand 5 | CCL5 | chr17q11.2-q12 |
| estrogen-related receptor alpha | ESRRA | chr11q13 |
| mitogen-activated protein kinase 1 | MAPK1 | chr22q11.2|22q11.21 |
| mitogen-activated protein kinase 1 | MAPK1 | chr22q11.2|22q11.21 |
| prune homolog 2 (Drosophila) | PRUNE2 | chr9q21.13 |
| interleukin 12 receptor, beta 1 | IL12RB1 | chr19p13.1 |
| Janus kinase 1 (a protein tyrosine kinase) | JAK1 | chr1p32.3-p31.3 |
| Janus kinase 1 (a protein tyrosine kinase) | JAK1 | chr1p32.3-p31.3 |
| DNA directed RNA polymerase II polypeptide J-related | POLR2J2 | chr7q22.1 |
| polymerase (RNA) II (DNA directed) polypeptide J, 13.3kDa pseudogene /// DNA directed RNA polymerase II polypeptide J-related /// similar to hCG1989139 /// PMS2 postmeiotic segregation increased 2 (S. cerevisiae)-like /// similar to POLR2J4 protein | LOC100134053 /// LOC392713 /// LOC441259 /// POLR2J2 /// POLR2J4 | chr7p13 /// chr7q11.23 /// chr7q22.1 |
| toll-like receptor 4 | TLR4 | chr9q32-q33 |
| interleukin 23 receptor | IL23R | chr1p31.3 |
| interleukin 29 (interferon, lambda 1) | IL29 | chr19q13.13 |
| interleukin 27 | IL27 | --- |
| TAF1 RNA polymerase II, TATA box binding protein (TBP)-associated factor, 210kDa-like | TAF1L | chr9p21.1 |
| chromosome 13 open reading frame 31 | C13orf31 | chr13q14.11 |
| chromosome 13 open reading frame 31 | C13orf31 | chr13q14.11 |
| pseudouridylate synthase 10 | PUS10 | chr2p16.1 |
| mucin 19, oligomeric | MUC19 | chr12q12 |
| TAF5 RNA polymerase II, TATA box binding protein (TBP)-associated factor, 100kDa | TAF5 | chr10q24-q25.2 |
| cytochrome c oxidase II /// zinc finger, FYVE domain containing 20 | COX2 /// ZFYVE20 | chr3p24.3 |
| cytochrome c oxidase II /// zinc finger, FYVE domain containing 20 | COX2 /// ZFYVE20 | chr3p24.3 |
| PDZK1 interacting protein 1 | PDZK1IP1 | chr1p33 |
| insulin-like 3 (Leydig cell) /// Janus kinase 3 (a protein tyrosine kinase, leukocyte) | INSL3 /// JAK3 | chr19p13.1 /// chr19p13.2-p12 |
| phosphoinositide-3-kinase, class 2, alpha polypeptide | PIK3C2A | chr11p15.5-p14 |
| NK2 transcription factor related, locus 3 (Drosophila) | NKX2-3 | chr10q24.2 |
| oxoglutarate (alpha-ketoglutarate) dehydrogenase (lipoamide) | OGDH | chr7p14-p13 |
| oxoglutarate (alpha-ketoglutarate) dehydrogenase (lipoamide) | OGDH | chr7p14-p13 |
| TAF5-like RNA polymerase II, p300/CBP-associated factor (PCAF)-associated factor, 65kDa | TAF5L | chr1q42.13 |
| serglycin | SRGN | chr10q22.1 |
| TAF2 RNA polymerase II, TATA box binding protein (TBP)-associated factor, 150kDa | TAF2 | chr8q24.12 |
| acetyl-Coenzyme A acetyltransferase 1 (acetoacetyl Coenzyme A thiolase) | ACAT1 | chr11q22.3-q23.1 |
| interleukin 16 (lymphocyte chemoattractant factor) | IL16 | chr15q26.3 |
| signal transducer and activator of transcription 5B | STAT5B | chr17q11.2 |
| signal transducer and activator of transcription 5B | STAT5B | chr17q11.2 |
| cyclin-dependent kinase inhibitor 1A (p21, Cip1) | CDKN1A | chr6p21.2 |
| tyrosine aminotransferase | TAT | chr16q22.1 |
| interleukin 28 receptor, alpha (interferon, lambda receptor) | IL28RA | chr1p36.11 |
| lysozyme (renal amyloidosis) | LYZ | chr12q15 |
| chemokine (C-C motif) ligand 5 | CCL5 | chr17q11.2-q12 |
| periostin, osteoblast specific factor | POSTN | chr13q13.3 |
| periostin, osteoblast specific factor | POSTN | chr13q13.3 |
| Polymerase (RNA) II (DNA directed) polypeptide B, 140kDa | POLR2B | chr4q12 |
| polymerase (RNA) II (DNA directed) polypeptide B, 140kDa | POLR2B | chr4q12 |
| mitogen-activated protein kinase 12 | MAPK12 | chr22q13.33 |
| mitogen-activated protein kinase 12 | MAPK12 | chr22q13.33 |
| collagen, type I, alpha 1 | COL1A1 | chr17q21.33 |
| son of sevenless homolog 1 (Drosophila) | SOS1 | chr2p22-p21 |
| V-raf-1 murine leukemia viral oncogene homolog 1 | RAF1 | chr3p25 |
| mucin 12, cell surface associated | MUC12 | chr7q22 |
| mucin 12, cell surface associated | MUC12 | chr7q22 |
| heat shock protein 90kDa alpha (cytosolic), class B member 1 | HSP90AB1 | chr6p12 |
| solute carrier family 16, member 1 (monocarboxylic acid transporter 1) | SLC16A1 | chr1p12 |
| ubiquitin-conjugating enzyme E2I (UBC9 homolog, yeast) | UBE2I | chr16p13.3 |
| TAF11 RNA polymerase II, TATA box binding protein (TBP)-associated factor, 28kDa | TAF11 | chr6p21.31 |
| TAF11 RNA polymerase II, TATA box binding protein (TBP)-associated factor, 28kDa | TAF11 | chr6p21.31 |
| immunoglobulin lambda locus /// immunoglobulin lambda constant 2 (Kern-Oz- marker) /// immunoglobulin lambda variable 7-46 /// immunoglobulin lambda variable 7-43 /// immunoglobulin lambda variable 2-14 | IGL@ /// IGLC2 /// IGLV2-14 /// IGLV7-43 /// IGLV7-46 | chr22q11.1-q11.2 /// chr22q11.2 |
| fibronectin 1 | FN1 | chr2q34 |
| COX17 cytochrome c oxidase assembly homolog (S. cerevisiae) | COX17 | chr3q13.33 |
| alpha-2-macroglobulin | A2M | chr12p13.3-p12.3 |
| v-Ki-ras2 Kirsten rat sarcoma viral oncogene homolog | KRAS | chr12p12.1 |
| v-Ki-ras2 Kirsten rat sarcoma viral oncogene homolog | KRAS | chr12p12.1 |
| acetyl-Coenzyme A acetyltransferase 1 (acetoacetyl Coenzyme A thiolase) | ACAT1 | chr11q22.3-q23.1 |
| MRNA; cDNA DKFZp686D10250 (from clone DKFZp686D10250) /// Hypothetical gene supported by AK128882 | LOC441108 | chr5q31.1 |
| FK506 binding protein 4, 59kDa | FKBP4 | chr12p13.33 |
| FK506 binding protein 4, 59kDa | FKBP4 | chr12p13.33 |
| glycophorin E | GYPE | chr4q31.1 |
| glycophorin E | GYPE | chr4q31.1 |
| Mucin 4, cell surface associated | MUC4 | chr3q29 |
| interleukin 23 receptor | IL23R | chr1p31.3 |
| Janus kinase 2 (a protein tyrosine kinase) | JAK2 | chr9p24 |
| Janus kinase 1 (a protein tyrosine kinase) | JAK1 | chr1p32.3-p31.3 |
| Nuclear receptor coactivator 3 | NCOA3 | chr20q12 |
| immunity-related GTPase family, M | IRGM | chr5q33.1 |
| hypothetical gene supported by AK128882 | LOC441108 | chr5q31.1 |
| mucin 6, oligomeric mucus/gel-forming /// similar to Mucin-6 precursor (Gastric mucin-6) | LOC100133432 /// LOC100133761 /// MUC6 | chr11p15.5-p15.4 |
| SMAD family member 4 | SMAD4 | chr18q21.1 |
| SMAD family member 4 | SMAD4 | chr18q21.1 |
| fatty acid binding protein 6, ileal (gastrotropin) | FABP6 | chr5q33.3-q34 |
| fatty acid binding protein 6, ileal (gastrotropin) | FABP6 | chr5q33.3-q34 |
| Ras-related C3 botulinum toxin substrate 1 (rho family, small GTP binding protein Rac1) | RAC1 | chr7p22 |
| ras-related C3 botulinum toxin substrate 1 (rho family, small GTP binding protein Rac1) | RAC1 | chr7p22 |
| CD74 molecule, major histocompatibility complex, class II invariant chain | CD74 | chr5q32 |
| CD74 molecule, major histocompatibility complex, class II invariant chain | CD74 | chr5q32 |
| Enhancer of polycomb homolog 1 (Drosophila) | IL23A | chr12q13.2 |
| phosphoinositide-3-kinase, regulatory subunit 2 (beta) | PIK3R2 | chr19q13.2-q13.4 |
| FGFR1 oncogene partner | FGFR1OP | chr6q27 |
| solute carrier family 22, member 23 | SLC22A23 | chr6p25.2 |
| solute carrier family 22, member 23 | SLC22A23 | chr6p25.2 |
| serpin peptidase inhibitor, clade E (nexin, plasminogen activator inhibitor type 1), member 1 | SERPINE1 | chr7q21.3-q22 |
| phosphoinositide-3-kinase, class 2, alpha polypeptide | PIK3C2A | chr11p15.5-p14 |
| phosphoinositide-3-kinase, class 2, alpha polypeptide | PIK3C2A | chr11p15.5-p14 |
| SWI/SNF related, matrix associated, actin dependent regulator of chromatin, subfamily a, member 4 | SMARCA4 | chr19p13.2 |
| peroxisome proliferator-activated receptor gamma, coactivator 1 alpha | PPARGC1A | chr4p15.1 |
| chemokine (C-X-C motif) ligand 2 | CXCL2 | chr4q21 |
| phosphoinositide-3-kinase, class 3 | PIK3C3 | chr18q12.3 |
| chromosome 11 open reading frame 30 | C11orf30 | chr11q13.5 |
| Phosphoinositide-3-kinase, class 2, alpha polypeptide | PIK3C2A | chr11p15.5-p14 |
| Phosphoinositide-3-kinase, class 2, alpha polypeptide | PIK3C2A | chr11p15.5-p14 |
| zinc finger protein 365 | ZNF365 | chr10q21.2 |
| TAF10 RNA polymerase II, TATA box binding protein (TBP)-associated factor, 30kDa | TAF10 | chr11p15.3 |
| heat shock protein 90kDa alpha (cytosolic), class B member 1 | HSP90AB1 | chr6p12 |
| prostaglandin E synthase 3 (cytosolic) | PTGES3 | chr12q13.3|12 |
| S100 calcium binding protein A11 | S100A11 | chr1q21 |
| cathepsin A | CTSA | chr20q13.1 |
| secreted protein, acidic, cysteine-rich (osteonectin) | SPARC | chr5q31.3-q32 |
| high-mobility group box 1 | HMGB1 | chr13q12 |
| high-mobility group box 1 | HMGB1 | chr13q12 |
| heat shock 70kDa protein 9 (mortalin) | HSPA9 | chr5q31.1 |
| heat shock 70kDa protein 9 (mortalin) | HSPA9 | chr5q31.1 |
| heat shock 70kDa protein 9 (mortalin) | HSPA9 | chr5q31.1 |
| GNAS complex locus | GNAS | chr20q13.3 |
| annexin A5 | ANXA5 | chr4q26-q28|4q28-q32 |
| enoyl Coenzyme A hydratase 1, peroxisomal | ECH1 | chr19q13.1 |
| heat shock 70kDa protein 1A /// heat shock 70kDa protein 1B | HSPA1A /// HSPA1B | chr6p21.3 |
| heat shock 70kDa protein 1A /// heat shock 70kDa protein 1B | HSPA1A /// HSPA1B | chr6p21.3 |
| peroxiredoxin 6 | PRDX6 | chr1q25.1 |
| peroxiredoxin 6 | PRDX6 | chr1q25.1 |
| nuclear receptor co-repressor 1 | NCOR1 | chr17p11.2 |
| nuclear receptor co-repressor 1 /// chromosome 20 open reading frame 191 /// similar to nuclear receptor co-repressor 1 | C20orf191 /// LOC100133918 /// NCOR1 | chr17p11.2 /// chr20p11.1 |
| nuclear receptor co-repressor 1 /// chromosome 20 open reading frame 191 /// similar to nuclear receptor co-repressor 1 | C20orf191 /// LOC100133918 /// NCOR1 | chr17p11.2 /// chr20p11.1 |
| nuclear receptor co-repressor 1 | NCOR1 | chr17p11.2 |
| creatine kinase, brain | CKB | chr14q32 |
| signal transducer and activator of transcription 1, 91kDa | STAT1 | chr2q32.2 |
| FK506 binding protein 4, 59kDa | FKBP4 | chr12p13.33 |
| FK506 binding protein 4, 59kDa | FKBP4 | chr12p13.33 |
| major histocompatibility complex, class I, E | HLA-E | chr6p21.3 |
| major histocompatibility complex, class I, E | HLA-E | chr6p21.3 |
| tetraspanin 3 | TSPAN3 | chr15q24.3 |
| tetraspanin 3 | TSPAN3 | chr15q24.3 |
| GNAS complex locus | GNAS | chr20q13.3 |
| hypoxia-inducible factor 1, alpha subunit (basic helix-loop-helix transcription factor) | HIF1A | chr14q21-q24 |
| CD9 molecule | CD9 | chr12p13.3 |
| annexin A1 | ANXA1 | chr9q12-q21.2|9q12-q21.2 |
| tyrosine 3-monooxygenase/tryptophan 5-monooxygenase activation protein, eta polypeptide | YWHAH | chr22q12.3 |
| TAF7 RNA polymerase II, TATA box binding protein (TBP)-associated factor, 55kDa | TAF7 | chr5q31 |
| dual specificity phosphatase 1 | DUSP1 | chr5q34 |
| dual specificity phosphatase 1 | DUSP1 | chr5q34 |
| cytochrome c-1 | CYC1 | chr8q24.3 |
| major histocompatibility complex, class II, DP beta 1 | HLA-DPB1 | chr6p21.3 |
| v-raf-1 murine leukemia viral oncogene homolog 1 | RAF1 | chr3p25 |
| oxoglutarate (alpha-ketoglutarate) dehydrogenase (lipoamide) | OGDH | chr7p14-p13 |
| interferon induced transmembrane protein 2 (1-8D) | IFITM2 | chr11p15.5 |
| chaperonin containing TCP1, subunit 6A (zeta 1) | CCT6A | chr7p11.2 |
| chaperonin containing TCP1, subunit 6A (zeta 1) | CCT6A | chr7p11.2 |
| signal transducer and activator of transcription 6, interleukin-4 induced | STAT6 | chr12q13 |
| signal transducer and activator of transcription 6, interleukin-4 induced | STAT6 | chr12q13 |
| phosphoinositide-3-kinase, regulatory subunit 2 (beta) /// interferon, gamma-inducible protein 30 | IFI30 /// PIK3R2 | chr19p13.1 /// chr19q13.2-q13.4 |
| collagen, type VI, alpha 3 | COL6A3 | chr2q37 |
| jun oncogene | JUN | chr1p32-p31 |
| jun oncogene | JUN | chr1p32-p31 |
| jun oncogene | JUN | chr1p32-p31 |
| SHC (Src homology 2 domain containing) transforming protein 1 | SHC1 | chr1q21 |
| nuclear factor of kappa light polypeptide gene enhancer in B-cells inhibitor, alpha | NFKBIA | chr14q13 |
| transforming growth factor, beta-induced, 68kDa | TGFBI | chr5q31 |
| Janus kinase 1 (a protein tyrosine kinase) | JAK1 | chr1p32.3-p31.3 |
| discs, large homolog 5 (Drosophila) | DLG5 | chr10q23 |
| CD14 molecule | CD14 | chr5q22-q32|5q31.1 |
| tumor susceptibility gene 101 | TSG101 | chr11p15 |
| v-rel reticuloendotheliosis viral oncogene homolog A, nuclear factor of kappa light polypeptide gene enhancer in B-cells 3, p65 (avian) | RELA | chr11q13 |
| polymerase (RNA) II (DNA directed) polypeptide B, 140kDa | POLR2B | chr4q12 |
| SWI/SNF related, matrix associated, actin dependent regulator of chromatin, subfamily d, member 2 | SMARCD2 | chr17q23-q24 |
| collagen, type III, alpha 1 (Ehlers-Danlos syndrome type IV, autosomal dominant) | COL3A1 | chr2q31 |
| serglycin | SRGN | chr10q22.1 |
| serglycin | SRGN | chr10q22.1 |
| nuclear receptor subfamily 3, group C, member 1 (glucocorticoid receptor) | NR3C1 | chr5q31.3 |
| nuclear receptor subfamily 3, group C, member 1 (glucocorticoid receptor) | NR3C1 | chr5q31.3 |
| solute carrier family 20 (phosphate transporter), member 1 | SLC20A1 | chr2q11-q14 |
| acetyl-Coenzyme A acyltransferase 2 | ACAA2 | chr18q21.1 |
| acetyl-Coenzyme A acyltransferase 2 | ACAA2 | chr18q21.1 |
| von Willebrand factor | VWF | chr12p13.3 |
| upstream transcription factor 2, c-fos interacting | USF2 | chr19q13 |
| CREB binding protein (Rubinstein-Taybi syndrome) | CREBBP | chr16p13.3 |
| TAF9 RNA polymerase II, TATA box binding protein (TBP)-associated factor, 32kDa | TAF9 | chr5q11.2-q13.1 |
| excision repair cross-complementing rodent repair deficiency, complementation group 3 (xeroderma pigmentosum group B complementing) | ERCC3 | chr2q21 |
| E1A binding protein p300 | EP300 | chr22q13.2 |
| solute carrier family 16, member 1 (monocarboxylic acid transporter 1) | SLC16A1 | chr1p12 |
| solute carrier family 16, member 1 (monocarboxylic acid transporter 1) | SLC16A1 | chr1p12 |
| solute carrier family 16, member 1 (monocarboxylic acid transporter 1) | SLC16A1 | chr1p12 |
| nicotinamide N-methyltransferase | NNMT | chr11q23.1 |
| nicotinamide N-methyltransferase | NNMT | chr11q23.1 |
| cyclin-dependent kinase inhibitor 1A (p21, Cip1) | CDKN1A | chr6p21.2 |
| fasciculation and elongation protein zeta 2 (zygin II) | FEZ2 | chr2p21 |
| polymerase (RNA) II (DNA directed) polypeptide G | POLR2G | chr11q13.1 |
| collagen, type I, alpha 1 | COL1A1 | chr17q21.33 |
| collagen, type I, alpha 1 | COL1A1 | chr17q21.33 |
| collagen, type I, alpha 1 | COL1A1 | chr17q21.33 |
| cytochrome c oxidase subunit Vb | COX5B | chr2cen-q13 |
| general transcription factor IIF, polypeptide 1, 74kDa | GTF2F1 | chr19p13.3 |
| general transcription factor IIF, polypeptide 1, 74kDa | GTF2F1 | chr19p13.3 |
| general transcription factor IIF, polypeptide 1, 74kDa | GTF2F1 | chr19p13.3 |
| MAX interactor 1 | MXI1 | chr10q24-q25 |
| acyl-Coenzyme A dehydrogenase, C-2 to C-3 short chain | ACADS | chr12q22-qter |
| cut-like homeobox 1 | CUX1 | chr7q22.1 |
| BCL2-associated athanogene | BAG1 | chr9p12 |
| collagen, type I, alpha 2 | COL1A2 | chr7q22.1 |
| collagen, type I, alpha 2 | COL1A2 | chr7q22.1 |
| mitogen-activated protein kinase kinase 2 | MAP2K2 | chr19p13.3 |
| protein phosphatase 3 (formerly 2B), catalytic subunit, alpha isoform | PPP3CA | chr4q21-q24 |
| protein phosphatase 3 (formerly 2B), catalytic subunit, alpha isoform | PPP3CA | chr4q21-q24 |
| protein phosphatase 3 (formerly 2B), catalytic subunit, beta isoform | PPP3CB | chr10q21-q22 |
| general transcription factor IIH, polypeptide 1, 62kDa | GTF2H1 | chr11p15.1-p14 |
| general transcription factor IIH, polypeptide 1, 62kDa | GTF2H1 | chr11p15.1-p14 |
| protein phosphatase 3 (formerly 2B), catalytic subunit, alpha isoform | PPP3CA | chr4q21-q24 |
| FXYD domain containing ion transport regulator 3 | FXYD3 | chr19q13.11-q13.12 |
| FXYD domain containing ion transport regulator 3 | FXYD3 | chr19q13.11-q13.12 |
| SMAD family member 4 | SMAD4 | chr18q21.1 |
| SMAD family member 4 | SMAD4 | chr18q21.1 |
| mitogen-activated protein kinase 14 | MAPK14 | chr6p21.3-p21.2 |
| heat shock 70kDa protein 1A /// heat shock 70kDa protein 1B | HSPA1A /// HSPA1B | chr6p21.3 |
| polymerase (RNA) II (DNA directed) polypeptide L, 7.6kDa | POLR2L | chr11p15 |
| nuclear receptor interacting protein 1 | NRIP1 | chr21q11.2 |
| nuclear receptor interacting protein 1 | NRIP1 | chr21q11.2 |
| glucuronidase, beta | GUSB | chr7q21.11 |
| mediator complex subunit 14 | MED14 | chrXp11.4-p11.2 |
| mediator complex subunit 14 | MED14 | chrXp11.4-p11.2 |
| mediator complex subunit 14 | MED14 | chrXp11.4-p11.2 |
| serpin peptidase inhibitor, clade E (nexin, plasminogen activator inhibitor type 1), member 1 | SERPINE1 | chr7q21.3-q22 |
| serpin peptidase inhibitor, clade E (nexin, plasminogen activator inhibitor type 1), member 1 | SERPINE1 | chr7q21.3-q22 |
| polymerase (RNA) II (DNA directed) polypeptide K, 7.0kDa | POLR2K | chr8q22.2 |
| polymerase (RNA) II (DNA directed) polypeptide K, 7.0kDa | POLR2K | chr8q22.2 |
| intercellular adhesion molecule 1 (CD54), human rhinovirus receptor | ICAM1 | chr19p13.3-p13.2 |
| intercellular adhesion molecule 1 (CD54), human rhinovirus receptor | ICAM1 | chr19p13.3-p13.2 |
| tumor necrosis factor, alpha-induced protein 3 | TNFAIP3 | chr6q23 |
| tumor necrosis factor, alpha-induced protein 3 | TNFAIP3 | chr6q23 |
| neuroblastoma RAS viral (v-ras) oncogene homolog | NRAS | chr1p13.2 |
| mitogen-activated protein kinase kinase 1 | MAP2K1 | chr15q22.1-q22.33 |
| general transcription factor IIA, 2, 12kDa | GTF2A2 | chr15q22.2 |
| general transcription factor IIE, polypeptide 2, beta 34kDa | GTF2E2 | chr8p21-p12 |
| insulin-like growth factor binding protein 2, 36kDa | IGFBP2 | chr2q33-q34 |
| polymerase (RNA) II (DNA directed) polypeptide A, 220kDa | POLR2A | chr17p13.1 |
| interferon gamma receptor 1 | IFNGR1 | chr6q23.3 |
| protein kinase, cAMP-dependent, catalytic, beta | PRKACB | chr1p36.1 |
| protein kinase, cAMP-dependent, catalytic, beta | PRKACB | chr1p36.1 |
| phosphoinositide-3-kinase, regulatory subunit 3 (gamma) | PIK3R3 | chr1p34.1 |
| protein kinase, cAMP-dependent, catalytic, alpha | PRKACA | chr19p13.1 |
| angiotensinogen (serpin peptidase inhibitor, clade A, member 8) | AGT | chr1q42-q43 |
| TAF15 RNA polymerase II, TATA box binding protein (TBP)-associated factor, 68kDa | TAF15 | chr17q11.1-q11.2 |
| phosphoenolpyruvate carboxykinase 2 (mitochondrial) | PCK2 | chr14q12 |
| interleukin 8 | IL8 | chr4q13-q21 |
| alanyl (membrane) aminopeptidase (aminopeptidase N, aminopeptidase M, microsomal aminopeptidase, CD13, p150) | ANPEP | chr15q25-q26 |
| four and a half LIM domains 2 | FHL2 | chr2q12-q14 |
| helicase-like transcription factor | HLTF | chr3q25.1-q26.1 |
| signal transducer and activator of transcription 5A | STAT5A | chr17q11.2 |
| secretory leukocyte peptidase inhibitor | SLPI | chr20q12 |
| SMAD family member 2 | SMAD2 | chr18q21.1 |
| SMAD family member 2 | SMAD2 | chr18q21.1 |
| SMAD family member 2 | SMAD2 | chr18q21.1 |
| MAD2L1 binding protein | MAD2L1BP | chr6p21.1 |
| TATA box binding protein | TBP | chr6q27 |
| SWI/SNF related, matrix associated, actin dependent regulator of chromatin, subfamily d, member 1 | SMARCD1 | chr12q13-q14 |
| estrogen-related receptor alpha | ESRRA | chr11q13 |
| mitogen-activated protein kinase 9 | MAPK9 | chr5q35 |
| platelet-activating factor acetylhydrolase, isoform Ib, gamma subunit 29kDa | PAFAH1B3 | chr19q13.1 |
| interleukin 4 receptor | IL4R | chr16p12.1-p11.2 |
| mitogen-activated protein kinase kinase 4 | MAP2K4 | chr17p11.2 |
| mitogen-activated protein kinase kinase 4 | MAP2K4 | chr17p11.2 |
| major histocompatibility complex, class II, DQ alpha 1 | HLA-DQA1 | chr6p21.3 |
| proteasome (prosome, macropain) assembly chaperone 1 | PSMG1 | chr21q22.3 |
| mediator complex subunit 1 | MED1 | chr17q12-q21.1 |
| mediator complex subunit 1 | MED1 | chr17q12-q21.1 |
| tumor necrosis factor receptor superfamily, member 1B | TNFRSF1B | chr1p36.3-p36.2 |
| syntaxin 4 | STX4 | chr16p11.2 |
| S100 calcium binding protein A9 | S100A9 | chr1q21 |
| menage a trois homolog 1, cyclin H assembly factor (Xenopus laevis) | MNAT1 | chr14q23 |
| chromosome 10 open reading frame 116 | C10orf116 | chr10q23.2 |
| TAF6 RNA polymerase II, TATA box binding protein (TBP)-associated factor, 80kDa | TAF6 | chr7q22.1 |
| general transcription factor IIH, polypeptide 4, 52kDa /// valyl-tRNA synthetase 2, mitochondrial (putative) | GTF2H4 /// VARS2 | chr6p21.3 |
| ELK1, member of ETS oncogene family | ELK1 | chrXp11.2 |
| CD163 molecule | CD163 | chr12p13.3 |
| phospholipase A2, group IIA (platelets, synovial fluid) | PLA2G2A | chr1p35 |
| polymerase (RNA) II (DNA directed) polypeptide D | POLR2D | chr2q21 |
| B-cell CLL/lymphoma 2 | BCL2 | chr18q21.33|18q21.3 |
| B-cell CLL/lymphoma 2 | BCL2 | chr18q21.33|18q21.3 |
| peptidase inhibitor 3, skin-derived (SKALP) | PI3 | chr20q12-q13 |
| interleukin 32 | IL32 | chr16p13.3 |
| p300/CBP-associated factor | PCAF | chr3p24 |
| paralemmin | PALM | chr19p13.3 |
| vascular cell adhesion molecule 1 | VCAM1 | chr1p32-p31 |
| phosphoinositide-3-kinase, catalytic, delta polypeptide | PIK3CD | chr1p36.2 |
| COX17 cytochrome c oxidase assembly homolog (S. cerevisiae) | COX17 | chr3q13.33 |
| TAF9 RNA polymerase II, TATA box binding protein (TBP)-associated factor, 32kDa | TAF9 | chr5q11.2-q13.1 |
| mitogen-activated protein kinase kinase kinase 7 interacting protein 1 | MAP3K7IP1 | chr22q13.1 |
| chemokine (C-X-C motif) ligand 9 | CXCL9 | chr4q21 |
| nuclear factor of kappa light polypeptide gene enhancer in B-cells inhibitor, epsilon | NFKBIE | chr6p21.1 |
| matrix metallopeptidase 9 (gelatinase B, 92kDa gelatinase, 92kDa type IV collagenase) | MMP9 | chr20q11.2-q13.1 |
| butyrophilin, subfamily 2, member A1 | BTN2A1 | chr6p22.1 |
| fatty acid binding protein 4, adipocyte | FABP4 | chr8q21 |
| v-Ki-ras2 Kirsten rat sarcoma viral oncogene homolog | KRAS | chr12p12.1 |
| v-Ki-ras2 Kirsten rat sarcoma viral oncogene homolog | KRAS | chr12p12.1 |
| CCAAT/enhancer binding protein (C/EBP), alpha | CEBPA | chr19q13.1 |
| cyclin H | CCNH | chr5q13.3-q14 |
| chemokine (C-C motif) ligand 4 | CCL4 | chr17q12 |
| interleukin 2 receptor, gamma (severe combined immunodeficiency) | IL2RG | chrXq13.1 |
| hydroxysteroid (11-beta) dehydrogenase 2 | HSD11B2 | chr16q22 |
| strawberry notch homolog 2 (Drosophila) | SBNO2 | chr19p13.3 |
| polymeric immunoglobulin receptor | PIGR | chr1q31-q41 |
| vitamin D (1,25- dihydroxyvitamin D3) receptor | VDR | chr12q13.11 |
| vitamin D (1,25- dihydroxyvitamin D3) receptor | VDR | chr12q13.11 |
| vitamin D (1,25- dihydroxyvitamin D3) receptor | VDR | chr12q13.11 |
| phosphoinositide-3-kinase, class 3 | PIK3C3 | chr18q12.3 |
| cAMP responsive element binding protein 1 | CREB1 | chr2q34 |
| cAMP responsive element binding protein 1 | CREB1 | chr2q34 |
| cAMP responsive element binding protein 1 | CREB1 | chr2q34 |
| S100 calcium binding protein P | S100P | chr4p16 |
| phosphoinositide-3-kinase, catalytic, alpha polypeptide | PIK3CA | chr3q26.3 |
| TNF receptor-associated factor 2 | TRAF2 | chr9q34 |
| chemokine (C-X-C motif) ligand 1 (melanoma growth stimulating activity, alpha) | CXCL1 | chr4q21 |
| matrix metallopeptidase 1 (interstitial collagenase) | MMP1 | chr11q22.3 |
| phosphoinositide-3-kinase, class 2, beta polypeptide | PIK3C2B | chr1q32 |
| protein phosphatase 3 (formerly 2B), regulatory subunit B, alpha isoform /// WD repeat domain 92 | PPP3R1 /// WDR92 | chr2p14 /// chr2p15 |
| protein phosphatase 3 (formerly 2B), regulatory subunit B, alpha isoform /// WD repeat domain 92 | PPP3R1 /// WDR92 | chr2p14 /// chr2p15 |
| UDP glucuronosyltransferase 1 family, polypeptide A10 /// UDP glucuronosyltransferase 1 family, polypeptide A8 /// UDP glucuronosyltransferase 1 family, polypeptide A6 /// UDP glucuronosyltransferase 1 family, polypeptide A9 /// UDP glucuronosyltransferase 1 family, polypeptide A4 /// UDP glucuronosyltransferase 1 family, polypeptide A1 | UGT1A1 /// UGT1A10 /// UGT1A4 /// UGT1A6 /// UGT1A8 /// UGT1A9 | chr2q37 |
| chemokine (C-X-C motif) ligand 10 | CXCL10 | chr4q21 |
| FK506 binding protein 5 | FKBP5 | chr6p21.3-p21.2 |
| matrix metallopeptidase 12 (macrophage elastase) | MMP12 | chr11q22.3 |
| 3-hydroxy-3-methylglutaryl-Coenzyme A synthase 2 (mitochondrial) | HMGCS2 | chr1p13-p12 |
| chemokine (C-C motif) ligand 5 | CCL5 | chr17q11.2-q12 |
| major histocompatibility complex, class II, DQ beta 1 /// major histocompatibility complex, class II, DQ beta 2 /// major histocompatibility complex, class II, DR beta 1 /// major histocompatibility complex, class II, DR beta 2 (pseudogene) /// major histocompatibility complex, class II, DR beta 3 /// major histocompatibility complex, class II, DR beta 4 /// major histocompatibility complex, class II, DR beta 5 /// ribonuclease, RNase A family, 2 (liver, eosinophil-derived neurotoxin) /// zinc finger protein 749 /// hypothetical protein LOC730415 /// similar to Major histocompatibility complex, class II, DR beta 4 /// similar to major histocompatibility complex, class II, DQ beta 1 /// similar to HLA class II histocompatibility antigen, DR-W53 beta chain /// similar to hCG1992647 | hCG_1998957 /// HLA-DQB1 /// HLA-DQB2 /// HLA-DRB1 /// HLA-DRB2 /// HLA-DRB3 /// HLA-DRB4 /// HLA-DRB5 /// LOC100133484 /// LOC100133583 /// LOC100133661 /// LOC100133811 /// LOC730415 /// RNASE2 /// ZNF749 | chr14q24-q31 /// chr19q13.43 /// chr6p21 /// chr6p21.3 |
| mucin 2, oligomeric mucus/gel-forming | MUC2 | chr11p15.5 |
| aldolase B, fructose-bisphosphate | ALDOB | chr9q21.3-q22.2 |
| aldolase B, fructose-bisphosphate | ALDOB | chr9q21.3-q22.2 |
| metallothionein 1G | MT1G | chr16q13 |
| mitogen-activated protein kinase 10 | MAPK10 | chr4q22.1-q23 |
| hydroxysteroid (17-beta) dehydrogenase 2 | HSD17B2 | chr16q24.1-q24.2 |
| mucin 4, cell surface associated | MUC4 | chr3q29 |
| prostaglandin E receptor 4 (subtype EP4) | PTGER4 | chr5p13.1 |
| prostaglandin E receptor 4 (subtype EP4) | PTGER4 | chr5p13.1 |
| B-cell CLL/lymphoma 3 | BCL3 | chr19q13.1-q13.2 |
| B-cell CLL/lymphoma 3 | BCL3 | chr19q13.1-q13.2 |
| toll-like receptor 2 | TLR2 | chr4q32 |
| protein tyrosine phosphatase, non-receptor type 2 | PTPN2 | chr18p11.3-p11.2 |
| cystatin A (stefin A) | CSTA | chr3q21 |
| trefoil factor 1 | TFF1 | chr21q22.3 |
| vasoactive intestinal peptide receptor 1 | VIPR1 | chr3p22 |
| signal transducer and activator of transcription 5B | STAT5B | chr17q11.2 |
| interleukin 1, beta | IL1B | chr2q14 |
| solute carrier family 22 (organic cation/carnitine transporter), member 5 | SLC22A5 | chr5q31 |
| solute carrier family 26 (sulfate transporter), member 2 | SLC26A2 | chr5q31-q34 |
| chemokine (C-C motif) ligand 3 /// chemokine (C-C motif) ligand 3-like 1 /// chemokine (C-C motif) ligand 3-like 3 /// similar to C-C motif chemokine 3-like 1 precursor (Small-inducible cytokine A3-like 1) (Tonsillar lymphocyte LD78 beta protein) (LD78-beta(1-70)) (G0/G1 switch regulatory protein 19-2) (G0S19-2 protein) (PAT 464.2) | CCL3 /// CCL3L1 /// CCL3L3 /// LOC728830 | chr17q11-q21 /// chr17q21.1 |
| mitogen-activated protein kinase kinase kinase 14 | MAP3K14 | chr17q21 |
| C-type lectin domain family 3, member B | CLEC3B | chr3p22-p21.3 |
| interleukin 6 (interferon, beta 2) | IL6 | chr7p21 |
| estrogen receptor 1 | ESR1 | chr6q25.1 |
| pre-B-cell leukemia homeobox 1 | PBX1 | chr1q23 |
| nuclear receptor subfamily 3, group C, member 2 | NR3C2 | chr4q31.1 |
| adducin 2 (beta) | ADD2 | chr2p14-p13 |
| complement factor D (adipsin) | CFD | chr19p13.3 |
| SMAD family member 3 | SMAD3 | chr15q22.33 |
| SMAD family member 3 | SMAD3 | chr15q22.33 |
| SMAD family member 3 | SMAD3 | chr15q22.33 |
| interleukin 1 receptor, type II | IL1R2 | chr2q12-q22 |
| acetyl-Coenzyme A acetyltransferase 1 (acetoacetyl Coenzyme A thiolase) | ACAT1 | chr11q22.3-q23.1 |
| prolactin | PRL | chr6p22.2-p21.3 |
| CD3e molecule, epsilon (CD3-TCR complex) | CD3E | chr11q23 |
| chemokine (C-C motif) ligand 20 | CCL20 | chr2q33-q37 |
| ISG15 ubiquitin-like modifier | ISG15 | chr1p36.33 |
| TNF receptor-associated factor 6 | TRAF6 | chr11p12 |
| FGFR1 oncogene partner | FGFR1OP | chr6q27 |
| FGFR1 oncogene partner | FGFR1OP | chr6q27 |
| macrophage stimulating 1 (hepatocyte growth factor-like) | MST1 | chr3p21 |
| interleukin 17 receptor A | IL17RA | chr22q11.1 |
| integrin, beta 7 | ITGB7 | chr12q13.13 |
| proopiomelanocortin (adrenocorticotropin/ beta-lipotropin/ alpha-melanocyte stimulating hormone/ beta-melanocyte stimulating hormone/ beta-endorphin) | POMC | chr2p23.3 |
| secretoglobin, family 1A, member 1 (uteroglobin) | SCGB1A1 | chr11q12.3-q13.1 |
| nuclear receptor coactivator 2 | NCOA2 | chr8q13.3 |
| nuclear receptor coactivator 2 | NCOA2 | chr8q13.3 |
| integrin, alpha M (complement component 3 receptor 3 subunit) | ITGAM | chr16p11.2 |
| integrin, alpha M (complement component 3 receptor 3 subunit) | ITGAM | chr16p11.2 |
| CD1d molecule | CD1D | chr1q22-q23 |
| regenerating islet-derived 3 alpha | REG3A | chr2p12 |
| matrix metallopeptidase 3 (stromelysin 1, progelatinase) | MMP3 | chr11q22.3 |
| Janus kinase 2 (a protein tyrosine kinase) | JAK2 | chr9p24 |
| Janus kinase 2 (a protein tyrosine kinase) | JAK2 | chr9p24 |
| regenerating islet-derived 1 beta (pancreatic stone protein, pancreatic thread protein) | REG1B | chr2p12 |
| fatty acid binding protein 1, liver | FABP1 | chr2p11 |
| nucleolar and coiled-body phosphoprotein 1 | NOLC1 | chr10q24.32 |
| solute carrier family 22 (organic cation/ergothioneine transporter), member 4 | SLC22A4 | chr5q31.1 |
| nuclear factor of activated T-cells, cytoplasmic, calcineurin-dependent 4 | NFATC4 | chr14q11.2 |
| major histocompatibility complex, class I, B /// major histocompatibility complex, class I, C /// MHC class I polypeptide-related sequence A /// MHC class I polypeptide-related sequence B | HLA-B /// HLA-C /// MICA /// MICB /// XXbac-BPG181B23.1 | chr6p21.3 /// chr6p21.33 |
| major histocompatibility complex, class I, B /// major histocompatibility complex, class I, C /// MHC class I polypeptide-related sequence A /// MHC class I polypeptide-related sequence B | HLA-B /// HLA-C /// MICA /// MICB /// XXbac-BPG181B23.1 | chr6p21.3 /// chr6p21.33 |
| general transcription factor IIE, polypeptide 1, alpha 56kDa | GTF2E1 | chr3q21-q24 |
| cytochrome P450, family 3, subfamily A, polypeptide 7 | CYP3A7 | chr7q21-q22.1 |
| TAF13 RNA polymerase II, TATA box binding protein (TBP)-associated factor, 18kDa | TAF13 | chr1p13.3 |
| interleukin 15 | IL15 | chr4q31 |
| meprin A, alpha (PABA peptide hydrolase) | MEP1A | chr6p12-p11 |
| mitogen-activated protein kinase 11 | MAPK11 | chr22q13.33 |
| zinc finger protein 91 | ZNF91 | chr19p13.1-p12 |
| protein tyrosine phosphatase, non-receptor type 22 (lymphoid) | PTPN22 | chr1p13.3-p13.1 |
| UDP glucuronosyltransferase 1 family, polypeptide A10 /// UDP glucuronosyltransferase 1 family, polypeptide A8 /// UDP glucuronosyltransferase 1 family, polypeptide A7 /// UDP glucuronosyltransferase 1 family, polypeptide A6 /// UDP glucuronosyltransferase 1 family, polypeptide A5 /// UDP glucuronosyltransferase 1 family, polypeptide A9 /// UDP glucuronosyltransferase 1 family, polypeptide A4 /// UDP glucuronosyltransferase 1 family, polypeptide A1 /// UDP glucuronosyltransferase 1 family, polypeptide A3 | UGT1A1 /// UGT1A10 /// UGT1A3 /// UGT1A4 /// UGT1A5 /// UGT1A6 /// UGT1A7 /// UGT1A8 /// UGT1A9 | chr2q37 |
| mitogen-activated protein kinase 12 | MAPK12 | chr22q13.33 |
| major histocompatibility complex, class II, DQ beta 1 /// major histocompatibility complex, class II, DQ beta 2 /// major histocompatibility complex, class II, DR beta 1 /// major histocompatibility complex, class II, DR beta 2 (pseudogene) /// major histocompatibility complex, class II, DR beta 3 /// major histocompatibility complex, class II, DR beta 4 /// major histocompatibility complex, class II, DR beta 5 /// ribonuclease, RNase A family, 2 (liver, eosinophil-derived neurotoxin) /// zinc finger protein 749 /// hypothetical protein LOC730415 /// similar to Major histocompatibility complex, class II, DR beta 4 /// similar to major histocompatibility complex, class II, DQ beta 1 /// similar to HLA class II histocompatibility antigen, DR-W53 beta chain /// similar to hCG1992647 | hCG_1998957 /// HLA-DQB1 /// HLA-DQB2 /// HLA-DRB1 /// HLA-DRB2 /// HLA-DRB3 /// HLA-DRB4 /// HLA-DRB5 /// LOC100133484 /// LOC100133583 /// LOC100133661 /// LOC100133811 /// LOC730415 /// RNASE2 /// ZNF749 | chr14q24-q31 /// chr19q13.43 /// chr6p21 /// chr6p21.3 |
| solute carrier family 26, member 3 | SLC26A3 | chr7q31 |
| adrenergic, beta-2-, receptor, surface | ADRB2 | chr5q31-q32 |
| growth hormone 2 | GH2 | chr17q24.2 |
| carbonic anhydrase IV | CA4 | chr17q23 |
| carbonic anhydrase IV | CA4 | chr17q23 |
| selectin E (endothelial adhesion molecule 1) | SELE | chr1q22-q25 |
| major histocompatibility complex, class I, B /// major histocompatibility complex, class I, C /// MHC class I polypeptide-related sequence A /// MHC class I polypeptide-related sequence B | HLA-B /// HLA-C /// MICA /// MICB /// XXbac-BPG181B23.1 | chr6p21.3 /// chr6p21.33 |
| toll-like receptor 3 | TLR3 | chr4q35 |
| hydroxy-delta-5-steroid dehydrogenase, 3 beta- and steroid delta-isomerase 2 | HSD3B2 | chr1p13.1 |
| interleukin 18 (interferon-gamma-inducing factor) | IL18 | chr11q22.2-q22.3 |
| major histocompatibility complex, class II, DO alpha | HLA-DOA | chr6p21.3 |
| interferon, gamma-inducible protein 16 | IFI16 | chr1q22 |
| phosphoinositide-3-kinase, catalytic, gamma polypeptide | PIK3CG | chr7q22.3 |
| phosphoinositide-3-kinase, catalytic, gamma polypeptide | PIK3CG | chr7q22.3 |
| chemokine (C-C motif) ligand 13 | CCL13 | chr17q11.2 |
| glucagon | GCG | chr2q36-q37 |
| zinc finger protein 365 | ZNF365 | chr10q21.2 |
| metallothionein 1H | MT1H | chr16q13 |
| tumor necrosis factor receptor superfamily, member 6b, decoy /// regulator of telomere elongation helicase 1 | RTEL1 /// TNFRSF6B | chr20q13.3 |
| cytochrome P450, family 4, subfamily F, polypeptide 3 /// cytochrome P450, family 4, subfamily F, polypeptide 2 | CYP4F2 /// CYP4F3 | chr19p13.2 /// chr19pter-p13.11 |
| cytochrome P450, family 4, subfamily F, polypeptide 3 | CYP4F3 | chr19p13.2 |
| general transcription factor IIA, 1, 19/37kDa | GTF2A1 | chr14q31.1 |
| muscle RAS oncogene homolog | MRAS | chr3q22.3 |
| SWI/SNF related, matrix associated, actin dependent regulator of chromatin, subfamily a, member 2 | SMARCA2 | chr9p22.3 |
| SWI/SNF related, matrix associated, actin dependent regulator of chromatin, subfamily a, member 2 | SMARCA2 | chr9p22.3 |
| SWI/SNF related, matrix associated, actin dependent regulator of chromatin, subfamily a, member 2 | SMARCA2 | chr9p22.3 |
| interleukin 24 | IL24 | chr1q32 |
| TATA box binding protein (TBP)-associated factor, RNA polymerase I, A, 48kDa | TAF1A | chr1q42 |
| BCL2-like 1 | BCL2L1 | chr20q11.21 |
| interleukin 7 | IL7 | chr8q12-q13 |
| POU class 2 homeobox 1 | POU2F1 | chr1q22-q23 |
| CD3g molecule, gamma (CD3-TCR complex) | CD3G | chr11q23 |
| adducin 2 (beta) | ADD2 | chr2p14-p13 |
| Mitogen-activated protein kinase kinase kinase 7 | MAP3K7 | chr6q16.1-q16.3 |
| mitogen-activated protein kinase kinase kinase 7 | MAP3K7 | chr6q16.1-q16.3 |
| glucokinase (hexokinase 4) regulator | GCKR | chr2p23 |
| interleukin 12 receptor, beta 1 | IL12RB1 | chr19p13.1 |
| tyrosine aminotransferase | TAT | chr16q22.1 |
| interleukin 11 | IL11 | chr19q13.3-q13.4 |
| interleukin 11 | IL11 | chr19q13.3-q13.4 |
| transforming growth factor, beta receptor I (activin A receptor type II-like kinase, 53kDa) | TGFBR1 | chr9q22 |
| bone gamma-carboxyglutamate (gla) protein (osteocalcin) /// polyamine-modulated factor 1 | BGLAP /// PMF1 | chr1q12 /// chr1q25-q31 |
| lymphotoxin alpha (TNF superfamily, member 1) | LTA | chr6p21.3 |
| chemokine (C-C motif) receptor 5 /// C-C chemokine receptor type 5-like | CCR5 /// LOC727797 | chr3p21.31 |
| interleukin 12 receptor, beta 2 | IL12RB2 | chr1p31.3-p31.2 |
| protein phosphatase 3 (formerly 2B), catalytic subunit, gamma isoform | PPP3CC | chr8p21.3 |
| TSC22 domain family, member 3 | TSC22D3 | chrXq22.3 |
| guanylate cyclase activator 2A (guanylin) | GUCA2A | chr1p35-p34 |
| B-cell CLL/lymphoma 2 | BCL2 | chr18q21.33|18q21.3 |
| B-cell CLL/lymphoma 2 | BCL2 | chr18q21.33|18q21.3 |
| interleukin 18 receptor accessory protein | IL18RAP | chr2q12 |
| phosphoinositide-3-kinase, regulatory subunit 2 (beta) | PIK3R2 | chr19q13.2-q13.4 |
| tumor necrosis factor (TNF superfamily, member 2) | TNF | chr6p21.3 |
| mitogen-activated protein kinase 6 | MAPK6 | chr15q21 |
| UDP glucuronosyltransferase 1 family, polypeptide A10 /// UDP glucuronosyltransferase 1 family, polypeptide A8 /// UDP glucuronosyltransferase 1 family, polypeptide A6 /// UDP glucuronosyltransferase 1 family, polypeptide A9 /// UDP glucuronosyltransferase 1 family, polypeptide A4 /// UDP glucuronosyltransferase 1 family, polypeptide A1 | UGT1A1 /// UGT1A10 /// UGT1A4 /// UGT1A6 /// UGT1A8 /// UGT1A9 | chr2q37 |
| interleukin 12A (natural killer cell stimulatory factor 1, cytotoxic lymphocyte maturation factor 1, p35) | IL12A | chr3q25.33-q26 |
| Janus kinase 3 (a protein tyrosine kinase, leukocyte) | JAK3 | chr19p13.1 |
| protein kinase, cAMP-dependent, catalytic, gamma | PRKACG | chr9q13 |
| mitogen-activated protein kinase 7 | MAPK7 | chr17p11.2 |
| transforming growth factor, beta receptor II (70/80kDa) | TGFBR2 | chr3p22 |
| UDP glucuronosyltransferase 2 family, polypeptide B15 | UGT2B15 | chr4q13 |
| nuclear factor of activated T-cells, cytoplasmic, calcineurin-dependent 3 | NFATC3 | chr16q22.2 |
| interleukin 10 | IL10 | chr1q31-q32 |
| chemokine (C-C motif) receptor 9 | CCR9 | chr3p21.3 |
| guanylate cyclase activator 2B (uroguanylin) | GUCA2B | chr1p34-p33 |
| defensin, alpha 5, Paneth cell-specific | DEFA5 | chr8pter-p21 |
| interleukin 4 | IL4 | chr5q31.1 |
| interleukin 4 | IL4 | chr5q31.1 |
| thromboxane A2 receptor | TBXA2R | chr19p13.3 |
| thromboxane A2 receptor | TBXA2R | chr19p13.3 |
| netrin 2-like (chicken) | NTN2L | chr16p13.3 |
| tumor necrosis factor receptor superfamily, member 1A | TNFRSF1A | chr12p13.2 |
| nuclear receptor coactivator 3 | NCOA3 | chr20q12 |
| nuclear receptor co-repressor 2 | NCOR2 | chr12q24 |
| ATPase, H+ transporting, lysosomal accessory protein 1 | ATP6AP1 | chrXq28 |
| defensin, alpha 6, Paneth cell-specific | DEFA6 | chr8pter-p21 |
| alcohol dehydrogenase 1A (class I), alpha polypeptide | ADH1A | chr4q21-q23 |
| interleukin 13 | IL13 | chr5q31 |
| mucin 1, cell surface associated | MUC1 | chr1q21 |
| interleukin 2 | IL2 | chr4q26-q27 |
| chemokine (C-X-C motif) ligand 3 | CXCL3 | chr4q21 |
| chemokine (C-X-C motif) ligand 5 | CXCL5 | chr4q12-q13 |
| glycophorin E | GYPE | chr4q31.1 |
| interleukin 12B (natural killer cell stimulatory factor 2, cytotoxic lymphocyte maturation factor 2, p40) | IL12B | chr5q31.1-q33.1 |
| interleukin 3 (colony-stimulating factor, multiple) | IL3 | chr5q31.1 |
| casein beta | CSN2 | chr4q21.1 |
| interleukin 5 (colony-stimulating factor, eosinophil) | IL5 | chr5q31.1 |
| calcium binding protein P22 | CHP | chr15q13.3 |
| nuclear factor of activated T-cells 5, tonicity-responsive | NFAT5 | chr16q22.1 |
| protein tyrosine phosphatase, non-receptor type 22 (lymphoid) | PTPN22 | chr1p13.3-p13.1 |
| protein tyrosine phosphatase, non-receptor type 22 (lymphoid) | PTPN22 | chr1p13.3-p13.1 |
| general transcription factor IIB | GTF2B | chr1p22-p21 |
| chorionic somatomammotropin hormone 1 (placental lactogen) /// chorionic somatomammotropin hormone 2 /// chorionic somatomammotropin hormone-like 1 /// growth hormone 1 /// growth hormone 2 | CSH1 /// CSH2 /// CSHL1 /// GH1 /// GH2 | chr17q24.2 |
| chorionic somatomammotropin hormone 1 (placental lactogen) /// chorionic somatomammotropin hormone 2 /// chorionic somatomammotropin hormone-like 1 /// growth hormone 1 /// growth hormone 2 | CSH1 /// CSH2 /// CSHL1 /// GH1 /// GH2 | chr17q24.2 |
| chemokine (C-C motif) ligand 7 | CCL7 | chr17q11.2-q12 |
| interleukin 9 | IL9 | chr5q31.1 |
| nuclear factor of activated T-cells, cytoplasmic, calcineurin-dependent 1 | NFATC1 | chr18q23 |
| amelogenin, Y-linked | AMELY | chrYp11.2 |
| progesterone receptor | PGR | chr11q22-q23 |
| Major histocompatibility complex, class II, DR beta 3 | HLA-DRB1 | chr6p21.3 |
| mitogen-activated protein kinase 1 | MAPK1 | chr22q11.2|22q11.21 |
| phosphoenolpyruvate carboxykinase 1 (soluble) | PCK1 | chr20q13.31 |
| interleukin 17A | IL17A | chr6p12 |
| related RAS viral (r-ras) oncogene homolog 2 | RRAS2 | chr11p15.2 |
| TAF4 RNA polymerase II, TATA box binding protein (TBP)-associated factor, 135kDa | TAF4 | chr20q13.33 |
| UDP glucuronosyltransferase 1 family, polypeptide A10 /// UDP glucuronosyltransferase 1 family, polypeptide A8 /// UDP glucuronosyltransferase 1 family, polypeptide A7 /// UDP glucuronosyltransferase 1 family, polypeptide A6 /// UDP glucuronosyltransferase 1 family, polypeptide A5 /// UDP glucuronosyltransferase 1 family, polypeptide A9 /// UDP glucuronosyltransferase 1 family, polypeptide A4 /// UDP glucuronosyltransferase 1 family, polypeptide A1 /// UDP glucuronosyltransferase 1 family, polypeptide A3 | UGT1A1 /// UGT1A10 /// UGT1A3 /// UGT1A4 /// UGT1A5 /// UGT1A6 /// UGT1A7 /// UGT1A8 /// UGT1A9 | chr2q37 |
| serum amyloid A1 /// serum amyloid A2 | SAA1 /// SAA2 | chr11p15.1 /// chr11p15.1-p14 |
| ras-related C3 botulinum toxin substrate 1 (rho family, small GTP binding protein Rac1) | RAC1 | chr7p22 |
| ras-related C3 botulinum toxin substrate 1 (rho family, small GTP binding protein Rac1) | RAC1 | chr7p22 |
| protein phosphatase 2 (formerly 2A), catalytic subunit, alpha isoform | PPP2CA | chr5q31.1 |
| heat shock 70kDa protein 8 | HSPA8 | chr11q24.1 |
| SMT3 suppressor of mif two 3 homolog 2 (S. cerevisiae) /// SMT3 suppressor of mif two 3 homolog 4 (S. cerevisiae) /// similar to SMT3 suppressor of mif two 3 homolog 2 pseudogene /// similar to small ubiquitin-related modifier 2 /// similar to SMT3B protein | LOC100127918 /// LOC100127922 /// LOC728825 /// SUMO2 /// SUMO4 | chr17q25.1 /// chr4q21.1 /// chr5q35.2 /// chr6q25 /// chrXq23 |
| SMT3 suppressor of mif two 3 homolog 2 (S. cerevisiae) | SUMO2 | chr17q25.1 |
| Ubiquitin-conjugating enzyme E2I (UBC9 homolog, yeast) | UBE2I | chr16p13.3 |
| SMT3 suppressor of mif two 3 homolog 1 (S. cerevisiae) | SUMO1 | chr2q33 |
| SMT3 suppressor of mif two 3 homolog 1 (S. cerevisiae) | SUMO1 | chr2q33 |
| TSC22 domain family, member 3 | TSC22D3 | chrXq22.3 |
| SWI/SNF related, matrix associated, actin dependent regulator of chromatin, subfamily a, member 4 | SMARCA4 | chr19p13.2 |
| SWI/SNF related, matrix associated, actin dependent regulator of chromatin, subfamily a, member 4 | SMARCA4 | chr19p13.2 |
| major histocompatibility complex, class I, C | HLA-C | chr6p21.3 |
| Heat shock 70kDa protein 4 | HSPA4 | chr5q31.1-q31.2 |
| heat shock 70kDa protein 4 | HSPA4 | chr5q31.1-q31.2 |
| nuclear receptor co-repressor 2 | NCOR2 | chr12q24 |
| nuclear receptor co-repressor 2 | NCOR2 | chr12q24 |
| major histocompatibility complex, class II, DR alpha | HLA-DRA | chr6p21.3 |
| sorcin | SRI | chr7q21.1 |
| sorcin | SRI | chr7q21.1 |
| transforming growth factor, beta receptor II (70/80kDa) | TGFBR2 | chr3p22 |
| interferon, gamma-inducible protein 16 | IFI16 | chr1q22 |
| interferon, gamma-inducible protein 16 | IFI16 | chr1q22 |
| signal transducer and activator of transcription 3 (acute-phase response factor) | STAT3 | chr17q21.31 |
| signal transducer and activator of transcription 3 (acute-phase response factor) | STAT3 | chr17q21.31 |
| polymerase (RNA) II (DNA directed) polypeptide C, 33kDa | POLR2C | chr16q13-q21 |
| esterase D/formylglutathione hydrolase | ESD | chr13q14.1-q14.2 |
| nuclear receptor coactivator 3 | NCOA3 | chr20q12 |
| nuclear receptor coactivator 3 | NCOA3 | chr20q12 |
| nuclear receptor coactivator 3 | NCOA3 | chr20q12 |
| nuclear receptor coactivator 1 | NCOA1 | chr2p23 |
| nuclear receptor coactivator 1 | NCOA1 | chr2p23 |
| nuclear receptor coactivator 1 | NCOA1 | chr2p23 |
| Immunoglobulin lambda joining 3 | IGL@ | chr22q11.1-q11.2 |
| major histocompatibility complex, class I, B /// major histocompatibility complex, class I, C /// MHC class I polypeptide-related sequence A /// MHC class I polypeptide-related sequence B | HLA-B /// HLA-C /// MICA /// MICB /// XXbac-BPG181B23.1 | chr6p21.3 /// chr6p21.33 |
| collagen, type VI, alpha 2 | COL6A2 | chr21q22.3 |
| v-fos FBJ murine osteosarcoma viral oncogene homolog | FOS | chr14q24.3 |
| Kruppel-like factor 5 (intestinal) | KLF5 | chr13q22.1 |
| Kruppel-like factor 5 (intestinal) | KLF5 | chr13q22.1 |
| nuclear factor of kappa light polypeptide gene enhancer in B-cells 1 (p105) | NFKB1 | chr4q24 |
| ATPase, Ca++ transporting, plasma membrane 1 | ATP2B1 | chr12q21.3 |
| carbonic anhydrase II | CA2 | chr8q22 |
| polymerase (RNA) II (DNA directed) polypeptide H | POLR2H | chr3q28 |
| major histocompatibility complex, class II, DQ beta 1 /// major histocompatibility complex, class II, DQ beta 2 /// major histocompatibility complex, class II, DR beta 1 /// major histocompatibility complex, class II, DR beta 2 (pseudogene) /// major histocompatibility complex, class II, DR beta 3 /// major histocompatibility complex, class II, DR beta 4 /// major histocompatibility complex, class II, DR beta 5 /// ribonuclease, RNase A family, 2 (liver, eosinophil-derived neurotoxin) /// zinc finger protein 749 /// hypothetical protein LOC730415 /// similar to Major histocompatibility complex, class II, DR beta 4 /// similar to major histocompatibility complex, class II, DQ beta 1 /// similar to HLA class II histocompatibility antigen, DR-W53 beta chain /// similar to hCG1992647 | hCG_1998957 /// HLA-DQB1 /// HLA-DQB2 /// HLA-DRB1 /// HLA-DRB2 /// HLA-DRB3 /// HLA-DRB4 /// HLA-DRB5 /// LOC100133484 /// LOC100133583 /// LOC100133661 /// LOC100133811 /// LOC730415 /// RNASE2 /// ZNF749 | chr14q24-q31 /// chr19q13.43 /// chr6p21 /// chr6p21.3 |
| TAF11 RNA polymerase II, TATA box binding protein (TBP)-associated factor, 28kDa | TAF11 | chr6p21.31 |
| TAF12 RNA polymerase II, TATA box binding protein (TBP)-associated factor, 20kDa | TAF12 | chr1p35.3 |
| major histocompatibility complex, class II, DQ beta 1 | HLA-DQB1 | chr6p21.3 |
| polymerase (RNA) II (DNA directed) polypeptide F | POLR2F | chr22q13.1 |
| SWI/SNF related, matrix associated, actin dependent regulator of chromatin, subfamily d, member 1 | SMARCD1 | chr12q13-q14 |
| TAF2 RNA polymerase II, TATA box binding protein (TBP)-associated factor, 150kDa | TAF2 | chr8q24.12 |
| general transcription factor IIF, polypeptide 2, 30kDa | GTF2F2 | chr13q14 |
| thiosulfate sulfurtransferase (rhodanese) | TST | chr22q13.1 |
| sulfotransferase family, cytosolic, 1A, phenol-preferring, member 3 /// GIY-YIG domain containing 2 /// sulfotransferase family, cytosolic, 1A, phenol-preferring, member 4 /// GIY-YIG domain containing 1 | GIYD1 /// GIYD2 /// SULT1A3 /// SULT1A4 | chr16p11.2 |
| CD74 molecule, major histocompatibility complex, class II invariant chain | CD74 | chr5q32 |
| nuclear factor of activated T-cells, cytoplasmic, calcineurin-dependent 1 | NFATC1 | chr18q23 |
| T cell receptor alpha locus /// T cell receptor alpha constant | TRA@ /// TRAC | chr14q11 /// chr14q11.2 |
| 6-pyruvoyltetrahydropterin synthase | PTS | chr11q22.3-q23.3 |
| regenerating islet-derived 1 alpha (pancreatic stone protein, pancreatic thread protein) | REG1A | chr2p12 |
| dodecenoyl-Coenzyme A delta isomerase (3,2 trans-enoyl-Coenzyme A isomerase) | DCI | chr16p13.3 |
| chemokine (C-X-C motif) ligand 2 | CXCL2 | chr4q21 |
| protein phosphatase 3 (formerly 2B), catalytic subunit, beta isoform | PPP3CB | chr10q21-q22 |
| interleukin 33 | IL33 | chr9p24.1 |
| major histocompatibility complex, class II, DQ beta 1 | HLA-DQB1 | chr6p21.3 |
| interleukin 16 (lymphocyte chemoattractant factor) | IL16 | chr15q26.3 |
| interleukin 16 (lymphocyte chemoattractant factor) | IL16 | chr15q26.3 |
| CASP2 and RIPK1 domain containing adaptor with death domain | CRADD | chr12q21.33-q23.1 |
| v-rel reticuloendotheliosis viral oncogene homolog A, nuclear factor of kappa light polypeptide gene enhancer in B-cells 3, p65 (avian) | RELA | chr11q13 |
| solute carrier family 16, member 1 (monocarboxylic acid transporter 1) | SLC16A1 | chr1p12 |
| transforming growth factor, beta 2 | TGFB2 | chr1q41 |
| transforming growth factor, beta 2 | TGFB2 | chr1q41 |
| mitogen-activated protein kinase kinase 7 | MAP2K7 | chr19p13.3-p13.2 |
| mitogen-activated protein kinase kinase 7 | MAP2K7 | chr19p13.3-p13.2 |
| natriuretic peptide precursor A | NPPA | chr1p36.21 |
| signal transducer and activator of transcription 1, 91kDa | STAT1 | chr2q32.2 |
| ATP-binding cassette, sub-family B (MDR/TAP), member 1 | ABCB1 | chr7q21.1 |
| ATP-binding cassette, sub-family B (MDR/TAP), member 1 /// ATP-binding cassette, sub-family B (MDR/TAP), member 4 | ABCB1 /// ABCB4 | chr7q21.1 |
| CD247 molecule | CD247 | chr1q22-q23 |
| nitric oxide synthase 2A (inducible, hepatocytes) | NOS2A | chr17q11.2-q12 |
| TAF5 RNA polymerase II, TATA box binding protein (TBP)-associated factor, 100kDa | TAF5 | chr10q24-q25.2 |
| mitogen-activated protein kinase 13 | MAPK13 | chr6p21.31 |
| mitogen-activated protein kinase 13 | MAPK13 | chr6p21.31 |
| leukotriene B4 receptor | LTB4R | chr14q11.2-q12 |
| chemokine (C-C motif) ligand 11 | CCL11 | chr17q21.1-q21.2 |
| peripheral myelin protein 22 | PMP22 | chr17p12-p11.2 |
| nuclear factor of activated T-cells, cytoplasmic, calcineurin-dependent 1 | NFATC1 | chr18q23 |
| toll-like receptor 5 | TLR5 | chr1q41-q42 |
| toll-like receptor 1 | TLR1 | chr4p14 |
| heat shock 70kDa protein 1-like | HSPA1L | chr6p21.3 |
| heat shock protein 90kDa alpha (cytosolic), class A member 1 | HSP90AA1 | chr14q32.33 |
| colony stimulating factor 2 (granulocyte-macrophage) | CSF2 | chr5q31.1 |
| colony stimulating factor 2 (granulocyte-macrophage) | CSF2 | chr5q31.1 |
| nuclear receptor coactivator 1 | NCOA1 | chr2p23 |
| heat shock 70kDa protein 8 | HSPA8 | chr11q24.1 |
| interferon, gamma | IFNG | chr12q14 |
| ELK1, member of ETS oncogene family | ELK1 | chrXp11.2 |
| fatty acid binding protein 6, ileal (gastrotropin) | FABP6 | chr5q33.3-q34 |
| mitogen-activated protein kinase 14 | MAPK14 | chr6p21.3-p21.2 |
| discs, large homolog 5 (Drosophila) | DLG5 | chr10q23 |
| metallothionein 1G | MT1G | chr16q13 |
| mitogen-activated protein kinase 8 | MAPK8 | chr10q11.22 |
| fibronectin 1 | FN1 | chr2q34 |
| nuclear factor of activated T-cells, cytoplasmic, calcineurin-dependent 3 | NFATC3 | chr16q22.2 |
| nuclear factor of activated T-cells, cytoplasmic, calcineurin-dependent 3 | NFATC3 | chr16q22.2 |
| mitogen-activated protein kinase 9 | MAPK9 | chr5q35 |
| sulfotransferase family, cytosolic, 1A, phenol-preferring, member 3 /// GIY-YIG domain containing 2 /// sulfotransferase family, cytosolic, 1A, phenol-preferring, member 4 /// GIY-YIG domain containing 1 | GIYD1 /// GIYD2 /// SULT1A3 /// SULT1A4 | chr16p11.2 |
| mitogen-activated protein kinase 8 | MAPK8 | chr10q11.22 |
| fasciculation and elongation protein zeta 2 (zygin II) | FEZ2 | chr2p21 |
| major histocompatibility complex, class II, DQ beta 1 | HLA-DQB1 | chr6p21.3 |
| periostin, osteoblast specific factor | POSTN | chr13q13.3 |
| ELK1, member of ETS oncogene family | ELK1 | chrXp11.2 |
| T cell receptor alpha locus /// T cell receptor alpha variable 20 /// T cell receptor alpha joining 17 /// T cell receptor alpha constant | TRA@ /// TRAC /// TRAJ17 /// TRAV20 | chr14q11 /// chr14q11.2 |
| major histocompatibility complex, class II, DR alpha | HLA-DRA | chr6p21.3 |
| ubiquitin-conjugating enzyme E2I (UBC9 homolog, yeast) | UBE2I | chr16p13.3 |
| heat shock 70kDa protein 4 | HSPA4 | chr5q31.1-q31.2 |
| heat shock 70kDa protein 4 | HSPA4 | chr5q31.1-q31.2 |
| cytochrome c oxidase subunit Vb | COX5B | chr2cen-q13 |
| SMT3 suppressor of mif two 3 homolog 1 (S. cerevisiae) | SUMO1 | chr2q33 |
| mitogen-activated protein kinase 14 | MAPK14 | chr6p21.3-p21.2 |
| nuclear factor of activated T-cells, cytoplasmic, calcineurin-dependent 1 | NFATC1 | chr18q23 |
| Janus kinase 3 (a protein tyrosine kinase, leukocyte) | JAK3 | chr19p13.1 |
| Janus kinase 3 (a protein tyrosine kinase, leukocyte) | JAK3 | chr19p13.1 |
| androgen receptor (dihydrotestosterone receptor; testicular feminization; spinal and bulbar muscular atrophy; Kennedy disease) | AR | chrXq11.2-q12 |
| major histocompatibility complex, class II, DO alpha | HLA-DOA | chr6p21.3 |
| collagen, type III, alpha 1 (Ehlers-Danlos syndrome type IV, autosomal dominant) | COL3A1 | chr2q31 |
| inducible T-cell co-stimulator ligand | ICOSLG | chr21q22.3 |
| inducible T-cell co-stimulator ligand | ICOSLG | chr21q22.3 |
| inducible T-cell co-stimulator ligand | ICOSLG | chr21q22.3 |
| phosphoinositide-3-kinase, catalytic, delta polypeptide | PIK3CD | chr1p36.2 |
| estrogen receptor 1 | ESR1 | chr6q25.1 |
| estrogen receptor 1 | ESR1 | chr6q25.1 |
| estrogen receptor 1 | ESR1 | chr6q25.1 |
| butyrophilin, subfamily 2, member A1 | BTN2A1 | chr6p22.1 |
| cyclin-dependent kinase 7 | CDK7 | chr5q12.1 |
| nuclear receptor coactivator 3 | NCOA3 | chr20q12 |
| aldolase B, fructose-bisphosphate | ALDOB | chr9q21.3-q22.2 |
| interleukin 1 receptor, type II | IL1R2 | chr2q12-q22 |
| BCL2-associated athanogene | BAG1 | chr9p12 |
| mitogen-activated protein kinase 11 | MAPK11 | chr22q13.33 |
| mitogen-activated protein kinase 11 | MAPK11 | chr22q13.33 |
| interleukin 8 | IL8 | chr4q13-q21 |
| growth hormone 2 | GH2 | chr17q24.2 |
| tumor necrosis factor receptor superfamily, member 6b, decoy /// regulator of telomere elongation helicase 1 | RTEL1 /// TNFRSF6B | chr20q13.3 |
| mitogen-activated protein kinase kinase kinase 7 | MAP3K7 | chr6q16.1-q16.3 |
| mitogen-activated protein kinase kinase kinase 7 | MAP3K7 | chr6q16.1-q16.3 |
| heat shock 70kDa protein 2 | HSPA2 | chr14q24.1 |
| mitogen-activated protein kinase 14 | MAPK14 | chr6p21.3-p21.2 |
| phosphoinositide-3-kinase, regulatory subunit 3 (gamma) | PIK3R3 | chr1p34.1 |
| thromboxane A2 receptor | TBXA2R | chr19p13.3 |
| androgen receptor (dihydrotestosterone receptor; testicular feminization; spinal and bulbar muscular atrophy; Kennedy disease) | AR | chrXq11.2-q12 |
| estrogen receptor 1 | ESR1 | chr6q25.1 |
| immunoglobulin heavy locus /// immunoglobulin heavy constant alpha 1 /// immunoglobulin heavy constant alpha 2 (A2m marker) /// immunoglobulin heavy constant gamma 1 (G1m marker) /// immunoglobulin heavy constant gamma 3 (G3m marker) /// immunoglobulin heavy constant mu /// immunoglobulin heavy variable 7-81 /// immunoglobulin heavy variable 4-31 /// similar to hCG1812074 /// similar to hCG2038920 | IGH@ /// IGHA1 /// IGHA2 /// IGHG1 /// IGHG3 /// IGHM /// IGHV4-31 /// IGHV7-81 /// LOC100133739 /// LOC642131 | chr14q32.33 /// chr15q11.2 |
| immunoglobulin heavy locus /// immunoglobulin heavy constant alpha 1 /// immunoglobulin heavy constant alpha 2 (A2m marker) /// immunoglobulin heavy constant delta /// immunoglobulin heavy constant gamma 1 (G1m marker) /// immunoglobulin heavy constant gamma 3 (G3m marker) /// immunoglobulin heavy constant gamma 4 (G4m marker) /// immunoglobulin heavy constant mu /// immunoglobulin heavy variable 4-31 /// similar to hCG1812074 /// similar to Ig heavy chain V-II region ARH-77 precursor /// hypothetical LOC100126583 | IGH@ /// IGHA1 /// IGHA2 /// IGHD /// IGHG1 /// IGHG3 /// IGHG4 /// IGHM /// IGHV4-31 /// LOC100126583 /// LOC100134331 /// LOC642131 /// LOC652128 | chr14q32.33 /// chr15q11.2 |
| immunoglobulin heavy locus /// immunoglobulin heavy constant alpha 1 /// immunoglobulin heavy constant alpha 2 (A2m marker) /// immunoglobulin heavy constant delta /// immunoglobulin heavy constant gamma 1 (G1m marker) /// immunoglobulin heavy constant gamma 3 (G3m marker) /// immunoglobulin heavy constant gamma 4 (G4m marker) /// immunoglobulin heavy constant mu /// immunoglobulin heavy variable 4-31 /// similar to hCG1812074 /// similar to Ig heavy chain V-II region ARH-77 precursor /// hypothetical LOC100126583 | IGH@ /// IGHA1 /// IGHA2 /// IGHD /// IGHG1 /// IGHG3 /// IGHG4 /// IGHM /// IGHV4-31 /// LOC100126583 /// LOC100134331 /// LOC642131 /// LOC652128 | chr14q32.33 /// chr15q11.2 |
| immunoglobulin heavy locus /// immunoglobulin heavy constant alpha 1 /// immunoglobulin heavy constant alpha 2 (A2m marker) /// immunoglobulin heavy constant delta /// immunoglobulin heavy constant gamma 1 (G1m marker) /// immunoglobulin heavy constant gamma 3 (G3m marker) /// immunoglobulin heavy constant gamma 4 (G4m marker) /// immunoglobulin heavy constant mu /// immunoglobulin heavy variable 4-31 /// similar to hCG1812074 /// similar to Ig heavy chain V-II region ARH-77 precursor /// hypothetical LOC100126583 /// similar to hCG2038920 | IGH@ /// IGHA1 /// IGHA2 /// IGHD /// IGHG1 /// IGHG3 /// IGHG4 /// IGHM /// IGHV4-31 /// LOC100126583 /// LOC100133739 /// LOC100134331 /// LOC642131 /// LOC652128 | chr14q32.33 /// chr15q11.2 |
| immunoglobulin heavy locus /// immunoglobulin heavy constant alpha 1 /// immunoglobulin heavy constant alpha 2 (A2m marker) /// immunoglobulin heavy constant gamma 1 (G1m marker) /// immunoglobulin heavy constant gamma 3 (G3m marker) /// immunoglobulin heavy constant mu /// immunoglobulin heavy variable group /// immunoglobulin heavy variable 4-31 /// similar to hCG2038920 | IGH@ /// IGHA1 /// IGHA2 /// IGHG1 /// IGHG3 /// IGHM /// IGHV4-31 /// IGHV@ /// LOC100133739 | chr14q32.33 |
| immunoglobulin heavy locus /// immunoglobulin heavy constant alpha 1 /// immunoglobulin heavy constant gamma 1 (G1m marker) /// immunoglobulin heavy constant gamma 2 (G2m marker) /// immunoglobulin heavy constant gamma 3 (G3m marker) /// immunoglobulin heavy constant mu /// similar to Ig heavy chain V-III region VH26 precursor /// hypothetical LOC100126583 /// similar to hCG2038920 | IGH@ /// IGHA1 /// IGHG1 /// IGHG2 /// IGHG3 /// IGHM /// LOC100126583 /// LOC100133739 /// LOC652494 | chr14q32.33 |
| immunoglobulin heavy constant alpha 1 /// immunoglobulin heavy constant delta /// immunoglobulin heavy constant gamma 1 (G1m marker) /// immunoglobulin heavy constant gamma 3 (G3m marker) /// immunoglobulin heavy constant mu /// immunoglobulin heavy variable group /// immunoglobulin heavy variable 4-31 /// hypothetical LOC100126583 | IGHA1 /// IGHD /// IGHG1 /// IGHG3 /// IGHM /// IGHV4-31 /// IGHV@ /// LOC100126583 | chr14q32.33 |
| major histocompatibility complex, class II, DQ beta 1 | HLA-DQB1 | chr6p21.3 |
| Immunoglobulin lambda joining 3 | IGL@ | chr22q11.1-q11.2 |
| major histocompatibility complex, class II, DQ beta 1 | HLA-DQB1 | chr6p21.3 |
| POU class 2 homeobox 2 | POU2F2 | chr19q13.2 |
| son of sevenless homolog 2 (Drosophila) | SOS2 | chr14q21 |
| nuclear receptor subfamily 3, group C, member 1 (glucocorticoid receptor) | NR3C1 | chr5q31.3 |
| actin related protein 2/3 complex, subunit 4, 20kDa /// tubulin tyrosine ligase-like family, member 3 | ARPC4 /// TTLL3 | chr3p25.3 |
| interferon gamma receptor 1 | IFNGR1 | chr6q23.3 |
| mucin 1, cell surface associated | MUC1 | chr1q21 |
| fibronectin 1 | FN1 | chr2q34 |
| polymerase (RNA) II (DNA directed) polypeptide L, 7.6kDa | POLR2L | chr11p15 |
| POU class 2 homeobox 2 | POU2F2 | chr19q13.2 |
| major histocompatibility complex, class I, C | HLA-C | chr6p21.3 |
| CREB binding protein (Rubinstein-Taybi syndrome) | CREBBP | chr16p13.3 |
| immunoglobulin heavy locus /// immunoglobulin heavy constant alpha 1 /// immunoglobulin heavy constant alpha 2 (A2m marker) /// immunoglobulin heavy constant delta /// immunoglobulin heavy constant gamma 1 (G1m marker) /// immunoglobulin heavy constant gamma 3 (G3m marker) /// immunoglobulin heavy constant gamma 4 (G4m marker) /// immunoglobulin heavy constant mu /// immunoglobulin heavy variable 4-31 /// similar to hCG1812074 /// similar to Ig heavy chain V-II region ARH-77 precursor /// hypothetical LOC100126583 | IGH@ /// IGHA1 /// IGHA2 /// IGHD /// IGHG1 /// IGHG3 /// IGHG4 /// IGHM /// IGHV4-31 /// LOC100126583 /// LOC100134331 /// LOC642131 /// LOC652128 | chr14q32.33 /// chr15q11.2 |
| cytochrome P450, family 3, subfamily A, polypeptide 7 | CYP3A7 | chr7q21-q22.1 |
| GNAS complex locus | GNAS | chr20q13.3 |
| immunoglobulin heavy constant alpha 1 /// immunoglobulin heavy constant gamma 1 (G1m marker) /// immunoglobulin heavy constant gamma 3 (G3m marker) /// immunoglobulin heavy constant mu /// immunoglobulin heavy variable group /// immunoglobulin heavy variable 4-31 /// similar to hCG2038920 | IGHA1 /// IGHG1 /// IGHG3 /// IGHM /// IGHV4-31 /// IGHV@ /// LOC100133739 | chr14q32.33 |
| T cell receptor alpha locus | TRA@ | chr14q11.2 |
| major histocompatibility complex, class I, B /// major histocompatibility complex, class I, C /// MHC class I polypeptide-related sequence A /// MHC class I polypeptide-related sequence B /// family with sequence similarity 20, member B | FAM20B /// HLA-B /// HLA-C /// MICA /// MICB /// XXbac-BPG181B23.1 | chr1q25 /// chr6p21.3 /// chr6p21.33 |
| heat shock 70kDa protein 5 (glucose-regulated protein, 78kDa) | HSPA5 | chr9q33-q34.1 |
| nucleolar and coiled-body phosphoprotein 1 | NOLC1 | chr10q24.32 |
| nucleolar and coiled-body phosphoprotein 1 | NOLC1 | chr10q24.32 |
| collagen, type IV, alpha 2 | COL4A2 | chr13q34 |
| collagen, type IV, alpha 2 | COL4A2 | chr13q34 |
| heat shock protein 90kDa alpha (cytosolic), class A member 1 | HSP90AA1 | chr14q32.33 |
| heat shock protein 90kDa alpha (cytosolic), class A member 1 | HSP90AA1 | chr14q32.33 |
| mitogen-activated protein kinase 3 | MAPK3 | chr16p11.2 |
| Hypothetical LOC100131938 | PBX1 | chr1q23 |
| pre-B-cell leukemia homeobox 1 | PBX1 | chr1q23 |
| phosphoinositide-3-kinase, regulatory subunit 1 (alpha) | PIK3R1 | chr5q13.1 |
| phosphoinositide-3-kinase, regulatory subunit 1 (alpha) | PIK3R1 | chr5q13.1 |
| phosphoinositide-3-kinase, regulatory subunit 1 (alpha) | PIK3R1 | chr5q13.1 |
| SWI/SNF related, matrix associated, actin dependent regulator of chromatin, subfamily a, member 2 | SMARCA2 | chr9p22.3 |
| SWI/SNF related, matrix associated, actin dependent regulator of chromatin, subfamily a, member 2 | SMARCA2 | chr9p22.3 |
| mitogen-activated protein kinase 1 | MAPK1 | chr22q11.2|22q11.21 |
| GNAS complex locus | GNAS | chr20q13.3 |
| BCL2-like 1 | BCL2L1 | chr20q11.21 |
| SREBF chaperone | SCAP | chr3p21.31 |
| fibronectin 1 | FN1 | chr2q34 |
| CCAAT/enhancer binding protein (C/EBP), beta | CEBPB | chr20q13.1 |
| SWI/SNF related, matrix associated, actin dependent regulator of chromatin, subfamily a, member 4 | SMARCA4 | chr19p13.2 |
| lipocalin 2 | LCN2 | chr9q34 |
| signal transducer and activator of transcription 5B | STAT5B | chr17q11.2 |
| signal transducer and activator of transcription 5B | STAT5B | chr17q11.2 |
| related RAS viral (r-ras) oncogene homolog 2 | RRAS2 | chr11p15.2 |
| related RAS viral (r-ras) oncogene homolog 2 | RRAS2 | chr11p15.2 |
| related RAS viral (r-ras) oncogene homolog | RRAS | chr19q13.3-qter |
| interleukin 1 receptor antagonist | IL1RN | chr2q14.2 |
| interleukin 1 receptor antagonist | IL1RN | chr2q14.2 |
| secreted protein, acidic, cysteine-rich (osteonectin) | SPARC | chr5q31.3-q32 |
| major histocompatibility complex, class II, DQ alpha 1 /// major histocompatibility complex, class II, DQ alpha 2 | HLA-DQA1 /// HLA-DQA2 | chr6p21.3 |
| phosphoinositide-3-kinase, catalytic, beta polypeptide | PIK3CB | chr3q22.3 |
| son of sevenless homolog 1 (Drosophila) | SOS1 | chr2p22-p21 |
| son of sevenless homolog 1 (Drosophila) | SOS1 | chr2p22-p21 |
| polymerase (RNA) II (DNA directed) polypeptide J, 13.3kDa | POLR2J | chr7q22.1 |
| prune homolog 2 (Drosophila) /// similar to KIAA0367 | LOC100129762 /// PRUNE2 | chr9q21.13 |
| prune homolog 2 (Drosophila) /// similar to KIAA0367 | LOC100129762 /// PRUNE2 | chr9q21.13 |
| son of sevenless homolog 2 (Drosophila) | SOS2 | chr14q21 |
| ATPase, Ca++ transporting, plasma membrane 1 | ATP2B1 | chr12q21.3 |
| polymerase (RNA) II (DNA directed) polypeptide I, 14.5kDa | POLR2I | chr19q12 |
| v-Ha-ras Harvey rat sarcoma viral oncogene homolog | HRAS | chr11p15.5 |
| major histocompatibility complex, class II, DQ beta 1 /// major histocompatibility complex, class II, DQ beta 2 /// major histocompatibility complex, class II, DR beta 1 /// major histocompatibility complex, class II, DR beta 2 (pseudogene) /// major histocompatibility complex, class II, DR beta 3 /// major histocompatibility complex, class II, DR beta 4 /// major histocompatibility complex, class II, DR beta 5 /// ribonuclease, RNase A family, 2 (liver, eosinophil-derived neurotoxin) /// zinc finger protein 749 /// hypothetical protein LOC730415 /// similar to Major histocompatibility complex, class II, DR beta 4 /// similar to major histocompatibility complex, class II, DQ beta 1 /// similar to HLA class II histocompatibility antigen, DR-W53 beta chain /// similar to hCG1992647 | hCG_1998957 /// HLA-DQB1 /// HLA-DQB2 /// HLA-DRB1 /// HLA-DRB2 /// HLA-DRB3 /// HLA-DRB4 /// HLA-DRB5 /// LOC100133484 /// LOC100133583 /// LOC100133661 /// LOC100133811 /// LOC730415 /// RNASE2 /// ZNF749 | chr14q24-q31 /// chr19q13.43 /// chr6p21 /// chr6p21.3 |
| major histocompatibility complex, class II, DQ beta 1 /// major histocompatibility complex, class II, DQ beta 2 /// major histocompatibility complex, class II, DR beta 1 /// major histocompatibility complex, class II, DR beta 2 (pseudogene) /// major histocompatibility complex, class II, DR beta 3 /// major histocompatibility complex, class II, DR beta 4 /// major histocompatibility complex, class II, DR beta 5 /// ribonuclease, RNase A family, 2 (liver, eosinophil-derived neurotoxin) /// zinc finger protein 749 /// hypothetical protein LOC730415 /// similar to Major histocompatibility complex, class II, DR beta 4 /// similar to major histocompatibility complex, class II, DQ beta 1 /// similar to HLA class II histocompatibility antigen, DR-W53 beta chain /// similar to hCG1992647 | hCG_1998957 /// HLA-DQB1 /// HLA-DQB2 /// HLA-DRB1 /// HLA-DRB2 /// HLA-DRB3 /// HLA-DRB4 /// HLA-DRB5 /// LOC100133484 /// LOC100133583 /// LOC100133661 /// LOC100133811 /// LOC730415 /// RNASE2 /// ZNF749 | chr14q24-q31 /// chr19q13.43 /// chr6p21 /// chr6p21.3 |
| phosphoinositide-3-kinase, class 2, alpha polypeptide | PIK3C2A | chr11p15.5-p14 |
| TAF4 RNA polymerase II, TATA box binding protein (TBP)-associated factor, 135kDa | TAF4 | chr20q13.33 |
| protein tyrosine phosphatase, non-receptor type 2 | PTPN2 | chr18p11.3-p11.2 |
| protein tyrosine phosphatase, non-receptor type 2 | PTPN2 | chr18p11.3-p11.2 |
| cyclin-dependent kinase inhibitor 1C (p57, Kip2) | CDKN1C | chr11p15.5 |
| Cyclin-dependent kinase inhibitor 1C (p57, Kip2) | CDKN1C | chr11p15.5 |
| TAF6-like RNA polymerase II, p300/CBP-associated factor (PCAF)-associated factor, 65kDa | TAF6L | chr11q12.3 |
| TAF6-like RNA polymerase II, p300/CBP-associated factor (PCAF)-associated factor, 65kDa | TAF6L | chr11q12.3 |
| TAF6-like RNA polymerase II, p300/CBP-associated factor (PCAF)-associated factor, 65kDa | TAF6L | chr11q12.3 |
| Jun oncogene | JUN | chr1p32-p31 |
| collagen, type VI, alpha 2 | COL6A2 | chr21q22.3 |
| nuclear factor of activated T-cells, cytoplasmic, calcineurin-dependent 4 | NFATC4 | chr14q11.2 |
| cyclin-dependent kinase inhibitor 1C (p57, Kip2) | CDKN1C | chr11p15.5 |
| general transcription factor IIH, polypeptide 5 | GTF2H5 | chr6q25.3 |
| macrophage stimulating 1 (hepatocyte growth factor-like) /// macrophage stimulating, pseudogene 9 | MST1 /// MSTP9 | chr1p36.13 /// chr3p21 |
| heat shock 70kDa protein 6 (HSP70B') | HSPA6 | chr1q23 |
| mucin 5B, oligomeric mucus/gel-forming | MUC5B | chr11p15.5 |
| inducible T-cell co-stimulator ligand | ICOSLG | chr21q22.3 |
| excision repair cross-complementing rodent repair deficiency, complementation group 2 (xeroderma pigmentosum D) | ERCC2 | chr19q13.3 |
| Mitogen-activated protein kinase kinase 2 | MAP2K2 | chr19p13.3 |
| mitogen-activated protein kinase kinase 2 | MAP2K2 | chr19p13.3 |
| G0/G1switch 2 | G0S2 | chr1q32.2-q41 |
| ubiquitin-conjugating enzyme E2I (UBC9 homolog, yeast) | UBE2I | chr16p13.3 |
| Ubiquitin-conjugating enzyme E2I (UBC9 homolog, yeast) | UBE2I | chr16p13.3 |
| CD3d molecule, delta (CD3-TCR complex) | CD3D | chr11q23 |
| E1A binding protein p300 | EP300 | chr22q13.2 |
| TAF5-like RNA polymerase II, p300/CBP-associated factor (PCAF)-associated factor, 65kDa | TAF5L | chr1q42.13 |
| Vitamin D (1,25- dihydroxyvitamin D3) receptor | VDR | chr12q13.11 |
| mucin 1, cell surface associated | MUC1 | chr1q21 |
| secreted and transmembrane 1 | SECTM1 | chr17q25 |
| SWI/SNF related, matrix associated, actin dependent regulator of chromatin, subfamily a, member 4 | SMARCA4 | chr19p13.2 |
| SWI/SNF related, matrix associated, actin dependent regulator of chromatin, subfamily a, member 4 | SMARCA4 | chr19p13.2 |
| cytochrome c oxidase subunit Vb | COX5B | chr2cen-q13 |
| Cytochrome c oxidase subunit Vb | COX5B | chr2cen-q13 |
| trichohyalin | TCHH | chr1q21.3 |
| T cell receptor delta locus | TRD@ | chr14q11.2 |
| major histocompatibility complex, class II, DQ alpha 1 | HLA-DQA1 | chr6p21.3 |
| SMT3 suppressor of mif two 3 homolog 2 (S. cerevisiae) | SUMO2 | chr17q25.1 |
| polymerase (RNA) II (DNA directed) polypeptide E, 25kDa | POLR2E | chr19p13.3 |
| Protein phosphatase 3 (formerly 2B), catalytic subunit, gamma isoform | PPP3CC | chr8p21.3 |
| keratin 20 | KRT20 | chr17q21.2 |
| lysozyme (renal amyloidosis) | LYZ | chr12q15 |
| chemokine (C-C motif) ligand 8 | CCL8 | chr17q11.2 |
| Interferon-induced protein 44 | IFI44 | chr1p31.1 |
| nuclear factor of kappa light polypeptide gene enhancer in B-cells inhibitor, beta | NFKBIB | chr19q13.1 |
| mucin 6, oligomeric mucus/gel-forming /// similar to Mucin-6 precursor (Gastric mucin-6) | LOC100133432 /// MUC6 | chr11p15.5-p15.4 |
| Polymerase (RNA) II (DNA directed) polypeptide D | POLR2D | chr2q21 |
| GNAS complex locus | GNAS | chr20q13.3 |
| polymerase (RNA) II (DNA directed) polypeptide C, 33kDa | POLR2C | chr16q13-q21 |
| mucin 5AC, oligomeric mucus/gel-forming | MUC5AC | chr11p15.5 |
| heat shock protein 90kDa alpha (cytosolic), class A member 1 | HSP90AA1 | chr14q32.33 |
| v-Ki-ras2 Kirsten rat sarcoma viral oncogene homolog | KRAS | chr12p12.1 |
| heat shock protein 90kDa alpha (cytosolic), class B member 1 | HSP90AB1 | chr6p12 |
| SWI/SNF related, matrix associated, actin dependent regulator of chromatin, subfamily a, member 4 | SMARCA4 | chr19p13.2 |
| mucin 5AC, oligomeric mucus/gel-forming | MUC5AC | chr11p15.5 |
| insulin-like 3 (Leydig cell) /// Janus kinase 3 (a protein tyrosine kinase, leukocyte) | INSL3 /// JAK3 | chr19p13.1 /// chr19p13.2-p12 |
| Tyrosine aminotransferase | TAT | chr16q22.1 |
| selenium binding protein 1 | SELENBP1 | chr1q21-q22 |
| N-acetyltransferase 1 (arylamine N-acetyltransferase) | NAT1 | chr8p23.1-p21.3 |
| nuclear factor of kappa light polypeptide gene enhancer in B-cells inhibitor, beta | NFKBIB | chr19q13.1 |
| interferon-induced protein 44 | IFI44 | chr1p31.1 |
| serum amyloid A1 /// serum amyloid A2 | SAA1 /// SAA2 | chr11p15.1 /// chr11p15.1-p14 |
| major histocompatibility complex, class I, C | HLA-C | chr6p21.3 |
| cAMP responsive element binding protein 1 | CREB1 | chr2q34 |
| GNAS complex locus | GNAS | chr20q13.3 |
| insulin-like 3 (Leydig cell) /// Janus kinase 3 (a protein tyrosine kinase, leukocyte) | INSL3 /// JAK3 | chr19p13.1 /// chr19p13.2-p12 |
| calcium binding protein P22 | CHP | chr15q13.3 |
| mucin 3A, cell surface associated | MUC3A | chr7q22 |
| immunoglobulin lambda locus | IGL@ | chr22q11.1-q11.2 |
| fibronectin 1 | FN1 | chr2q34 |
| fibronectin 1 | FN1 | chr2q34 |
| SWI/SNF related, matrix associated, actin dependent regulator of chromatin, subfamily a, member 4 | SMARCA4 | chr19p13.2 |
| polymerase (RNA) II (DNA directed) polypeptide J, 13.3kDa /// polymerase (RNA) II (DNA directed) polypeptide J, 13.3kDa pseudogene /// DNA directed RNA polymerase II polypeptide J-related /// RPB11b2 protein /// similar to POLR2J4 protein | LOC100134053 /// POLR2J /// POLR2J2 /// POLR2J3 /// POLR2J4 | chr7p13 /// chr7q22.1 |
| cut-like homeobox 1 | CUX1 | chr7q22.1 |
| mitogen-activated protein kinase kinase kinase 1 | MAP3K1 | chr5q11.2 |
| SHC (Src homology 2 domain containing) transforming protein 1 | SHC1 | chr1q21 |
| CDK5 regulatory subunit associated protein 1-like 1 | CDKAL1 | chr6p22.3 |
| upstream transcription factor 2, c-fos interacting | USF2 | chr19q13 |
| immunoglobulin heavy locus /// immunoglobulin heavy constant alpha 1 /// immunoglobulin heavy constant alpha 2 (A2m marker) /// immunoglobulin heavy constant delta /// immunoglobulin heavy constant gamma 1 (G1m marker) /// immunoglobulin heavy constant gamma 2 (G2m marker) /// immunoglobulin heavy constant gamma 3 (G3m marker) /// immunoglobulin heavy constant mu /// immunoglobulin heavy variable group /// immunoglobulin heavy variable 4-31 /// hypothetical LOC100126583 /// similar to hCG2038920 | IGH@ /// IGHA1 /// IGHA2 /// IGHD /// IGHG1 /// IGHG2 /// IGHG3 /// IGHM /// IGHV4-31 /// IGHV@ /// LOC100126583 /// LOC100133739 | chr14q32.33 |
| high-mobility group box 1 | HMGB1 | chr13q12 |
| chemokine (C-X-C motif) ligand 5 | CXCL5 | chr4q12-q13 |
| periostin, osteoblast specific factor | POSTN | chr13q13.3 |
| fasciculation and elongation protein zeta 2 (zygin II) | FEZ2 | chr2p21 |
| BCL2-like 1 | BCL2L1 | chr20q11.21 |
| CD163 molecule | CD163 | chr12p13.3 |
| growth factor receptor-bound protein 2 | GRB2 | chr17q24-q25 |
| collagen, type III, alpha 1 (Ehlers-Danlos syndrome type IV, autosomal dominant) | COL3A1 | chr2q31 |
| superoxide dismutase 2, mitochondrial | SOD2 | chr6q25.3 |
| nuclear factor of activated T-cells 5, tonicity-responsive | NFAT5 | chr16q22.1 |
| Esterase D/formylglutathione hydrolase | ESD | chr13q14.1-q14.2 |
| esterase D/formylglutathione hydrolase | ESD | chr13q14.1-q14.2 |
| chemokine (C-X-C motif) ligand 5 | CXCL5 | chr4q12-q13 |
| immunoglobulin lambda locus /// immunoglobulin lambda constant 2 (Kern-Oz- marker) /// immunoglobulin lambda variable 2-14 | IGL@ /// IGLC2 /// IGLV2-14 | chr22q11.1-q11.2 /// chr22q11.2 |
| UDP glucuronosyltransferase 1 family, polypeptide A10 /// UDP glucuronosyltransferase 1 family, polypeptide A8 /// UDP glucuronosyltransferase 1 family, polypeptide A7 /// UDP glucuronosyltransferase 1 family, polypeptide A6 /// UDP glucuronosyltransferase 1 family, polypeptide A5 /// UDP glucuronosyltransferase 1 family, polypeptide A9 /// UDP glucuronosyltransferase 1 family, polypeptide A4 /// UDP glucuronosyltransferase 1 family, polypeptide A1 /// UDP glucuronosyltransferase 1 family, polypeptide A3 | UGT1A1 /// UGT1A10 /// UGT1A3 /// UGT1A4 /// UGT1A5 /// UGT1A6 /// UGT1A7 /// UGT1A8 /// UGT1A9 | chr2q37 |
| phosphoinositide-3-kinase, class 2, gamma polypeptide | PIK3C2G | chr12p12 |
| mediator complex subunit 14 | MED14 | chrXp11.4-p11.2 |
| major histocompatibility complex, class II, DQ beta 1 /// major histocompatibility complex, class II, DQ beta 2 /// major histocompatibility complex, class II, DR beta 1 /// major histocompatibility complex, class II, DR beta 2 (pseudogene) /// major histocompatibility complex, class II, DR beta 3 /// major histocompatibility complex, class II, DR beta 4 /// major histocompatibility complex, class II, DR beta 5 /// ribonuclease, RNase A family, 2 (liver, eosinophil-derived neurotoxin) /// zinc finger protein 749 /// hypothetical protein LOC730415 /// similar to Major histocompatibility complex, class II, DR beta 4 /// similar to major histocompatibility complex, class II, DQ beta 1 /// similar to HLA class II histocompatibility antigen, DR-W53 beta chain /// similar to hCG1992647 | hCG_1998957 /// HLA-DQB1 /// HLA-DQB2 /// HLA-DRB1 /// HLA-DRB2 /// HLA-DRB3 /// HLA-DRB4 /// HLA-DRB5 /// LOC100133484 /// LOC100133583 /// LOC100133661 /// LOC100133811 /// LOC730415 /// RNASE2 /// ZNF749 | chr14q24-q31 /// chr19q13.43 /// chr6p21 /// chr6p21.3 |
| nuclear receptor co-repressor 2 | NCOR2 | chr12q24 |
| Immunoglobulin lambda joining 3 | IGL@ | chr22q11.1-q11.2 |
| superoxide dismutase 2, mitochondrial | SOD2 | chr6q25.3 |
| immunoglobulin lambda locus /// immunoglobulin lambda constant 2 (Kern-Oz- marker) /// immunoglobulin lambda variable 2-14 | IGL@ /// IGLC2 /// IGLV2-14 | chr22q11.1-q11.2 /// chr22q11.2 |
| phosphoinositide-3-kinase, class 3 | PIK3C3 | chr18q12.3 |
| SMT3 suppressor of mif two 3 homolog 2 (S. cerevisiae) /// SMT3 suppressor of mif two 3 homolog 4 (S. cerevisiae) | SUMO2 /// SUMO4 | chr17q25.1 /// chr6q25 |
| intercellular adhesion molecule 1 (CD54), human rhinovirus receptor | ICAM1 | chr19p13.3-p13.2 |
| butyrophilin, subfamily 2, member A1 | BTN2A1 | chr6p22.1 |
| T cell receptor alpha locus /// T cell receptor alpha variable 20 /// T cell receptor alpha joining 17 /// T cell receptor alpha constant | TRA@ /// TRAC /// TRAJ17 /// TRAV20 | chr14q11 /// chr14q11.2 |
| major histocompatibility complex, class II, DQ beta 1 /// major histocompatibility complex, class II, DQ beta 2 /// major histocompatibility complex, class II, DR beta 1 /// major histocompatibility complex, class II, DR beta 2 (pseudogene) /// major histocompatibility complex, class II, DR beta 3 /// major histocompatibility complex, class II, DR beta 4 /// major histocompatibility complex, class II, DR beta 5 /// ribonuclease, RNase A family, 2 (liver, eosinophil-derived neurotoxin) /// zinc finger protein 749 /// hypothetical protein LOC730415 /// similar to Major histocompatibility complex, class II, DR beta 4 /// similar to major histocompatibility complex, class II, DQ beta 1 /// similar to HLA class II histocompatibility antigen, DR-W53 beta chain /// similar to hCG1992647 | hCG_1998957 /// HLA-DQB1 /// HLA-DQB2 /// HLA-DRB1 /// HLA-DRB2 /// HLA-DRB3 /// HLA-DRB4 /// HLA-DRB5 /// LOC100133484 /// LOC100133583 /// LOC100133661 /// LOC100133811 /// LOC730415 /// RNASE2 /// ZNF749 | chr14q24-q31 /// chr19q13.43 /// chr6p21 /// chr6p21.3 |
| T cell receptor alpha locus | TRA@ | chr14q11.2 |
| estrogen receptor 1 | ESR1 | chr6q25.1 |
| estrogen receptor 1 | ESR1 | chr6q25.1 |
| Nuclear receptor coactivator 2 | NCOA2 | chr8q13.3 |
| Solute carrier family 26, member 3 | SLC26A3 | chr7q31 |
| hydroxy-delta-5-steroid dehydrogenase, 3 beta- and steroid delta-isomerase 1 /// hydroxy-delta-5-steroid dehydrogenase, 3 beta- and steroid delta-isomerase 2 /// 3-beta-hydroxysteroid dehydrogenase, tissue-type heart /// similar to 3 beta-hydroxysteroid dehydrogenase/Delta 5-->4-isomerase type 1 (3-beta-HSD I) | HSD3B1 /// HSD3B2 /// LOC128102 /// LOC391081 | chr1p12 /// chr1p13.1 |
| SWI/SNF related, matrix associated, actin dependent regulator of chromatin, subfamily a, member 4 | SMARCA4 | chr19p13.2 |
| ATPase, Ca++ transporting, plasma membrane 1 | ATP2B1 | chr12q21.3 |
| upstream transcription factor 2, c-fos interacting | USF2 | chr19q13 |
| strawberry notch homolog 2 (Drosophila) | SBNO2 | chr19p13.3 |
| YME1-like 1 (S. cerevisiae) /// T-cell antigen receptor-alpha (TCRA) mRNA, partial putative cds | TRA@ | chr14q11.2 |
| immunoglobulin lambda-like polypeptide 3 | IGLL3 | chr22q11.2|22q11.23 |
| YME1-like 1 (S. cerevisiae) | TRA@ | chr14q11.2 |
| T cell receptor alpha locus /// T cell receptor delta locus | TRA@ /// TRD@ | chr14q11.2 |
| mitogen-activated protein kinase kinase 7 | MAP2K7 | chr19p13.3-p13.2 |
| TAF4b RNA polymerase II, TATA box binding protein (TBP)-associated factor, 105kDa | TAF4B | chr18q11.2 |
| CD163 molecule | CD163 | chr12p13.3 |
| protein kinase, cAMP-dependent, catalytic, alpha | PRKACA | chr19p13.1 |
| polymerase (RNA) II (DNA directed) polypeptide J, 13.3kDa pseudogene /// DNA directed RNA polymerase II polypeptide J-related /// RPB11b2 protein | POLR2J2 /// POLR2J3 /// POLR2J4 | chr7p13 /// chr7q22.1 |
| interleukin 1 receptor antagonist | IL1RN | chr2q14.2 |
| interleukin 1 receptor antagonist | IL1RN | chr2q14.2 |
| interleukin 1 receptor antagonist | IL1RN | chr2q14.2 |
| polymerase (RNA) II (DNA directed) polypeptide C, 33kDa | POLR2C | chr16q13-q21 |
| macrophage stimulating 1 (hepatocyte growth factor-like) | MST1 | chr3p21 |
| nuclear receptor subfamily 3, group C, member 1 (glucocorticoid receptor) | NR3C1 | chr5q31.3 |
| metallothionein 1E /// metallothionein 1H /// metallothionein 1M /// metallothionein 1 pseudogene 2 | MT1E /// MT1H /// MT1M /// MT1P2 | chr16q13 /// chr1q43 |
| immunoglobulin lambda locus /// immunoglobulin lambda variable 3-19 | IGL@ /// IGLV3-19 | chr22q11.1-q11.2 /// chr22q11.2 |
| leukotriene B4 receptor | LTB4R | chr14q11.2-q12 |
| immunoglobulin lambda locus | IGL@ | chr22q11.1-q11.2 |
| immunoglobulin lambda locus | IGL@ | chr22q11.1-q11.2 |
| fibronectin 1 | FN1 | chr2q34 |
| high-mobility group box 1 /// high-mobility group (nonhistone chromosomal) protein 1-like 1 /// high-mobility group (nonhistone chromosomal) protein 1-like 10 /// CCCTC-binding factor (zinc finger protein)-like /// similar to high-mobility group (nonhistone chromosomal) protein 1-like 10 /// similar to hCG26831 | CTCFL /// HMG1L1 /// HMG1L10 /// HMGB1 /// LOC100130561 /// LOC100132863 | chr13q12 /// chr20q13.31 /// chr20q13.32 /// chr22q12.1 /// chr9q33.2 |
| major histocompatibility complex, class I, C /// immunoglobulin kappa constant /// immunoglobulin kappa variable 1-5 /// similar to Ig kappa chain V-I region HK101 precursor /// similar to Ig kappa chain V-I region HK102 precursor /// similar to hCG26659 | HLA-C /// IGKC /// IGKV1-5 /// LOC100130100 /// LOC647506 /// LOC650405 /// LOC652493 /// LOC652694 | chr2p12 /// chr2q14.1 /// chr6p21.3 |
| major histocompatibility complex, class I, B /// major histocompatibility complex, class I, C /// MHC class I polypeptide-related sequence A /// MHC class I polypeptide-related sequence B | HLA-B /// HLA-C /// MICA /// MICB /// XXbac-BPG181B23.1 | chr6p21.3 /// chr6p21.33 |
| YME1-like 1 (S. cerevisiae) | TRA@ | chr14q11.2 |
| immunoglobulin heavy constant alpha 1 /// immunoglobulin heavy constant gamma 1 (G1m marker) /// immunoglobulin heavy constant gamma 3 (G3m marker) /// immunoglobulin heavy constant mu /// immunoglobulin heavy variable group /// immunoglobulin heavy variable 4-31 /// similar to hCG2038920 | IGHA1 /// IGHG1 /// IGHG3 /// IGHM /// IGHV4-31 /// IGHV@ /// LOC100133739 | chr14q32.33 |
| immunoglobulin heavy constant alpha 1 /// immunoglobulin heavy constant delta /// immunoglobulin heavy constant gamma 1 (G1m marker) /// immunoglobulin heavy constant gamma 2 (G2m marker) /// immunoglobulin heavy constant gamma 3 (G3m marker) /// immunoglobulin heavy constant mu /// immunoglobulin heavy variable 4-31 /// similar to Ig heavy chain V-III region VH26 precursor | IGHA1 /// IGHD /// IGHG1 /// IGHG2 /// IGHG3 /// IGHM /// IGHV4-31 /// LOC652494 | chr14q32.33 |
| immunoglobulin lambda locus | IGL@ | chr22q11.1-q11.2 |
| immunoglobulin lambda locus | IGL@ | chr22q11.1-q11.2 |
| chemokine (C-C motif) ligand 2 | CCL2 | chr17q11.2-q12 |
| major histocompatibility complex, class II, DQ beta 1 /// major histocompatibility complex, class II, DQ beta 2 /// major histocompatibility complex, class II, DR beta 1 /// major histocompatibility complex, class II, DR beta 2 (pseudogene) /// major histocompatibility complex, class II, DR beta 3 /// major histocompatibility complex, class II, DR beta 4 /// major histocompatibility complex, class II, DR beta 5 /// ribonuclease, RNase A family, 2 (liver, eosinophil-derived neurotoxin) /// zinc finger protein 749 /// similar to Nonsecretory ribonuclease precursor (Ribonuclease US) (Eosinophil-derived neurotoxin) (RNase UpI-2) (Ribonuclease 2) (RNase 2) /// hypothetical protein LOC730415 /// similar to Major histocompatibility complex, class II, DR beta 4 /// similar to major histocompatibility complex, class II, DQ beta 1 /// similar to HLA class II histocompatibility antigen, DR-W53 beta chain /// similar to hCG1992647 | hCG_1998957 /// HLA-DQB1 /// HLA-DQB2 /// HLA-DRB1 /// HLA-DRB2 /// HLA-DRB3 /// HLA-DRB4 /// HLA-DRB5 /// LOC100133484 /// LOC100133583 /// LOC100133661 /// LOC100133811 /// LOC643332 /// LOC730415 /// RNASE2 /// ZNF749 | chr14q11.2 /// chr14q24-q31 /// chr19q13.43 /// chr6p21 /// chr6p21.3 |
| mucin 8 | MUC8 | chr12q24.3 |
| UDP glucuronosyltransferase 2 family, polypeptide B15 | UGT2B15 | chr4q13 |
| kallikrein 1 | KLK1 | chr19q13.3 |
| TAF1 RNA polymerase II, TATA box binding protein (TBP)-associated factor, 250kDa | TAF1 | chrXq13.1 |
| chemokine (C-C motif) ligand 13 | CCL13 | chr17q11.2 |
| glycophorin B (MNS blood group) /// glycophorin E | GYPB /// GYPE | chr4q28-q31 /// chr4q31.1 |
| superoxide dismutase 2, mitochondrial | SOD2 | chr6q25.3 |
| immunoglobulin lambda locus /// immunoglobulin lambda variable 3-19 /// immunoglobulin lambda variable 2-18 | IGL@ /// IGLV2-18 /// IGLV3-19 | chr22q11.1-q11.2 /// chr22q11.2 |
| Immunoglobulin lambda joining 3 | IGL@ | chr22q11.1-q11.2 |
| Immunoglobulin lambda joining 3 | IGL@ | chr22q11.1-q11.2 |
| interleukin 17A | IL17A | chr6p12 |
| cyclin-dependent kinase inhibitor 1C (p57, Kip2) | CDKN1C | chr11p15.5 |
| major histocompatibility complex, class II, DO alpha | HLA-DOA | chr6p21.3 |
| Fc fragment of IgG, high affinity Ia, receptor (CD64) | FCGR1A | chr1q21.2-q21.3 |
| Fc fragment of IgG, high affinity Ia, receptor (CD64) | FCGR1A | chr1q21.2-q21.3 |
| TAF1 RNA polymerase II, TATA box binding protein (TBP)-associated factor, 250kDa | TAF1 | chrXq13.1 |
| major histocompatibility complex, class II, DO alpha | HLA-DOA | chr6p21.3 |
| Netrin 2-like (chicken) | NTN2L | chr16p13.3 |
| SHC (Src homology 2 domain containing) transforming protein 1 | SHC1 | chr1q21 |
| YME1-like 1 (S. cerevisiae) /// T cell receptor, clone IGRA15 /// T-cell receptor alpha chain V-region /// T-cell antigen receptor-alpha (TCRA) mRNA, partial putative cds | TRA@ | chr14q11.2 |
| GNAS complex locus | GNAS | chr20q13.3 |
| GNAS complex locus | GNAS | chr20q13.3 |
| mucin 7, secreted | MUC7 | chr4q13-q21 |
| YME1-like 1 (S. cerevisiae) | TRA@ | chr14q11.2 |
| YME1-like 1 (S. cerevisiae) | TRA@ | chr14q11.2 |
| mucin 4, cell surface associated | MUC4 | chr3q29 |
| mucin 4, cell surface associated | MUC4 | chr3q29 |
| mucin 3A, cell surface associated | MUC3A | chr7q22 |
| mediator complex subunit 14 | MED14 | chrXp11.4-p11.2 |
| Immunoglobulin lambda joining 3 | IGL@ | chr22q11.1-q11.2 |
| T cell receptor alpha locus /// T cell receptor delta locus | TRA@ /// TRD@ | chr14q11.2 |
| Immunoglobulin lambda joining 3 | IGL@ | chr22q11.1-q11.2 |
| estrogen receptor 1 | ESR1 | chr6q25.1 |
| UDP glucuronosyltransferase 2 family, polypeptide B15 | UGT2B15 | chr4q13 |
| mucin 5AC, oligomeric mucus/gel-forming | MUC5AC | chr11p15.5 |
| mucin 5AC, oligomeric mucus/gel-forming | MUC5AC | chr11p15.5 |
| estrogen receptor 1 | ESR1 | chr6q25.1 |
| Interleukin 8 | IL8 | chr4q13-q21 |
| immunoglobulin heavy locus /// immunoglobulin heavy constant alpha 1 /// immunoglobulin heavy constant alpha 2 (A2m marker) /// immunoglobulin heavy constant delta /// immunoglobulin heavy constant gamma 1 (G1m marker) /// immunoglobulin heavy constant gamma 3 (G3m marker) /// immunoglobulin heavy constant gamma 4 (G4m marker) /// immunoglobulin heavy constant mu /// immunoglobulin heavy variable 4-31 /// similar to hCG1812074 /// similar to Ig heavy chain V-II region ARH-77 precursor /// hypothetical LOC100126583 | IGH@ /// IGHA1 /// IGHA2 /// IGHD /// IGHG1 /// IGHG3 /// IGHG4 /// IGHM /// IGHV4-31 /// LOC100126583 /// LOC100134331 /// LOC642131 /// LOC652128 | chr14q32.33 /// chr15q11.2 |
| immunoglobulin lambda locus | IGL@ | chr22q11.1-q11.2 |
| immunoglobulin lambda locus /// immunoglobulin lambda constant 1 (Mcg marker) /// immunoglobulin lambda variable 2-23 /// immunoglobulin lambda variable 2-18 /// immunoglobulin lambda variable 2-11 | IGL@ /// IGLC1 /// IGLV2-11 /// IGLV2-18 /// IGLV2-23 | chr22q11.1-q11.2 /// chr22q11.2 |
| immunoglobulin heavy locus /// immunoglobulin heavy constant alpha 1 /// immunoglobulin heavy constant alpha 2 (A2m marker) /// immunoglobulin heavy constant delta /// immunoglobulin heavy constant gamma 1 (G1m marker) /// immunoglobulin heavy constant gamma 3 (G3m marker) /// immunoglobulin heavy constant gamma 4 (G4m marker) /// immunoglobulin heavy constant mu /// immunoglobulin heavy variable 4-31 /// similar to hCG1812074 /// similar to Ig heavy chain V-II region ARH-77 precursor /// hypothetical LOC100126583 | IGH@ /// IGHA1 /// IGHA2 /// IGHD /// IGHG1 /// IGHG3 /// IGHG4 /// IGHM /// IGHV4-31 /// LOC100126583 /// LOC100134331 /// LOC642131 /// LOC652128 | chr14q32.33 /// chr15q11.2 |
| aldolase B, fructose-bisphosphate | ALDOB | chr9q21.3-q22.2 |
| immunoglobulin lambda locus | IGL@ | chr22q11.1-q11.2 |
| immunoglobulin heavy locus /// immunoglobulin heavy constant alpha 1 /// immunoglobulin heavy constant alpha 2 (A2m marker) /// immunoglobulin heavy constant gamma 1 (G1m marker) /// immunoglobulin heavy constant gamma 2 (G2m marker) /// immunoglobulin heavy constant gamma 3 (G3m marker) /// immunoglobulin heavy constant mu /// immunoglobulin heavy variable 4-31 /// similar to Ig heavy chain V-III region VH26 precursor /// hypothetical LOC100126583 /// similar to hCG2038920 | IGH@ /// IGHA1 /// IGHA2 /// IGHG1 /// IGHG2 /// IGHG3 /// IGHM /// IGHV4-31 /// LOC100126583 /// LOC100133739 /// LOC652494 | chr14q32.33 |
| mucin 8 | MUC8 | chr12q24.3 |
| immunoglobulin heavy constant alpha 1 /// immunoglobulin heavy constant gamma 1 (G1m marker) /// immunoglobulin heavy constant gamma 3 (G3m marker) /// immunoglobulin heavy constant mu /// similar to Ig heavy chain V-III region VH26 precursor | IGHA1 /// IGHG1 /// IGHG3 /// IGHM /// LOC652494 | chr14q32.33 |
| interleukin 15 | IL15 | chr4q31 |
| YME1-like 1 (S. cerevisiae) /// T cell receptor V alpha gene segment V-alpha-w24, clone IGRa02 | TRA@ | chr14q11.2 |
| polymerase (RNA) II (DNA directed) polypeptide A, 220kDa | POLR2A | chr17p13.1 |
| polymerase (RNA) II (DNA directed) polypeptide A, 220kDa | POLR2A | chr17p13.1 |
| collagen, type I, alpha 1 | COL1A1 | chr17q21.33 |
| major histocompatibility complex, class I, E | HLA-E | chr6p21.3 |
| major histocompatibility complex, class II, DM alpha | HLA-DMA | chr6p21.3 |
| son of sevenless homolog 2 (Drosophila) | SOS2 | chr14q21 |
| son of sevenless homolog 2 (Drosophila) | SOS2 | chr14q21 |
| phosphoinositide-3-kinase, catalytic, beta polypeptide | PIK3CB | chr3q22.3 |
| son of sevenless homolog 2 (Drosophila) | SOS2 | chr14q21 |
| GNAS complex locus | GNAS | chr20q13.3 |
| SWI/SNF related, matrix associated, actin dependent regulator of chromatin, subfamily a, member 2 | SMARCA2 | chr9p22.3 |
| alpha-2-macroglobulin | A2M | chr12p13.3-p12.3 |
| actin related protein 2/3 complex, subunit 4, 20kDa /// tubulin tyrosine ligase-like family, member 3 | ARPC4 /// TTLL3 | chr3p25.3 |
| actin related protein 2/3 complex, subunit 4, 20kDa /// tubulin tyrosine ligase-like family, member 3 | ARPC4 /// TTLL3 | chr3p25.3 |
| polymerase (RNA) II (DNA directed) polypeptide E, 25kDa | POLR2E | chr19p13.3 |
| SMAD family member 3 | SMAD3 | chr15q22.33 |
| LYR motif containing 4 | LYRM4 | chr6p25.1 |
| chromosome 11 open reading frame 30 | C11orf30 | chr11q13.5 |
| peroxisome proliferator-activated receptor gamma, coactivator 1 alpha | PPARGC1A | chr4p15.1 |
| heat shock 70kDa protein 14 | HSPA14 | chr10p13 |
| cyclin-dependent kinase inhibitor 1C (p57, Kip2) | CDKN1C | chr11p15.5 |
| cyclin-dependent kinase inhibitor 1C (p57, Kip2) | CDKN1C | chr11p15.5 |
| fibrinogen gamma chain | FGG | chr4q28 |
| PDZK1 interacting protein 1 | PDZK1IP1 | chr1p33 |
| CDK5 regulatory subunit associated protein 1-like 1 | CDKAL1 | chr6p22.3 |
| interleukin 23, alpha subunit p19 | IL23A | chr12q13.2 |
| nucleotide-binding oligomerization domain containing 2 | NOD2 | chr16q21 |
| leukotriene B4 receptor 2 | LTB4R2 | chr14q11.2-q12 |
| TAF7-like RNA polymerase II, TATA box binding protein (TBP)-associated factor, 50kDa | TAF7L | chrXq22.1 |
| transforming growth factor, beta 2 | TGFB2 | chr1q41 |
| transforming growth factor, beta 2 | TGFB2 | chr1q41 |
| ATG16 autophagy related 16-like 1 (S. cerevisiae) | ATG16L1 | chr2q37.1 |
| phosphoinositide-3-kinase, regulatory subunit 5 | PIK3R5 | chr17p13.1 |
| interleukin 19 | IL19 | chr1q32.2 |
| NADH dehydrogenase (ubiquinone) 1 alpha subcomplex, 13 | NDUFA13 | chr19p13.2 |
| interleukin 25 | IL25 | chr14q11.2 |
| toll-like receptor 4 | TLR4 | chr9q32-q33 |
| tumor necrosis factor (ligand) superfamily, member 15 | TNFSF15 | chr9q32 |
| interleukin 26 | IL26 | chr12q15 |
| interleukin 22 | IL22 | chr12q15 |
| interleukin 21 | IL21 | chr4q26-q27 |
| UDP glucuronosyltransferase 1 family, polypeptide A10 /// UDP glucuronosyltransferase 1 family, polypeptide A8 /// UDP glucuronosyltransferase 1 family, polypeptide A7 /// UDP glucuronosyltransferase 1 family, polypeptide A6 | UGT1A10 /// UGT1A6 /// UGT1A7 /// UGT1A8 | chr2q37 |
| UDP glucuronosyltransferase 1 family, polypeptide A8 /// UDP glucuronosyltransferase 1 family, polypeptide A6 /// UDP glucuronosyltransferase 1 family, polypeptide A9 | UGT1A6 /// UGT1A8 /// UGT1A9 | chr2q37 |
| butyrophilin-like 2 (MHC class II associated) | BTNL2 | chr6p21.3 |
| major histocompatibility complex, class II, DQ beta 1 /// major histocompatibility complex, class II, DQ beta 2 /// major histocompatibility complex, class II, DR beta 1 /// major histocompatibility complex, class II, DR beta 2 (pseudogene) /// major histocompatibility complex, class II, DR beta 3 /// major histocompatibility complex, class II, DR beta 4 /// major histocompatibility complex, class II, DR beta 5 /// ribonuclease, RNase A family, 2 (liver, eosinophil-derived neurotoxin) /// zinc finger protein 749 /// hypothetical protein LOC730415 /// similar to HLA class II histocompatibility antigen, DRB1-7 beta chain precursor (MHC class I antigen DRB1*7) (DR-7) (DR7) /// similar to Major histocompatibility complex, class II, DR beta 4 /// similar to major histocompatibility complex, class II, DQ beta 1 /// similar to HLA class II histocompatibility antigen, DR-W53 beta chain /// similar to hCG1992647 | hCG_1998957 /// HLA-DQB1 /// HLA-DQB2 /// HLA-DRB1 /// HLA-DRB2 /// HLA-DRB3 /// HLA-DRB4 /// HLA-DRB5 /// LOC100133484 /// LOC100133583 /// LOC100133661 /// LOC100133811 /// LOC730415 /// LOC731718 /// RNASE2 /// ZNF749 | chr14q24-q31 /// chr19q13.43 /// chr6p21 /// chr6p21.3 |
| general transcription factor IIH, polypeptide 2, 44kDa /// general transcription factor IIH, polypeptide 2, 44kDa-like | DKFZP686M0199 /// GTF2H2 /// LOC728340 /// LOC730394 | chr5q12.2-q13.3 /// chr5q13.2 |
| TAF9B RNA polymerase II, TATA box binding protein (TBP)-associated factor, 31kDa | TAF9B | chrXq13.1-q21.1 |
| TAF9B RNA polymerase II, TATA box binding protein (TBP)-associated factor, 31kDa | TAF9B | chrXq13.1-q21.1 |
| TAF9B RNA polymerase II, TATA box binding protein (TBP)-associated factor, 31kDa /// similar to transcription associated factor TAFII31L | LOC728198 /// TAF9B | chrXq13.1-q21.1 |
| zinc finger, CCCH-type with G patch domain | ZGPAT | chr20q13.3 |
| heat shock 70kDa protein 8 | HSPA8 | chr11q24.1 |
| TAF6-like RNA polymerase II, p300/CBP-associated factor (PCAF)-associated factor, 65kDa | TAF6L | chr11q12.3 |
| sulfotransferase family, cytosolic, 1A, phenol-preferring, member 3 /// sulfotransferase family, cytosolic, 1A, phenol-preferring, member 4 | SULT1A3 /// SULT1A4 | chr16p11.2 |
| general transcription factor IIH, polypeptide 3, 34kDa | GTF2H3 | chr12q24.31 |
| mucin 5AC, oligomeric mucus/gel-forming /// mucin 5B, oligomeric mucus/gel-forming | MUC5AC /// MUC5B | chr11p15.5 |
| LYR motif containing 4 | LYRM4 | chr6p25.1 |
| chromosome 11 open reading frame 30 | C11orf30 | chr11q13.5 |
| chromosome 11 open reading frame 30 | C11orf30 | chr11q13.5 |
| interleukin 22 | IL22 | chr12q15 |
| growth factor receptor-bound protein 2 | GRB2 | chr17q24-q25 |
| solute carrier family 22, member 23 | SLC22A23 | chr6p25.2 |
| intelectin 1 (galactofuranose binding) | ITLN1 | chr1q22-q23.5 |
| general transcription factor IIH, polypeptide 2, 44kDa | GTF2H2 | chr5q12.2-q13.3 |
| interleukin 20 | IL20 | chr1q32 |
| steroid receptor RNA activator 1 | SRA1 | chr5q31.3 |
| heat shock 70kDa protein 8 | HSPA8 | chr11q24.1 |
| toll-like receptor 4 | TLR4 | chr9q32-q33 |
| immunoglobulin lambda locus | IGL@ | chr22q11.1-q11.2 |
| YME1-like 1 (S. cerevisiae) /// T cell receptor V alpha gene segment V-alpha-w23, clone IGRa01 /// Kinesin family member C3 | KIFC3 /// TRA@ | chr14q11.2 /// chr16q13-q21 |
| TAF7-like RNA polymerase II, TATA box binding protein (TBP)-associated factor, 50kDa | TAF7L | chrXq22.1 |
| nuclear factor of activated T-cells, cytoplasmic, calcineurin-dependent 2 | NFATC2 | chr20q13.2-q13.3 |
| Mitogen-activated protein kinase 1 | MAPK1 | chr22q11.2|22q11.21 |
| mitogen-activated protein kinase 1 | MAPK1 | chr22q11.2|22q11.21 |
| high-mobility group box 1 | HMGB1 | chr13q12 |
| high-mobility group box 1 | HMGB1 | chr13q12 |
| transforming growth factor, beta receptor I (activin A receptor type II-like kinase, 53kDa) | TGFBR1 | chr9q22 |
| FK506 binding protein 5 | FKBP5 | chr6p21.3-p21.2 |
| FK506 binding protein 5 | FKBP5 | chr6p21.3-p21.2 |
| steroid receptor RNA activator 1 | SRA1 | chr5q31.3 |
| solute carrier family 26 (sulfate transporter), member 2 | SLC26A2 | chr5q31-q34 |
| solute carrier family 26 (sulfate transporter), member 2 | SLC26A2 | chr5q31-q34 |
| nuclear factor of activated T-cells 5, tonicity-responsive | NFAT5 | chr16q22.1 |
| neuroblastoma RAS viral (v-ras) oncogene homolog | NRAS | chr1p13.2 |
| muscle RAS oncogene homolog | MRAS | chr3q22.3 |
| signal transducer and activator of transcription 3 (acute-phase response factor) | STAT3 | chr17q21.31 |
| General transcription factor IIA, 1, 19/37kDa | GTF2A1 | chr14q31.1 |
| mediator complex subunit 1 | MED1 | chr17q12-q21.1 |
| mediator complex subunit 1 | MED1 | chr17q12-q21.1 |
| cAMP responsive element binding protein 1 | CREB1 | chr2q34 |
| cAMP responsive element binding protein 1 | CREB1 | chr2q34 |
| Mitogen-activated protein kinase 9 | MAPK9 | chr5q35 |
| mitogen-activated protein kinase kinase kinase 1 | MAP3K1 | chr5q11.2 |
| mitogen-activated protein kinase kinase 7 | MAP2K7 | chr19p13.3-p13.2 |
| TAF9B RNA polymerase II, TATA box binding protein (TBP)-associated factor, 31kDa /// similar to transcription associated factor TAFII31L | LOC728198 /// TAF9B | chrXq13.1-q21.1 |
| mitogen-activated protein kinase 8 | MAPK8 | chr10q11.22 |
| mitogen-activated protein kinase 8 | MAPK8 | chr10q11.22 |
| mitogen-activated protein kinase kinase 7 | MAP2K7 | chr19p13.3-p13.2 |
| phosphoinositide-3-kinase, class 2, alpha polypeptide | PIK3C2A | chr11p15.5-p14 |
| polymeric immunoglobulin receptor | PIGR | chr1q31-q41 |
| SMAD family member 2 | SMAD2 | chr18q21.1 |
| mucin 12, cell surface associated | MUC12 | chr7q22 |
| major histocompatibility complex, class II, DO alpha | HLA-DOA | chr6p21.3 |
| heat shock 70kDa protein 14 | HSPA14 | chr10p13 |
| Nuclear factor of activated T-cells, cytoplasmic, calcineurin-dependent 2 | NFATC2 | chr20q13.2-q13.3 |
| TAF1 RNA polymerase II, TATA box binding protein (TBP)-associated factor, 250kDa | TAF1 | chrXq13.1 |
| mucin 15, cell surface associated | MUC15 | chr11p14.3 |
| mucin 15, cell surface associated | MUC15 | chr11p14.3 |
| POU class 2 homeobox 1 | POU2F1 | chr1q22-q23 |
| son of sevenless homolog 1 (Drosophila) | SOS1 | chr2p22-p21 |
| phosphoinositide-3-kinase, regulatory subunit 5 | PIK3R5 | chr17p13.1 |
| heat shock 70kDa protein 14 | HSPA14 | chr10p13 |
| Janus kinase 3 (a protein tyrosine kinase, leukocyte) | JAK3 | chr19p13.1 |
| TAF15 RNA polymerase II, TATA box binding protein (TBP)-associated factor, 68kDa | TAF15 | chr17q11.1-q11.2 |
| TAF15 RNA polymerase II, TATA box binding protein (TBP)-associated factor, 68kDa | TAF15 | chr17q11.1-q11.2 |
| esterase D/formylglutathione hydrolase | ESD | chr13q14.1-q14.2 |
| GNAS complex locus | GNAS | chr20q13.3 |
| CREB binding protein (Rubinstein-Taybi syndrome) | CREBBP | chr16p13.3 |
| POU class 2 homeobox 2 | POU2F2 | chr19q13.2 |
| nuclear factor of kappa light polypeptide gene enhancer in B-cells inhibitor, beta | NFKBIB | chr19q13.1 |
| TAF9B RNA polymerase II, TATA box binding protein (TBP)-associated factor, 31kDa | TAF9B | chrXq13.1-q21.1 |
| progesterone receptor | PGR | chr11q22-q23 |
| growth factor receptor-bound protein 2 | GRB2 | chr17q24-q25 |
| SWI/SNF related, matrix associated, actin dependent regulator of chromatin, subfamily a, member 2 | SMARCA2 | chr9p22.3 |
| chromosome 13 open reading frame 31 | C13orf31 | chr13q14.11 |
| inducible T-cell co-stimulator ligand | ICOSLG | chr21q22.3 |
| collagen, type I, alpha 2 | COL1A2 | chr7q22.1 |
| GNAS complex locus | GNAS | chr20q13.3 |
| Pseudouridylate synthase 10 | PUS10 | chr2p16.1 |
| Phosphoinositide-3-kinase, regulatory subunit 2 (beta) | PIK3R2 | chr19q13.2-q13.4 |
| syntaxin 4 | STX4 | chr16p11.2 |
| leucine-rich repeat kinase 2 | LRRK2 | chr12q12 |
| mitogen-activated protein kinase 8 | MAPK8 | chr10q11.22 |
| BCL2-associated athanogene | BAG1 | chr9p12 |
| POU class 2 homeobox 2 | POU2F2 | chr19q13.2 |
| POU class 2 homeobox 1 | POU2F1 | chr1q22-q23 |
| Mitogen-activated protein kinase 1 | MAPK1 | chr22q11.2|22q11.21 |
| heat shock 70kDa protein 5 (glucose-regulated protein, 78kDa) | HSPA5 | chr9q33-q34.1 |
| TAF6-like RNA polymerase II, p300/CBP-associated factor (PCAF)-associated factor, 65kDa | TAF6L | chr11q12.3 |
| Chemokine (C-X-C motif) ligand 2 | CXCL2 | chr4q21 |
| Glucuronidase, beta | GUSB | chr7q21.11 |
| Immunoglobulin lambda joining 3 | IGL@ | chr22q11.1-q11.2 |
| V-rel reticuloendotheliosis viral oncogene homolog A, nuclear factor of kappa light polypeptide gene enhancer in B-cells 3, p65 (avian) | RELA | chr11q13 |
| son of sevenless homolog 1 (Drosophila) | SOS1 | chr2p22-p21 |
| hypothetical gene supported by AK128882 | LOC441108 | chr5q31.1 |
| Solute carrier family 20 (phosphate transporter), member 1 | SLC20A1 | chr2q11-q14 |
| BCL2-like 1 | BCL2L1 | chr20q11.21 |
| Nuclear receptor coactivator 1 | NCOA1 | chr2p23 |
| protein phosphatase 3 (formerly 2B), regulatory subunit B, beta isoform | PPP3R2 | chr9q31.1 |
| Nicotinamide N-methyltransferase | NNMT | chr11q23.1 |
| Fatty acid binding protein 1, liver | FABP1 | chr2p11 |
| nuclear factor of activated T-cells, cytoplasmic, calcineurin-dependent 2 | NFATC2 | chr20q13.2-q13.3 |
| mucin 12, cell surface associated | MUC12 | chr7q22 |
| phosphoinositide-3-kinase, catalytic, alpha polypeptide | PIK3CA | chr3q26.3 |
| toll-like receptor 4 | TLR4 | chr9q32-q33 |
| heat shock 70kDa protein 9 (mortalin) | HSPA9 | chr5q31.1 |
| Hypothetical gene supported by AK128882 | LOC441108 | chr5q31.1 |
| mucin 17, cell surface associated | MUC17 | chr7q22.1 |
| mucin 17, cell surface associated | MUC17 | chr7q22.1 |
| Collagen, type III, alpha 1 (Ehlers-Danlos syndrome type IV, autosomal dominant) | COL3A1 | chr2q31 |
| ATG16 autophagy related 16-like 1 (S. cerevisiae) | ATG16L1 | chr2q37.1 |
| Polymerase (RNA) II (DNA directed) polypeptide F | POLR2F | chr22q13.1 |
| UDP glucuronosyltransferase 1 family, polypeptide A6 | UGT1A6 | chr2q37 |
| Pseudouridylate synthase 10 | PUS10 | chr2p16.1 |
| Annexin A1 | ANXA1 | chr9q12-q21.2|9q12-q21.2 |
| solute carrier family 22, member 23 | SLC22A23 | chr6p25.2 |
| Selenium binding protein 1 | SELENBP1 | chr1q21-q22 |
| CD9 molecule | CD9 | chr12p13.3 |
| CD9 molecule | CD9 | chr12p13.3 |
| sulfotransferase family, cytosolic, 1A, phenol-preferring, member 3 /// GIY-YIG domain containing 2 /// sulfotransferase family, cytosolic, 1A, phenol-preferring, member 4 /// GIY-YIG domain containing 1 | GIYD1 /// GIYD2 /// SULT1A3 /// SULT1A4 | chr16p11.2 |
| ubiquitin-conjugating enzyme E2I (UBC9 homolog, yeast) | UBE2I | chr16p13.3 |
| Mitogen-activated protein kinase kinase kinase 7 interacting protein 1 | MAP3K7IP1 | chr22q13.1 |
| Heat shock 70kDa protein 1-like | HSPA1L | chr6p21.3 |
| Immunoglobulin lambda joining 3 | IGL@ | chr22q11.1-q11.2 |
| von Willebrand factor | VWF | chr12p13.3 |
| YME1-like 1 (S. cerevisiae) | TRA@ | chr14q11.2 |
| TAF15 RNA polymerase II, TATA box binding protein (TBP)-associated factor, 68kDa | TAF15 | chr17q11.1-q11.2 |
| regenerating islet-derived 3 alpha /// regenerating islet-derived 3 gamma | REG3A /// REG3G | chr2p12 |
| nuclear receptor co-repressor 1 | NCOR1 | chr17p11.2 |
| immunoglobulin lambda locus /// immunoglobulin lambda constant 1 (Mcg marker) /// immunoglobulin lambda variable 3-21 /// immunoglobulin lambda variable 3-12 | IGL@ /// IGLC1 /// IGLV3-12 /// IGLV3-21 | chr22q11.1-q11.2 /// chr22q11.2 |
| immunoglobulin lambda locus | IGL@ | chr22q11.1-q11.2 |
| YME1-like 1 (S. cerevisiae) | TRA@ | chr14q11.2 |
| YME1-like 1 (S. cerevisiae) | TRA@ | chr14q11.2 |
| YME1-like 1 (S. cerevisiae) | TRA@ | chr14q11.2 |
| Alanyl (membrane) aminopeptidase (aminopeptidase N, aminopeptidase M, microsomal aminopeptidase, CD13, p150) | ANPEP | chr15q25-q26 |
| Alanyl (membrane) aminopeptidase (aminopeptidase N, aminopeptidase M, microsomal aminopeptidase, CD13, p150) | ANPEP | chr15q25-q26 |
| chromosome 11 open reading frame 30 | C11orf30 | chr11q13.5 |
| immunoglobulin lambda locus /// interleukin 8 /// immunoglobulin lambda variable 1-44 /// immunoglobulin lambda variable 1-36 | IGL@ /// IGLV1-36 /// IGLV1-44 /// IL8 | chr22q11.1-q11.2 /// chr22q11.2 /// chr4q13-q21 |
| T cell receptor alpha locus | TRA@ | chr14q11.2 |
| Immunoglobulin lambda joining 3 | IGL@ | chr22q11.1-q11.2 |
| Enhancer of polycomb homolog 1 (Drosophila) /// (1.3) mRNA for T-cell receptor beta chain | IL23A | chr12q13.2 |
| Immunoglobulin lambda variable group /// Immunoglobulin lambda joining 3 | IGL@ /// RPL14 | chr22q11.1-q11.2 /// chr3p22-p21.2 |
| T-cell receptor ( V beta 17.1 variant, J beta 2.3, C beta 2) mRNA /// Enhancer of polycomb homolog 1 (Drosophila) /// Coiled-coil domain containing 42 /// T-cell receptor active beta-chain (V9-D-J-C) mRNA, clone PL2.6 /// TCR-beta VB12 subfamily gene, 5' end /// T-cell receptor V beta gene segment V-beta-w21, clone IGRb01 /// T-cell receptor rearranged alpha chain mRNA V-region (cell line B5.62) /// T-cell receptor rearranged beta-chain V-region (V-D-J) mRNA, clone ph32 /// T-cell receptor active beta-chain (V7-D-J-C) mRNA, clone PL4.19 | IFI6 /// IL23A | chr12q13.2 /// chr1p35 |
| YME1-like 1 (S. cerevisiae) /// T-cell antigen receptor-alpha (TCRA) mRNA, partial putative cds | TRA@ | chr14q11.2 |
| TAF4b RNA polymerase II, TATA box binding protein (TBP)-associated factor, 105kDa | TAF4B | chr18q11.2 |
| Mucin 4, cell surface associated | MUC4 | chr3q29 |
| TAF3 RNA polymerase II, TATA box binding protein (TBP)-associated factor, 140kDa | TAF3 | chr10p15.1 |
| TAF10 RNA polymerase II, TATA box binding protein (TBP)-associated factor, 30kDa | TAF10 | chr11p15.3 |
| TAF3 RNA polymerase II, TATA box binding protein (TBP)-associated factor, 140kDa | TAF3 | chr10p15.1 |
| TSC22 domain family, member 3 | TSC22D3 | chrXq22.3 |
| Mitogen-activated protein kinase kinase kinase 7 interacting protein 1 | MAP3K7IP1 | chr22q13.1 |
| SMAD family member 2 | SMAD2 | chr18q21.1 |
| SMAD family member 4 | SMAD4 | chr18q21.1 |
| thymic stromal lymphopoietin | TSLP | chr5q22.1 |
| protein kinase, cAMP-dependent, catalytic, beta | PRKACB | chr1p36.1 |
| phosphoinositide-3-kinase, class 2, alpha polypeptide | PIK3C2A | chr11p15.5-p14 |
| mitogen-activated protein kinase kinase kinase 7 interacting protein 1 | MAP3K7IP1 | chr22q13.1 |
| GNAS complex locus | GNAS | chr20q13.3 |
| CREB binding protein (Rubinstein-Taybi syndrome) | CREBBP | chr16p13.3 |
| TAF10 RNA polymerase II, TATA box binding protein (TBP)-associated factor, 30kDa | TAF10 | chr11p15.3 |
| fatty acid binding protein 4, adipocyte | FABP4 | chr8q21 |
| Phosphoinositide-3-kinase, catalytic, alpha polypeptide | PIK3CA | chr3q26.3 |
| zinc finger protein 91 | ZNF91 | chr19p13.1-p12 |
| leukotriene B4 receptor | LTB4R | chr14q11.2-q12 |
| major histocompatibility complex, class II, DQ alpha 1 | HLA-DQA1 | chr6p21.3 |
| Syntaxin 4 | STX4 | chr16p11.2 |
| nuclear factor of activated T-cells, cytoplasmic, calcineurin-dependent 4 | NFATC4 | chr14q11.2 |
| protein tyrosine phosphatase, non-receptor type 22 (lymphoid) | PTPN22 | chr1p13.3-p13.1 |
| Tyrosine 3-monooxygenase/tryptophan 5-monooxygenase activation protein, eta polypeptide | YWHAH | chr22q12.3 |
| Transforming growth factor, beta receptor I (activin A receptor type II-like kinase, 53kDa) | TGFBR1 | chr9q22 |
| chromosome 16 open reading frame 77 | C16orf77 | chr16q22.1 |
| cAMP responsive element binding protein 1 | CREB1 | chr2q34 |
| adducin 2 (beta) | ADD2 | chr2p14-p13 |
| Son of sevenless homolog 2 (Drosophila) | SOS2 | chr14q21 |
| Proteasome (prosome, macropain) assembly chaperone 1 | PSMG1 | chr21q22.3 |
| major histocompatibility complex, class II, DQ beta 1 /// major histocompatibility complex, class II, DQ beta 2 /// major histocompatibility complex, class II, DR beta 1 /// major histocompatibility complex, class II, DR beta 2 (pseudogene) /// major histocompatibility complex, class II, DR beta 3 /// major histocompatibility complex, class II, DR beta 4 /// major histocompatibility complex, class II, DR beta 5 /// ribonuclease, RNase A family, 2 (liver, eosinophil-derived neurotoxin) /// zinc finger protein 749 /// hypothetical protein LOC730415 /// similar to Major histocompatibility complex, class II, DR beta 4 /// similar to major histocompatibility complex, class II, DQ beta 1 /// similar to HLA class II histocompatibility antigen, DR-W53 beta chain /// similar to hCG1992647 | hCG_1998957 /// HLA-DQB1 /// HLA-DQB2 /// HLA-DRB1 /// HLA-DRB2 /// HLA-DRB3 /// HLA-DRB4 /// HLA-DRB5 /// LOC100133484 /// LOC100133583 /// LOC100133661 /// LOC100133811 /// LOC730415 /// RNASE2 /// ZNF749 | chr14q24-q31 /// chr19q13.43 /// chr6p21 /// chr6p21.3 |
| GNAS complex locus | GNAS | chr20q13.3 |
| SMAD family member 2 | SMAD2 | chr18q21.1 |
| interleukin 12 receptor, beta 1 | IL12RB1 | chr19p13.1 |
| P300/CBP-associated factor | PCAF | chr3p24 |
| Janus kinase 1 (a protein tyrosine kinase) | JAK1 | chr1p32.3-p31.3 |
| 3-hydroxy-3-methylglutaryl-Coenzyme A synthase 2 (mitochondrial) | HMGCS2 | chr1p13-p12 |
| Janus kinase 1 (a protein tyrosine kinase) | JAK1 | chr1p32.3-p31.3 |
| Enhancer of polycomb homolog 1 (Drosophila) | IL23A | chr12q13.2 |
| esterase D/formylglutathione hydrolase | ESD | chr13q14.1-q14.2 |
| Aldolase B, fructose-bisphosphate | ALDOB | chr9q21.3-q22.2 |
| phosphoinositide-3-kinase, class 3 | PIK3C3 | chr18q12.3 |
| Protein tyrosine phosphatase, non-receptor type 2 | PTPN2 | chr18p11.3-p11.2 |
| protein tyrosine phosphatase, non-receptor type 2 | PTPN2 | chr18p11.3-p11.2 |
| Phosphoinositide-3-kinase, class 2, alpha polypeptide | PIK3C2A | chr11p15.5-p14 |
| Protein tyrosine phosphatase, non-receptor type 2 | PTPN2 | chr18p11.3-p11.2 |
| Tyrosine 3-monooxygenase/tryptophan 5-monooxygenase activation protein, eta polypeptide | YWHAH | chr22q12.3 |
| General transcription factor IIH, polypeptide 1, 62kDa | GTF2H1 | chr11p15.1-p14 |
| Interleukin 4 receptor | IL4R | chr16p12.1-p11.2 |
| chromosome 11 open reading frame 30 | C11orf30 | chr11q13.5 |
| interferon gamma receptor 1 | IFNGR1 | chr6q23.3 |
| signal transducer and activator of transcription 3 (acute-phase response factor) | STAT3 | chr17q21.31 |
| cytochrome P450, family 3, subfamily A, polypeptide 7 | CYP3A7 | chr7q21-q22.1 |
| aldolase B, fructose-bisphosphate | ALDOB | chr9q21.3-q22.2 |
| ATP-binding cassette, sub-family B (MDR/TAP), member 1 | ABCB1 | chr7q21.1 |
| interleukin 6 (interferon, beta 2) /// hypothetical LOC541472 | IL6 /// LOC541472 | chr7p21 |
| general transcription factor IIA, 2, 12kDa | GTF2A2 | chr15q22.2 |
| POU class 2 homeobox 1 | POU2F1 | chr1q22-q23 |
| butyrophilin, subfamily 2, member A1 | BTN2A1 | chr6p22.1 |
| interleukin 28 receptor, alpha (interferon, lambda receptor) | IL28RA | chr1p36.11 |
| protein phosphatase 3 (formerly 2B), regulatory subunit B, beta isoform | PPP3R2 | chr9q31.1 |
| Protein phosphatase 3 (formerly 2B), catalytic subunit, gamma isoform | PPP3CC | chr8p21.3 |
| protein phosphatase 3 (formerly 2B), catalytic subunit, gamma isoform | PPP3CC | chr8p21.3 |
| thromboxane A2 receptor | TBXA2R | chr19p13.3 |
| mitogen-activated protein kinase 7 | MAPK7 | chr17p11.2 |
| interleukin 1, beta | IL1B | chr2q14 |
| peptidase inhibitor 3, skin-derived (SKALP) | PI3 | chr20q12-q13 |
| zinc finger, CCCH-type with G patch domain | ZGPAT | chr20q13.3 |
| signal transducer and activator of transcription 1, 91kDa | STAT1 | chr2q32.2 |
| signal transducer and activator of transcription 1, 91kDa | STAT1 | chr2q32.2 |
| signal transducer and activator of transcription 1, 91kDa | STAT1 | chr2q32.2 |
| signal transducer and activator of transcription 1, 91kDa | STAT1 | chr2q32.2 |
