# Supplementary material for: Gene Expression Analysis of Peripheral Cells for Subclassification of Pediatric Inflammatory Bowel Disease in Remission
Source: PLoS One. 2013 Nov 18;8(11):e79549. doi: 10.1371/journal.pone.0079549 (PMC3832619; doi:10.1371/journal.pone.0079549)
Supplement: Table S3 — Top genes up/down regulated Group A/B/C vs Control. (DOC) [file pone.0079549.s003.doc]

**Table S3, Top genes up/down regulated Group A/B/C vs Control**

|  | **Top Up** |  |  |  | **Top Down** |  |  |
| --- | --- | --- | --- | --- | --- | --- | --- |
| **A vs Control** | **Symbol** | **Entrez Gene Name** | **Log Ratio** |  | **Symbol** | **Entrez Gene Name** | **Log Ratio** |
|  | BCL2L1 | BCL2-like 1 | 1,122 |  | NFATC2 | nuclear factor of activated T-cells, cytoplasmic, calcineurin-dependent 2 | -1,072 |
|  | FCGR1A | Fc fragment of IgG, high affinity Ia, receptor (CD64) | 0,799 |  | TRA@ | T cell receptor alpha locus | -0,931 |
|  | MUC5AC | mucin 5AC, oligomeric mucus/gel-forming | 0,716 |  | TRD@ | T cell receptor delta locus | -0,908 |
|  | SOD2 | superoxide dismutase 2, mitochondrial | 0,715 |  | CD3G | CD3g molecule, gamma (CD3-TCR complex) | -0,889 |
|  | CSTA | cystatin A (stefin A) | 0,634 |  | GNAS | GNAS complex locus | -0,878 |
|  | PIK3C3 | phosphoinositide-3-kinase, class 3 | 0,631 |  | SLC26A2 | solute carrier family 26 (sulfate transporter), member 2 | -0,864 |
|  | SOS2 | son of sevenless homolog 2 (Drosophila) | 0,620 |  | PIK3C2A | phosphoinositide-3-kinase, class 2, alpha polypeptide | -0,845 |
|  |  |  |  |  | C11ORF30 | chromosome 11 open reading frame 30 | -0,839 |
|  |  |  |  |  | HLA-DQA1 | major histocompatibility complex, class II, DQ alpha 1 | -0,825 |
|  |  |  |  |  | ZNF91 | zinc finger protein 91 | -0,790 |
|  |  |  |  |  | NFATC3 | nuclear factor of activated T-cells, cytoplasmic, calcineurin-dependent 3 | -0,769 |
|  |  |  |  |  | CCL5 | chemokine (C-C motif) ligand 5 | -0,760 |
|  |  |  |  |  | EP300 | E1A binding protein p300 | -0,759 |
|  |  |  |  |  | TAF3 | TAF3 RNA polymerase II, TATA box binding protein (TBP)-associated factor, 140kDa | -0,745 |
|  |  |  |  |  | MUC8 | mucin 8 | -0,699 |
|  |  |  |  |  | CD247 | CD247 molecule | -0,663 |
|  |  |  |  |  | TGFBR1 | transforming growth factor, beta receptor 1 | -0,645 |
|  |  |  |  |  | CCR5 | chemokine (C-C motif) receptor 5 | -0,609 |
|  |  |  |  |  | SMARCA4 | SWI/SNF related, matrix associated, actin dependent regulator of chromatin, subfamily a, member 4 | -0,606 |
|  |  |  |  |  | KAT2B | K(lysine) acetyltransferase 2B | -0,597 |
|  |  |  |  |  | CUX1 | cut-like homeobox 1 | -0,591 |
|  |  |  |  |  | SLC20A1 | solute carrier family 20 (phosphate transporter), member 1 | -0,589 |
|  |  |  |  |  |  |  |  |
|  |  |  |  |  |  |  |  |
|  | **Top Up** |  |  |  | **Top Down** |  |  |
| **B vs Control** | **Symbol** | **Entrez Gene Name** | **Log Ratio** |  | **Symbol** | **Entrez Gene Name** | **Log Ratio** |
|  | POLR2K | polymerase (RNA) II (DNA directed) polypeptide K, 7.0kDa | 2,424 |  | HLA-DQA1 | major histocompatibility complex, class II, DQ alpha 1 | -1,745 |
|  | IL8 | interleukin 8 | 2,352 |  | HLA-DQB1 | major histocompatibility complex, class II, DQ beta 1 | -0,882 |
|  | IFI44 | interferon-induced protein 44 | 2,254 |  | NFATC2 | nuclear factor of activated T-cells, cytoplasmic, calcineurin-dependent 2 | -0,870 |
|  | FCGR1A | Fc fragment of IgG, high affinity Ia, receptor (CD64) | 2,062 |  | MUC8 | mucin 8 | -0,790 |
|  | CSTA | cystatin A (stefin A) | 2,018 |  | CCL5 | chemokine (C-C motif) ligand 5 | -0,756 |
|  | IFNGR1 | interferon gamma receptor 1 | 1,857 |  | GNAS | GNAS complex locus | -0,726 |
|  | ANXA1 | annexin A1 | 1,815 |  | NFATC3 | nuclear factor of activated T-cells, cytoplasmic, calcineurin-dependent 3 | -0,714 |
|  | HMGB1 (includes EG:3146) | high-mobility group box 1 | 1,786 |  | POU2F1 | POU class 2 homeobox 1 | -0,712 |
|  | NAT1 | N-acetyltransferase 1 (arylamine N-acetyltransferase) | 1,752 |  | SMARCA4 | SWI/SNF related, matrix associated, actin dependent regulator of chromatin, subfamily a, member 4 | -0,698 |
|  | PIK3C2A | phosphoinositide-3-kinase, class 2, alpha polypeptide | 1,665 |  | MAP2K7 | mitogen-activated protein kinase kinase 7 | -0,692 |
|  | LRRK2 | leucine-rich repeat kinase 2 | 1,526 |  | C11ORF30 | chromosome 11 open reading frame 30 | -0,662 |
|  | TGFBR1 | transforming growth factor, beta receptor 1 | 1,515 |  | ATG16L1 | ATG16 autophagy related 16-like 1 (S. cerevisiae) | -0,647 |
|  | IL15 | interleukin 15 | 1,510 |  | STAT3 | signal transducer and activator of transcription 3 (acute-phase response factor) | -0,633 |
|  | ATP2B1 | ATPase, Ca++ transporting, plasma membrane 1 | 1,460 |  | NCOR2 | nuclear receptor co-repressor 2 | -0,615 |
|  | JAK2 | Janus kinase 2 | 1,440 |  | NFATC1 | nuclear factor of activated T-cells, cytoplasmic, calcineurin-dependent 1 | -0,600 |
|  | TAF7 | TAF7 RNA polymerase II, TATA box binding protein (TBP)-associated factor, 55kDa | 1,428 |  | NCOA1 | nuclear receptor coactivator 1 | -0,592 |
|  | TLR4 | toll-like receptor 4 | 1,390 |  | TAF11 | TAF11 RNA polymerase II, TATA box binding protein (TBP)-associated factor, 28kDa | -0,587 |
|  | PTPN22 | protein tyrosine phosphatase, non-receptor type 22 (lymphoid) | 1,388 |  |  |  |  |
|  | NRIP1 | nuclear receptor interacting protein 1 | 1,372 |  |  |  |  |
|  | PRKACB | protein kinase, cAMP-dependent, catalytic, beta | 1,336 |  |  |  |  |
|  | MAP3K7 | mitogen-activated protein kinase kinase kinase 7 | 1,332 |  |  |  |  |
|  | HSP90AA1 | heat shock protein 90kDa alpha (cytosolic), class A member 1 | 1,308 |  |  |  |  |
|  | TAF9 | TAF9 RNA polymerase II, TATA box binding protein (TBP)-associated factor, 32kDa | 1,295 |  |  |  |  |
|  | ZNF91 | zinc finger protein 91 | 1,287 |  |  |  |  |
|  | SRGN | serglycin | 1,263 |  |  |  |  |
|  | MAPK6 | mitogen-activated protein kinase 6 | 1,248 |  |  |  |  |
|  | SLC26A2 | solute carrier family 26 (sulfate transporter), member 2 | 1,246 |  |  |  |  |
|  | S100P | S100 calcium binding protein P | 1,209 |  |  |  |  |
|  | NR3C1 | nuclear receptor subfamily 3, group C, member 1 (glucocorticoid receptor) | 1,202 |  |  |  |  |
|  | NRAS | neuroblastoma RAS viral (v-ras) oncogene homolog | 1,197 |  |  |  |  |
|  | LYZ | lysozyme (renal amyloidosis) | 1,191 |  |  |  |  |
|  | KAT2B | K(lysine) acetyltransferase 2B | 1,171 |  |  |  |  |
|  | HSPA14 | heat shock 70kDa protein 14 | 1,139 |  |  |  |  |
|  | STAT1 | signal transducer and activator of transcription 1, 91kDa | 1,124 |  |  |  |  |
|  | CREB1 | cAMP responsive element binding protein 1 | 1,122 |  |  |  |  |
|  | HLTF | helicase-like transcription factor | 1,119 |  |  |  |  |
|  | GTF2B | general transcription factor IIB | 1,050 |  |  |  |  |
|  | SLPI | secretory leukocyte peptidase inhibitor | 1,001 |  |  |  |  |
|  | TAF2 | TAF2 RNA polymerase II, TATA box binding protein (TBP)-associated factor, 150kDa | 0,953 |  |  |  |  |
|  | ACAT1 | acetyl-Coenzyme A acetyltransferase 1 | 0,948 |  |  |  |  |
|  | NCOA2 | nuclear receptor coactivator 2 | 0,947 |  |  |  |  |
|  | PTGER4 | prostaglandin E receptor 4 (subtype EP4) | 0,934 |  |  |  |  |
|  | RRAS2 | related RAS viral (r-ras) oncogene homolog 2 | 0,912 |  |  |  |  |
|  | GTF2H5 | general transcription factor IIH, polypeptide 5 | 0,910 |  |  |  |  |
|  | GTF2A1 | general transcription factor IIA, 1, 19/37kDa | 0,909 |  |  |  |  |
|  | POLR2B | polymerase (RNA) II (DNA directed) polypeptide B, 140kDa | 0,897 |  |  |  |  |
|  | MAPK8 | mitogen-activated protein kinase 8 | 0,887 |  |  |  |  |
|  | PPP3CA | protein phosphatase 3 (formerly 2B), catalytic subunit, alpha isoform | 0,887 |  |  |  |  |
|  | SMAD4 | SMAD family member 4 | 0,862 |  |  |  |  |
|  | MED14 | mediator complex subunit 14 | 0,838 |  |  |  |  |
|  | GTF2E1 | general transcription factor IIE, polypeptide 1, alpha 56kDa | 0,831 |  |  |  |  |
|  | TAF5 | TAF5 RNA polymerase II, TATA box binding protein (TBP)-associated factor, 100kDa | 0,827 |  |  |  |  |
|  | PTPN2 | protein tyrosine phosphatase, non-receptor type 2 | 0,826 |  |  |  |  |
|  | TAF9B | TAF9B RNA polymerase II, TATA box binding protein (TBP)-associated factor, 31kDa | 0,826 |  |  |  |  |
|  | SOD2 | superoxide dismutase 2, mitochondrial | 0,809 |  |  |  |  |
|  | TAF1 | TAF1 RNA polymerase II, TATA box binding protein (TBP)-associated factor, 250kDa | 0,796 |  |  |  |  |
|  | HIF1A | hypoxia inducible factor 1, alpha subunit (basic helix-loop-helix transcription factor) | 0,749 |  |  |  |  |
|  | C13ORF31 | chromosome 13 open reading frame 31 | 0,748 |  |  |  |  |
|  | TLR1 | toll-like receptor 1 | 0,738 |  |  |  |  |
|  | HLA-DRA | major histocompatibility complex, class II, DR alpha | 0,728 |  |  |  |  |
|  | S100A9 | S100 calcium binding protein A9 | 0,723 |  |  |  |  |
|  | ISG15 | ISG15 ubiquitin-like modifier | 0,721 |  |  |  |  |
|  | FEZ2 | fasciculation and elongation protein zeta 2 (zygin II) | 0,715 |  |  |  |  |
|  | WDR92 | WD repeat domain 92 | 0,712 |  |  |  |  |
|  | SRI | sorcin | 0,711 |  |  |  |  |
|  | MAP2K4 | mitogen-activated protein kinase kinase 4 | 0,704 |  |  |  |  |
|  | PIK3CA | phosphoinositide-3-kinase, catalytic, alpha polypeptide | 0,701 |  |  |  |  |
|  | IFI16 | interferon, gamma-inducible protein 16 | 0,699 |  |  |  |  |
|  | SMAD2 | SMAD family member 2 | 0,697 |  |  |  |  |
|  | BTN2A1 | butyrophilin, subfamily 2, member A1 | 0,690 |  |  |  |  |
|  | GTF2H2 | general transcription factor IIH, polypeptide 2, 44kDa | 0,687 |  |  |  |  |
|  | SLC22A4 (includes EG:6583) | solute carrier family 22 (organic cation/ergothioneine transporter), member 4 | 0,681 |  |  |  |  |
|  | SOS2 | son of sevenless homolog 2 (Drosophila) | 0,665 |  |  |  |  |
|  | TAF12 (includes EG:6883) | TAF12 RNA polymerase II, TATA box binding protein (TBP)-associated factor, 20kDa | 0,664 |  |  |  |  |
|  | FOS | v-fos FBJ murine osteosarcoma viral oncogene homolog | 0,661 |  |  |  |  |
|  | SUMO1 | SMT3 suppressor of mif two 3 homolog 1 (S. cerevisiae) | 0,655 |  |  |  |  |
|  | HLA-DRB1 | major histocompatibility complex, class II, DR beta 1 | 0,653 |  |  |  |  |
|  | FGFR1OP (includes EG:11116) | FGFR1 oncogene partner | 0,646 |  |  |  |  |
|  | MAPK14 | mitogen-activated protein kinase 14 | 0,642 |  |  |  |  |
|  | KRAS | v-Ki-ras2 Kirsten rat sarcoma viral oncogene homolog | 0,631 |  |  |  |  |
|  | TAF1A | TATA box binding protein (TBP)-associated factor, RNA polymerase I, A, 48kDa | 0,630 |  |  |  |  |
|  | SUMO4 | SMT3 suppressor of mif two 3 homolog 4 (S. cerevisiae) | 0,627 |  |  |  |  |
|  | PPP3CB | protein phosphatase 3 (formerly 2B), catalytic subunit, beta isoform | 0,615 |  |  |  |  |
|  | CCNH | cyclin H | 0,611 |  |  |  |  |
|  | PPP2CA | protein phosphatase 2 (formerly 2A), catalytic subunit, alpha isoform | 0,595 |  |  |  |  |
|  |  |  |  |  |  |  |  |
|  |  |  |  |  |  |  |  |
|  | **Top Up** |  |  |  | **Top Down** |  |  |
| **C vs Control** | **Symbol** | **Entrez Gene Name** | **Log Ratio** |  | **Symbol** | **Entrez Gene Name** | **Log Ratio** |
|  | FCGR1A | Fc fragment of IgG, high affinity Ia, receptor (CD64) | 2,126 |  | NFATC2 | nuclear factor of activated T-cells, cytoplasmic, calcineurin-dependent 2 | -1,459 |
|  | CXCL10 | chemokine (C-X-C motif) ligand 10 | 1,944 |  | NFATC3 | nuclear factor of activated T-cells, cytoplasmic, calcineurin-dependent 3 | -1,395 |
|  | CSTA | cystatin A (stefin A) | 1,810 |  | CD3G | CD3g molecule, gamma (CD3-TCR complex) | -1,225 |
|  | BCL2L1 | BCL2-like 1 | 1,578 |  | GNAS | GNAS complex locus | -1,219 |
|  | S100P | S100 calcium binding protein P | 1,522 |  | CCL5 | chemokine (C-C motif) ligand 5 | -1,141 |
|  | IL1R2 | interleukin 1 receptor, type II | 1,382 |  | C11ORF30 | chromosome 11 open reading frame 30 | -1,117 |
|  | KAT2B | K(lysine) acetyltransferase 2B | 1,278 |  | CD247 | CD247 molecule | -1,112 |
|  | CCL2 | chemokine (C-C motif) ligand 2 | 1,215 |  | PIK3C2A | phosphoinositide-3-kinase, class 2, alpha polypeptide | -1,081 |
|  | IFNGR1 | interferon gamma receptor 1 | 1,210 |  | SMARCA4 | SWI/SNF related, matrix associated, actin dependent regulator of chromatin, subfamily a, member 4 | -1,068 |
|  | IFI44 | interferon-induced protein 44 | 1,189 |  | TAF3 | TAF3 RNA polymerase II, TATA box binding protein (TBP)-associated factor, 140kDa | -1,025 |
|  | TLR5 | toll-like receptor 5 | 1,180 |  | TGFBR1 | transforming growth factor, beta receptor 1 | -1,008 |
|  | PBX1 | pre-B-cell leukemia homeobox 1 | 1,173 |  | TRA@ | T cell receptor alpha locus | -0,978 |
|  | SLC22A4 (includes EG:6583) | solute carrier family 22 (organic cation/ergothioneine transporter), member 4 | 1,167 |  | UBE2I | ubiquitin-conjugating enzyme E2I (UBC9 homolog, yeast) | -0,944 |
|  | POLR2K | polymerase (RNA) II (DNA directed) polypeptide K, 7.0kDa | 1,163 |  | EP300 | E1A binding protein p300 | -0,942 |
|  | CCL8 | chemokine (C-C motif) ligand 8 | 1,118 |  | SLC26A2 | solute carrier family 26 (sulfate transporter), member 2 | -0,922 |
|  | FKBP5 | FK506 binding protein 5 | 1,104 |  | NCOR2 | nuclear receptor co-repressor 2 | -0,916 |
|  | MAPK14 | mitogen-activated protein kinase 14 | 1,094 |  | SLC20A1 | solute carrier family 20 (phosphate transporter), member 1 | -0,916 |
|  | SRGN | serglycin | 1,087 |  | TAF11 | TAF11 RNA polymerase II, TATA box binding protein (TBP)-associated factor, 28kDa | -0,888 |
|  | ITLN1 | intelectin 1 (galactofuranose binding) | 1,078 |  | NFATC1 | nuclear factor of activated T-cells, cytoplasmic, calcineurin-dependent 1 | -0,883 |
|  | ANXA1 | annexin A1 | 1,061 |  | ZNF91 | zinc finger protein 91 | -0,874 |
|  | WDR92 | WD repeat domain 92 | 1,058 |  | TRD@ | T cell receptor delta locus | -0,861 |
|  | JAK2 | Janus kinase 2 | 1,010 |  | IL16 | interleukin 16 (lymphocyte chemoattractant factor) | -0,860 |
|  | LYZ | lysozyme (renal amyloidosis) | 0,994 |  | HLA-DQA1 | major histocompatibility complex, class II, DQ alpha 1 | -0,839 |
|  | CD163 | CD163 molecule | 0,972 |  | IGL@ | immunoglobulin lambda locus | -0,808 |
|  | S100A9 | S100 calcium binding protein A9 | 0,972 |  | HLA-DQB1 | major histocompatibility complex, class II, DQ beta 1 | -0,803 |
|  | TLR4 | toll-like receptor 4 | 0,965 |  | NCOA1 | nuclear receptor coactivator 1 | -0,803 |
|  | GYPB | glycophorin B (MNS blood group) | 0,960 |  | POU2F1 | POU class 2 homeobox 1 | -0,801 |
|  | IL1RN | interleukin 1 receptor antagonist | 0,942 |  | CFD | complement factor D (adipsin) | -0,798 |
|  | MMP9 | matrix metallopeptidase 9 (gelatinase B, 92kDa gelatinase, 92kDa type IV collagenase) | 0,869 |  | PIK3C2B | phosphoinositide-3-kinase, class 2, beta polypeptide | -0,792 |
|  | STAT1 | signal transducer and activator of transcription 1, 91kDa | 0,868 |  | C5ORF56 | chromosome 5 open reading frame 56 | -0,782 |
|  | NAT1 | N-acetyltransferase 1 (arylamine N-acetyltransferase) | 0,823 |  | HSP90AB1 | heat shock protein 90kDa alpha (cytosolic), class B member 1 | -0,767 |
|  | CA4 | carbonic anhydrase IV | 0,819 |  | MUC8 | mucin 8 | -0,763 |
|  | SLPI | secretory leukocyte peptidase inhibitor | 0,804 |  | MED1 | mediator complex subunit 1 | -0,762 |
|  | HMGB1 (includes EG:3146) | high-mobility group box 1 | 0,791 |  | IGH@ | immunoglobulin heavy locus | -0,743 |
|  | SELENBP1 | selenium binding protein 1 | 0,755 |  | CDKN1C | cyclin-dependent kinase inhibitor 1C (p57, Kip2) | -0,733 |
|  | MXI1 | MAX interactor 1 | 0,749 |  | CXCL5 | chemokine (C-X-C motif) ligand 5 | -0,712 |
|  | IL15 | interleukin 15 | 0,712 |  | SMAD4 | SMAD family member 4 | -0,701 |
|  | SOS2 | son of sevenless homolog 2 (Drosophila) | 0,712 |  | CUX1 | cut-like homeobox 1 | -0,698 |
|  | CHP | calcium binding protein P22 | 0,700 |  | HLA-DRB1 | major histocompatibility complex, class II, DR beta 1 | -0,698 |
|  | SOD2 | superoxide dismutase 2, mitochondrial | 0,694 |  | JAK1 | Janus kinase 1 | -0,679 |
|  | FHL2 | four and a half LIM domains 2 | 0,671 |  | PIK3CD | phosphoinositide-3-kinase, catalytic, delta polypeptide | -0,669 |
|  | MT1H | metallothionein 1H | 0,668 |  | NOLC1 | nucleolar and coiled-body phosphoprotein 1 | -0,668 |
|  | TAF7 | TAF7 RNA polymerase II, TATA box binding protein (TBP)-associated factor, 55kDa | 0,636 |  | RAC1 | ras-related C3 botulinum toxin substrate 1 (rho family, small GTP binding protein Rac1) | -0,665 |
|  | LRRK2 | leucine-rich repeat kinase 2 | 0,633 |  | GUSB | glucuronidase, beta | -0,661 |
|  | STAT5B | signal transducer and activator of transcription 5B | 0,632 |  | SMAD3 | SMAD family member 3 | -0,660 |
|  | GTF2H5 | general transcription factor IIH, polypeptide 5 | 0,631 |  | IL12RB1 | interleukin 12 receptor, beta 1 | -0,647 |
|  | MAP3K1 | mitogen-activated protein kinase kinase kinase 1 | 0,620 |  | MAP3K14 | mitogen-activated protein kinase kinase kinase 14 | -0,635 |
|  | IL18RAP | interleukin 18 receptor accessory protein | 0,606 |  | IL32 | interleukin 32 | -0,633 |
|  | TTLL3 | tubulin tyrosine ligase-like family, member 3 | 0,602 |  | HLA-DOA | major histocompatibility complex, class II, DO alpha | -0,628 |
|  |  |  |  |  | SMARCA2 | SWI/SNF related, matrix associated, actin dependent regulator of chromatin, subfamily a, member 2 | -0,627 |
|  |  |  |  |  | SPARC | secreted protein, acidic, cysteine-rich (osteonectin) | -0,622 |
|  |  |  |  |  | CREBBP | CREB binding protein | -0,621 |
|  |  |  |  |  | CCR5 | chemokine (C-C motif) receptor 5 | -0,618 |
|  |  |  |  |  | CD14 | CD14 molecule | -0,601 |
|  |  |  |  |  | SCAP | SREBF chaperone | -0,589 |
|  |  |  |  |  | POLR2A | polymerase (RNA) II (DNA directed) polypeptide A, 220kDa | -0,587 |
|  |  |  |  |  | ZGPAT | zinc finger, CCCH-type with G patch domain | -0,586 |
