# Supplementary material for: Gene Expression Analysis of Peripheral Cells for Subclassification of Pediatric Inflammatory Bowel Disease in Remission
Source: PLoS One. 2013 Nov 18;8(11):e79549. doi: 10.1371/journal.pone.0079549 (PMC3832619; doi:10.1371/journal.pone.0079549)
Supplement: Table S4 — Specific top genes up/down regulated group A/B/C. (DOC) [file pone.0079549.s004.doc]

**Table S4, Specific top genes up/down regulated group A/B/C**

|  | **Top Up** |  |  |  | **Top Down** |  |  |
| --- | --- | --- | --- | --- | --- | --- | --- |
| **A vs Control and Group B,C** | **Symbol** | **Entrez Gene Name** | **Log Ratio** |  | **Symbol** | **Entrez Gene Name** | **Log Ratio** |
|  | FCGR1A | Fc fragment of IgG, high affinity Ia, receptor (CD64) | 0,799 |  |  |  |  |
|  | CSTA | cystatin A (stefin A) | 0,634 |  |  |  |  |
|  |  |  |  |  |  |  |  |
|  |  |  |  |  |  |  |  |
|  | **Top Up** |  |  |  | **Top Down** |  |  |
| **B vs Control and Group A,C** | **Symbol** | **Entrez Gene Name** | **Log Ratio** |  | **Symbol** | **Entrez Gene Name** | **Log Ratio** |
|  | POLR2K | polymerase (RNA) II (DNA directed) polypeptide K, 7.0kDa | 2,424 |  | HLA-DQA1 | major histocompatibility complex, class II, DQ alpha 1 | -1,745 |
|  | IL8 | interleukin 8 | 2,352 |  |  |  |  |
|  | IFI44 | interferon-induced protein 44 | 2,254 |  |  |  |  |
|  | IFNGR1 | interferon gamma receptor 1 | 1,857 |  |  |  |  |
|  | ANXA1 | annexin A1 | 1,815 |  |  |  |  |
|  | HMGB1 (includes EG:3146) | high-mobility group box 1 | 1,786 |  |  |  |  |
|  | NAT1 | N-acetyltransferase 1 (arylamine N-acetyltransferase) | 1,752 |  |  |  |  |
|  | PIK3C2A | phosphoinositide-3-kinase, class 2, alpha polypeptide | 1,665 |  |  |  |  |
|  | LRRK2 | leucine-rich repeat kinase 2 | 1,526 |  |  |  |  |
|  | TGFBR1 | transforming growth factor, beta receptor 1 | 1,515 |  |  |  |  |
|  | IL15 | interleukin 15 | 1,510 |  |  |  |  |
|  | ATP2B1 | ATPase, Ca++ transporting, plasma membrane 1 | 1,460 |  |  |  |  |
|  | TAF7 | TAF7 RNA polymerase II, TATA box binding protein (TBP)-associated factor, 55kDa | 1,428 |  |  |  |  |
|  | PTPN22 | protein tyrosine phosphatase, non-receptor type 22 (lymphoid) | 1,388 |  |  |  |  |
|  | NRIP1 | nuclear receptor interacting protein 1 | 1,372 |  |  |  |  |
|  | PRKACB | protein kinase, cAMP-dependent, catalytic, beta | 1,336 |  |  |  |  |
|  | MAP3K7 | mitogen-activated protein kinase kinase kinase 7 | 1,332 |  |  |  |  |
|  | HSP90AA1 | heat shock protein 90kDa alpha (cytosolic), class A member 1 | 1,308 |  |  |  |  |
|  | TAF9 | TAF9 RNA polymerase II, TATA box binding protein (TBP)-associated factor, 32kDa | 1,295 |  |  |  |  |
|  | ZNF91 | zinc finger protein 91 | 1,287 |  |  |  |  |
|  | MAPK6 | mitogen-activated protein kinase 6 | 1,248 |  |  |  |  |
|  | SLC26A2 | solute carrier family 26 (sulfate transporter), member 2 | 1,246 |  |  |  |  |
|  | NR3C1 | nuclear receptor subfamily 3, group C, member 1 (glucocorticoid receptor) | 1,202 |  |  |  |  |
|  | NRAS | neuroblastoma RAS viral (v-ras) oncogene homolog | 1,197 |  |  |  |  |
|  | HSPA14 | heat shock 70kDa protein 14 | 1,139 |  |  |  |  |
|  | CREB1 | cAMP responsive element binding protein 1 | 1,122 |  |  |  |  |
|  | HLTF | helicase-like transcription factor | 1,119 |  |  |  |  |
|  | GTF2B | general transcription factor IIB | 1,050 |  |  |  |  |
|  | TAF2 | TAF2 RNA polymerase II, TATA box binding protein (TBP)-associated factor, 150kDa | 0,953 |  |  |  |  |
|  | ACAT1 | acetyl-Coenzyme A acetyltransferase 1 | 0,948 |  |  |  |  |
|  | PTGER4 | prostaglandin E receptor 4 (subtype EP4) | 0,934 |  |  |  |  |
|  | RRAS2 | related RAS viral (r-ras) oncogene homolog 2 | 0,912 |  |  |  |  |
|  | GTF2A1 | general transcription factor IIA, 1, 19/37kDa | 0,909 |  |  |  |  |
|  | POLR2B | polymerase (RNA) II (DNA directed) polypeptide B, 140kDa | 0,897 |  |  |  |  |
|  | MAPK8 | mitogen-activated protein kinase 8 | 0,887 |  |  |  |  |
|  | SMAD4 | SMAD family member 4 | 0,862 |  |  |  |  |
|  | MED14 | mediator complex subunit 14 | 0,838 |  |  |  |  |
|  | GTF2E1 | general transcription factor IIE, polypeptide 1, alpha 56kDa | 0,831 |  |  |  |  |
|  | TAF5 | TAF5 RNA polymerase II, TATA box binding protein (TBP)-associated factor, 100kDa | 0,827 |  |  |  |  |
|  | TAF9B | TAF9B RNA polymerase II, TATA box binding protein (TBP)-associated factor, 31kDa | 0,826 |  |  |  |  |
|  | PTPN2 | protein tyrosine phosphatase, non-receptor type 2 | 0,826 |  |  |  |  |
|  | TAF1 | TAF1 RNA polymerase II, TATA box binding protein (TBP)-associated factor, 250kDa | 0,796 |  |  |  |  |
|  | HLA-DRA | major histocompatibility complex, class II, DR alpha | 0,728 |  |  |  |  |
|  | FEZ2 | fasciculation and elongation protein zeta 2 (zygin II) | 0,715 |  |  |  |  |
|  | SRI | sorcin | 0,711 |  |  |  |  |
|  | PIK3CA | phosphoinositide-3-kinase, catalytic, alpha polypeptide | 0,701 |  |  |  |  |
|  | BTN2A1 | butyrophilin, subfamily 2, member A1 | 0,690 |  |  |  |  |
|  | TAF12 (includes EG:6883) | TAF12 RNA polymerase II, TATA box binding protein (TBP)-associated factor, 20kDa | 0,664 |  |  |  |  |
|  | HLA-DRB1 | major histocompatibility complex, class II, DR beta 1 | 0,653 |  |  |  |  |
|  | MAP2K4 | mitogen-activated protein kinase kinase 4 | 0,638 |  |  |  |  |
|  | TAF1A | TATA box binding protein (TBP)-associated factor, RNA polymerase I, A, 48kDa | 0,630 |  |  |  |  |
|  | SMAD2 | SMAD family member 2 | 0,606 |  |  |  |  |
|  | C11ORF30 | chromosome 11 open reading frame 30 | 0,605 |  |  |  |  |
|  | PPP2CA | protein phosphatase 2 (formerly 2A), catalytic subunit, alpha isoform | 0,595 |  |  |  |  |
|  |  |  |  |  |  |  |  |
|  |  |  |  |  |  |  |  |
|  | **Top Up** |  |  |  | **Top Down** |  |  |
| **C vs Control and Group A,B** | **Symbol** | **Entrez Gene Name** | **Log Ratio** |  | **Symbol** | **Entrez Gene Name** | **Log Ratio** |
|  | IL1R2 | interleukin 1 receptor, type II | 1,382 |  | HLA-DQA1 | major histocompatibility complex, class II, DQ alpha 1 | -0,839 |
|  | CCL2 | chemokine (C-C motif) ligand 2 | 1,215 |  | TRA@ | T cell receptor alpha locus | -0,978 |
|  | IFNGR1 | interferon gamma receptor 1 | 1,210 |  | NFATC3 | nuclear factor of activated T-cells, cytoplasmic, calcineurin-dependent 3 | -1,395 |
|  | IFI44 | interferon-induced protein 44 | 1,189 |  |  |  |  |
|  | TLR5 | toll-like receptor 5 | 1,180 |  |  |  |  |
|  | PBX1 | pre-B-cell leukemia homeobox 1 | 1,173 |  |  |  |  |
|  | POLR2K | polymerase (RNA) II (DNA directed) polypeptide K, 7.0kDa | 1,163 |  |  |  |  |
|  | FKBP5 | FK506 binding protein 5 | 1,104 |  |  |  |  |
|  | ITLN1 | intelectin 1 (galactofuranose binding) | 1,078 |  |  |  |  |
|  | ANXA1 | annexin A1 | 1,061 |  |  |  |  |
|  | CD163 | CD163 molecule | 0,972 |  |  |  |  |
|  | MMP9 | matrix metallopeptidase 9 (gelatinase B, 92kDa gelatinase, 92kDa type IV collagenase) | 0,869 |  |  |  |  |
|  | NAT1 | N-acetyltransferase 1 (arylamine N-acetyltransferase) | 0,823 |  |  |  |  |
|  | HMGB1 (includes EG:3146) | high-mobility group box 1 | 0,791 |  |  |  |  |
|  | MXI1 | MAX interactor 1 | 0,749 |  |  |  |  |
|  | TGFBR1 | transforming growth factor, beta receptor 1 | 0,749 |  |  |  |  |
|  | IL15 | interleukin 15 | 0,712 |  |  |  |  |
|  | TAF7 | TAF7 RNA polymerase II, TATA box binding protein (TBP)-associated factor, 55kDa | 0,636 |  |  |  |  |
|  | LRRK2 | leucine-rich repeat kinase 2 | 0,633 |  |  |  |  |
