# Supplementary material for: Gene Expression Analysis of Peripheral Cells for Subclassification of Pediatric Inflammatory Bowel Disease in Remission
Source: PLoS One. 2013 Nov 18;8(11):e79549. doi: 10.1371/journal.pone.0079549 (PMC3832619; doi:10.1371/journal.pone.0079549)
Supplement: Table S5 — Supervised analysis. (DOC) [file pone.0079549.s005.doc]

**Table S5, Supervised analysis**

| **CD exacerbation vs non-exacerbation** |  |  |
| --- | --- | --- |
| **Gene Title** | **Symbol** | **Log Ratio** |
| chemokine (C-C motif) ligand 3 | CCL3 | -1,375 |
| interleukin 8 | IL8 | -1,293 |
| phosphoinositide-3-kinase, class 2, alpha polypeptide | PIK3C2A | -1,040 |
| complement factor D (adipsin) | CFD | -0,984 |
| protein tyrosine phosphatase, non-receptor type 22 (lymphoid) | PTPN22 | -0,941 |
| prostaglandin E receptor 4 (subtype EP4) | PTGER4 | -0,849 |
| major histocompatibility complex, class II, DQ alpha 1 /// major histocompatibility complex, class II, DQ alpha 2 | HLA-DQA1 /// HLA-DQA2 | -0,767 |
| polymerase (RNA) II (DNA directed) polypeptide B, 140kDa | POLR2B | -0,745 |
| heat shock protein 90kDa alpha (cytosolic), class A member 1 | HSP90AA1 | -0,739 |
| T cell receptor alpha locus /// T cell receptor delta locus | TRA@ /// TRD@ | -0,718 |
| TAF2 RNA polymerase II, TATA box binding protein (TBP)-associated factor, 150kDa | TAF2 | -0,687 |
| T cell receptor alpha locus /// T cell receptor delta locus | TRA@ /// TRD@ | -0,666 |
| heat shock protein 90kDa alpha (cytosolic), class A member 1 | HSP90AA1 | -0,644 |
| interleukin 15 | IL15 | -0,644 |
| high-mobility group box 1 | HMGB1 | -0,627 |
| TAF1 RNA polymerase II, TATA box binding protein (TBP)-associated factor, 250kDa | TAF1 | -0,597 |
| helicase-like transcription factor | HLTF | -0,596 |
|  |  |  |
|  |  |  |
| **CD vs UC** |  |  |
| **Gene Title** | **Symbol** | **Log Ratio** |
| Immunoglobulin lambda joining 3 | IGL@ | -0,731 |
| general transcription factor IIH, polypeptide 5 | GTF2H5 | -0,675 |
| son of sevenless homolog 2 (Drosophila) | SOS2 | -0,587 |
|  |  |  |
|  |  |  |
| **CD ileitis vs non-ileitis** |  |  |
| **Gene Title** | **Symbol** | **Log Ratio** |
| major histocompatibility complex, class II, DQ alpha 1 | HLA-DQA1 | -1,258 |
| son of sevenless homolog 1 (Drosophila) | SOS1 | -1,130 |
| immunoglobulin lambda locus /// interleukin 8 | IGL@ | -1,095 |
| immunoglobulin lambda locus | IGL@ | -0,971 |
| Immunoglobulin lambda joining 3 | IGL@ | -0,956 |
| immunoglobulin lambda locus | IGL@ | -0,951 |
| immunoglobulin heavy locus | IGH@ | -0,891 |
| immunoglobulin lambda locus | IGL@ | -0,875 |
| CD9 molecule | CD9 | -0,753 |
| immunoglobulin lambda locus | IGL@ | -0,735 |
| immunoglobulin lambda-like polypeptide 3 | IGLL3 | -0,726 |
| immunoglobulin lambda locus | IGH@ | -0,672 |
| TATA box binding protein (TBP)-associated factor, RNA polymerase I, A, 48kDa | TAF1A | -0,666 |
| immunoglobulin heavy constant alpha 1 | IGHA1 | -0,641 |
| Immunoglobulin lambda joining 3 | IGL@ | -0,636 |
| polymerase (RNA) II (DNA directed) polypeptide J | LOC100134053 | -0,599 |
| TSC22 domain family, member 3 | TSC22D3 | -0,588 |
